# Supplementary material for: Codon-optimized Npc1 mRNA corrects Niemann-Pick type C1 disease phenotypes in vitro and in vivo
Source: Mol Ther Nucleic Acids. 2026 Jul 18;37(3):103022. doi: 10.1016/j.omtn.2026.103022 (PMC13521171; doi:10.1016/j.omtn.2026.103022)
Supplement: Supplementary file 1 [file mmc1.pdf]

OMTN, Volume *37*

Supplemental information

Codon-optimized *Npc1* mRNA corrects Niemann-Pick type C1 disease phenotypes *in vitro* and *in vivo*

Ellen R. Koufer, Alice Townsend, Youngseo Na, Adele B. Correia, Noah K. Latham, Koralege C. Pathmasiri, Olga Ilnytska, Judith Storch, Kyle A. Sullivan, Troy Halseth, Thaddeus J. Kunkel, Elizabeth S. Phillippi, Matthew L. Lieberman, Stephanie M. Cologna, Andrew P. Lieberman, Daniel A. Jacobson, Anna S. Schwendeman, and Mark L. Schultz

Supplemental Figures

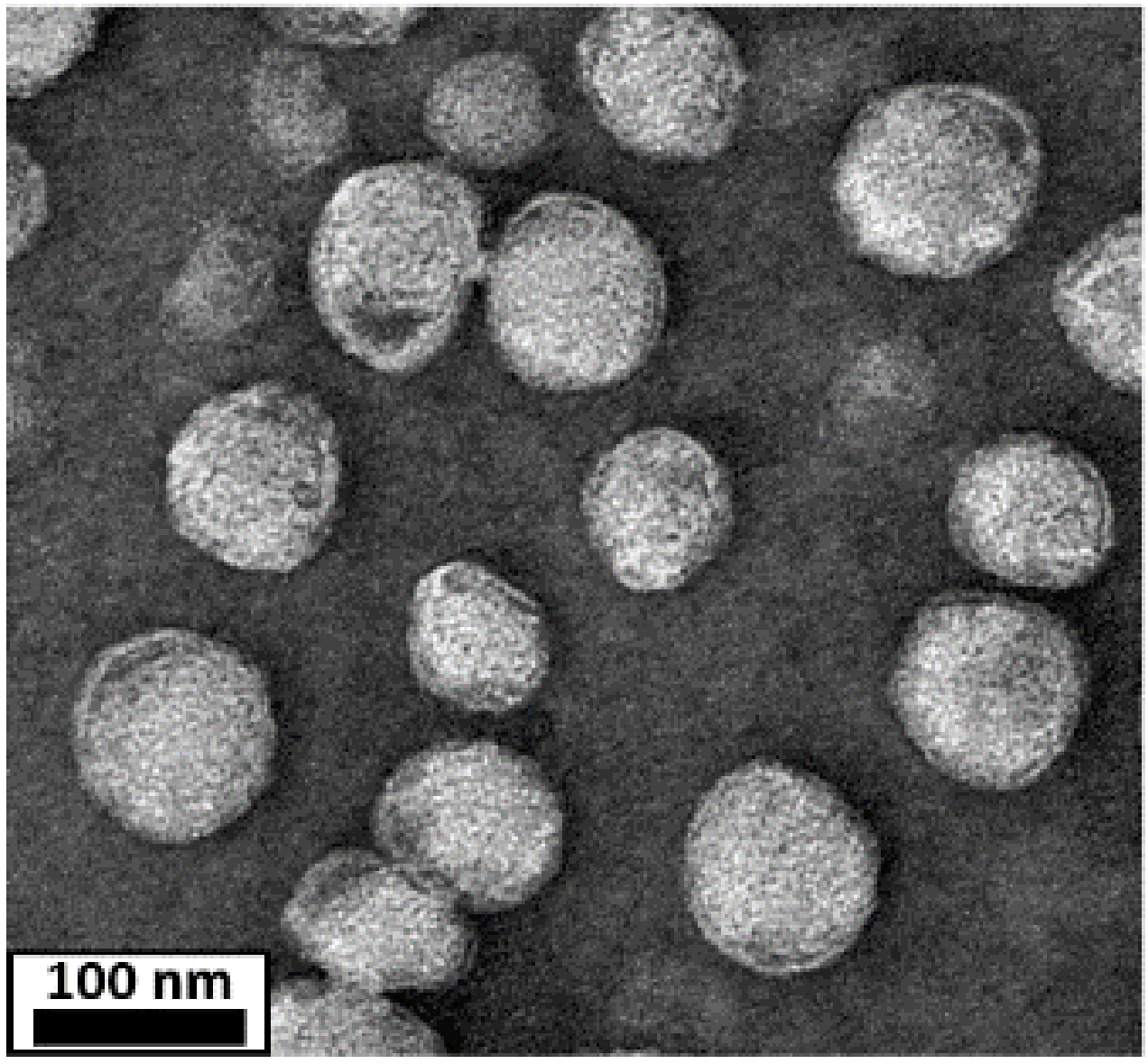

Figure S1. Transmission electron microscopy showing *GFP*:LNP particle diameter. Scale bar = 100 nm.

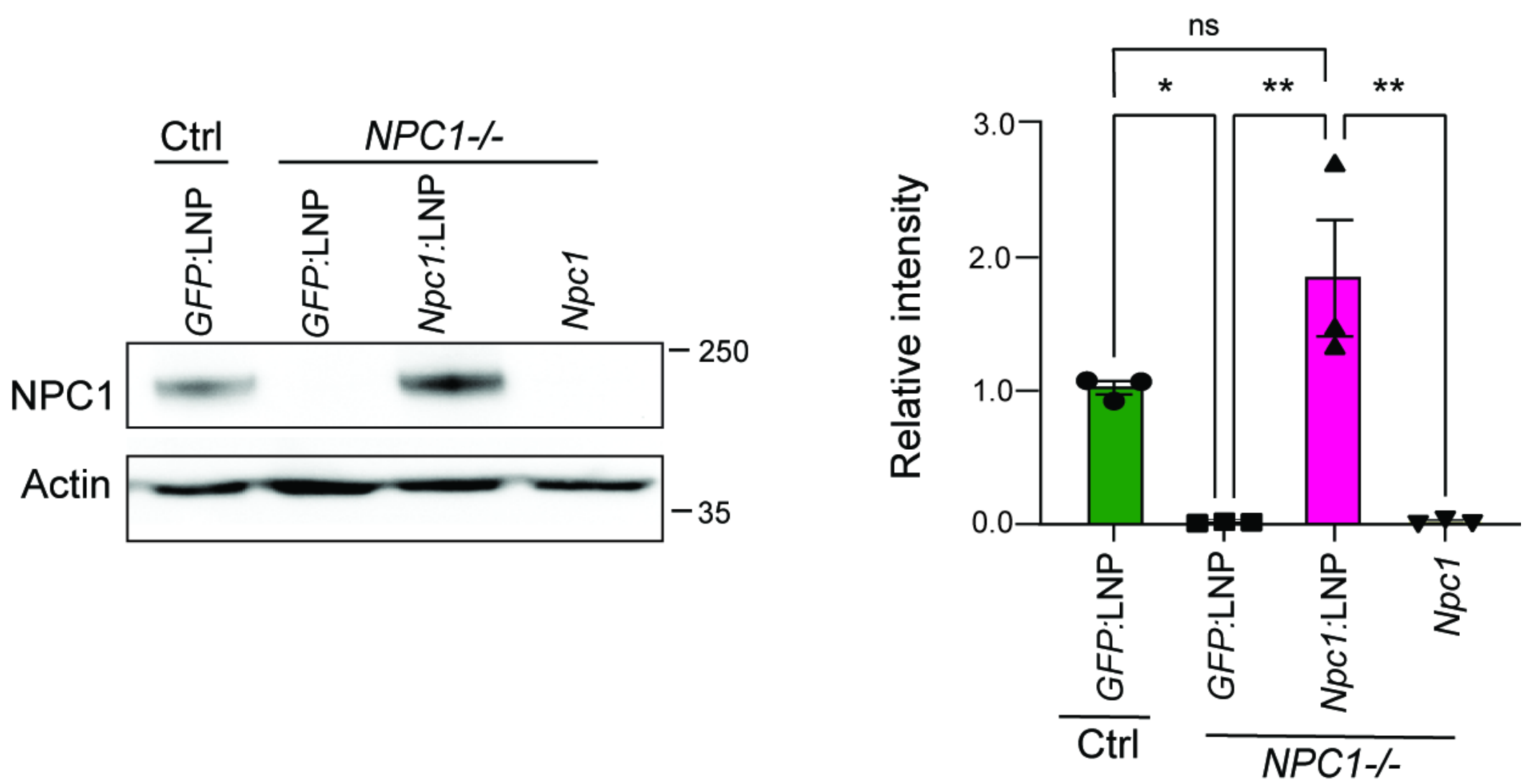

Figure S2. Total protein western blot for NPC1 after *Npc1*:LNP delivery. HeLa cells homozygous for null NPC1 (*NPC1*$^{-/-}$) or WT NPC1 (Ctrl) were treated for 24 hours with 1 µg lipid particle containing *GFP* or *Npc1* mRNA (*GFP*:LNP, *Npc1*:LNP), or 'naked' *Npc1* mRNA (not encapsulated within an LNP). After the 24-hour treatment, cells were given fresh media and analyzed 24 hours later by western blot for NPC1 protein levels. ns, not significant, *$p \leq .05$, **$p \leq .01$, ****$p \leq .0001$ by one-way ANOVA with Tukey's multiple comparisons test: F (3, 8) = 16.39. Data are shown as the mean ± SEM from n = 3/group.

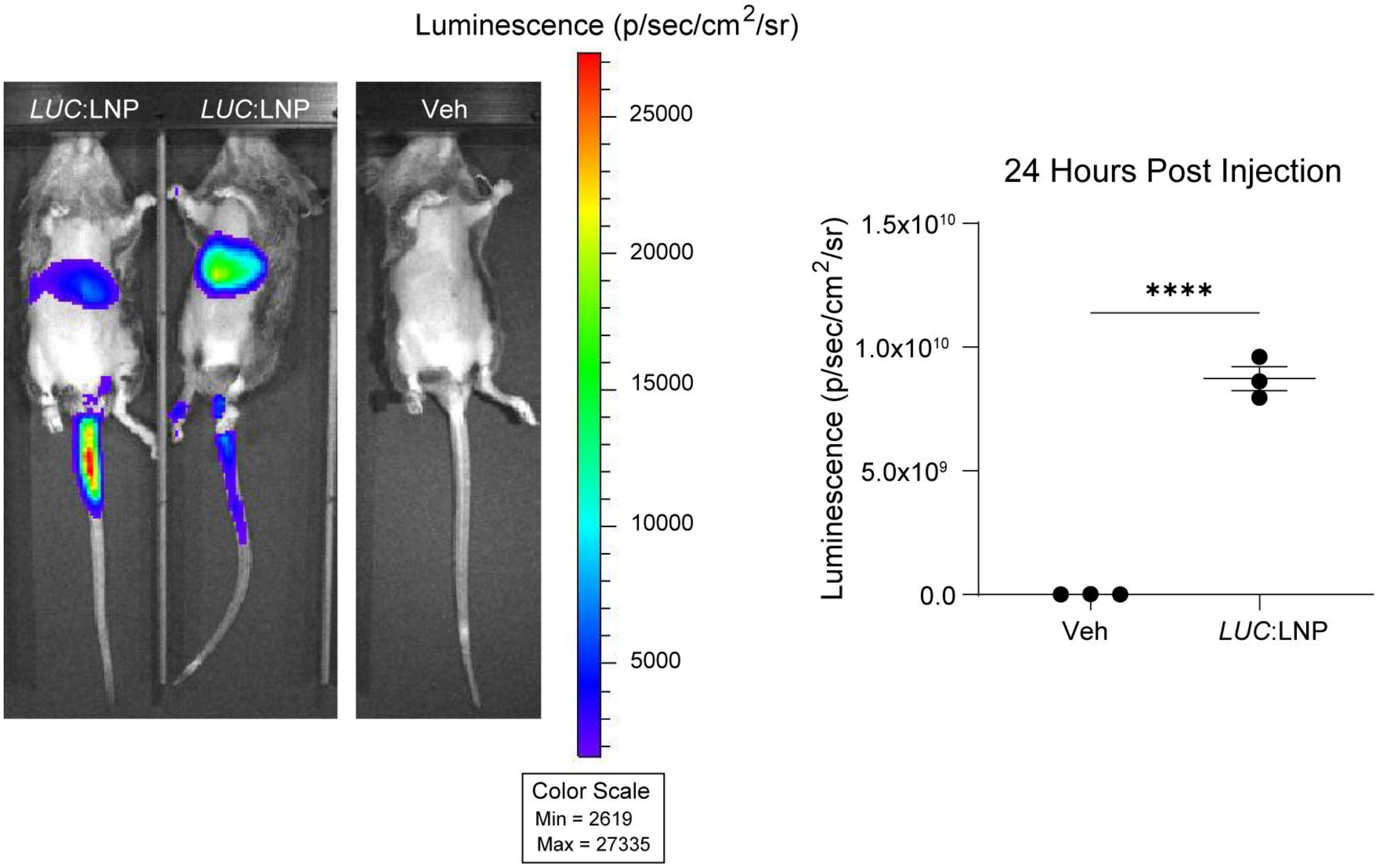

Figure S3. Image from the PerkinElmer IVIS Lumina optical imaging system showing localization of bioluminescent activity after LNP injection. Mice were injected once intravenously with 200 µL saline or 1 mg/kg of LNP containing luciferase mRNA (*LUC*:LNP). 24 hours after injection, mice were injected with 15 mg/kg d-luciferin and imaging was performed 20 minutes later. ****$p \leq 0.0001$ by unpaired t test: ($t=18.09$, $df=4$). Data are shown as the mean ± SEM from n = 3/group.

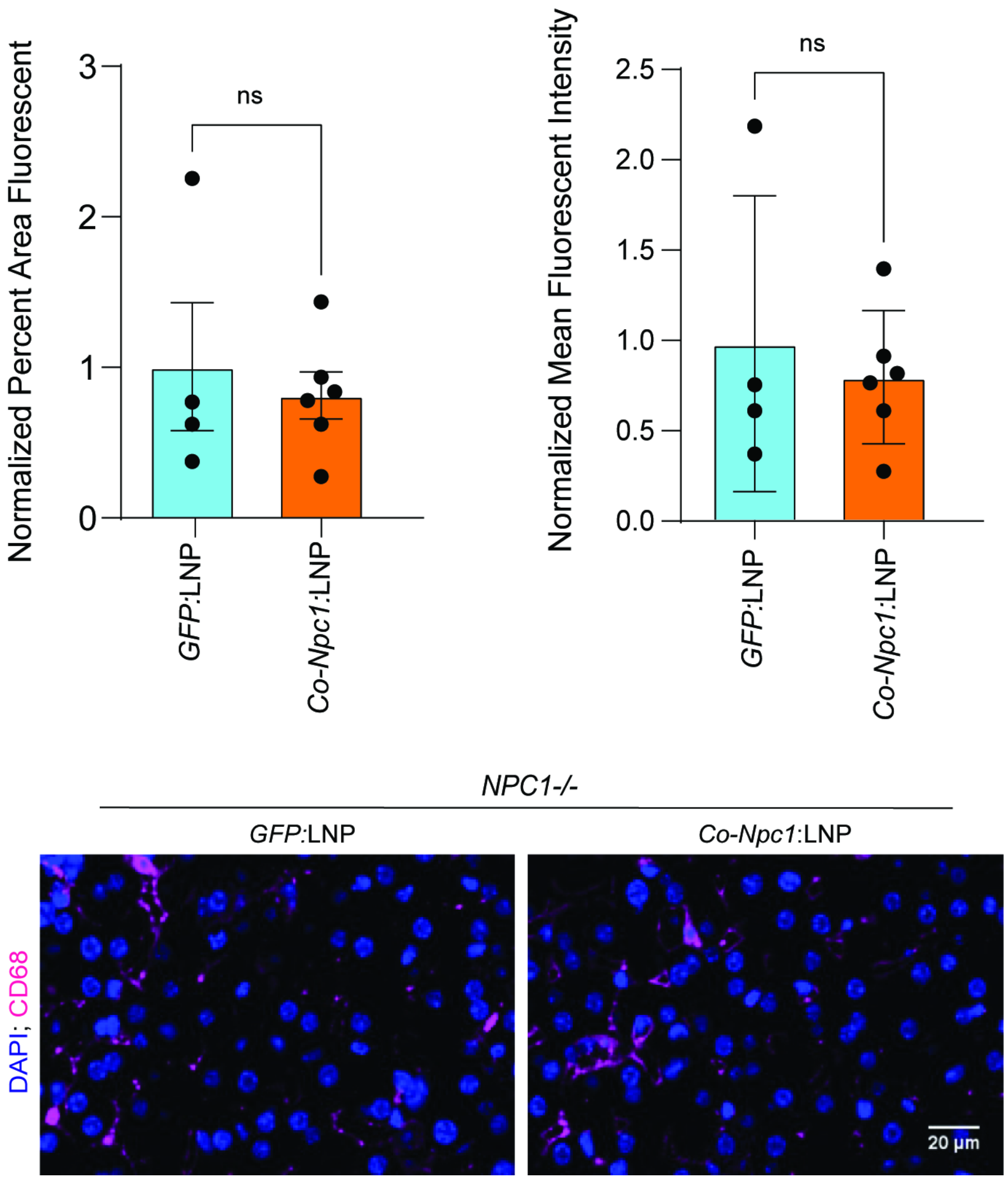

Figure S4. Macrophage populations in the mouse liver. *Npc1*$^{-/-}$ mice were injected intravenously with 1 mg/kg of *GFP*:LNP or *Co-Npc1:*LNP. 48 hours after injeciton, livers were perfused, collected, and stained with the macrophage marker CD68 (pink) and DAPI (blue). ns, not significant by unpaired t test: (t=0.4927, df=8), (t=0.4927, df=8), from left to right. Data are shown as the mean ± SEM from n = 4-6/group.

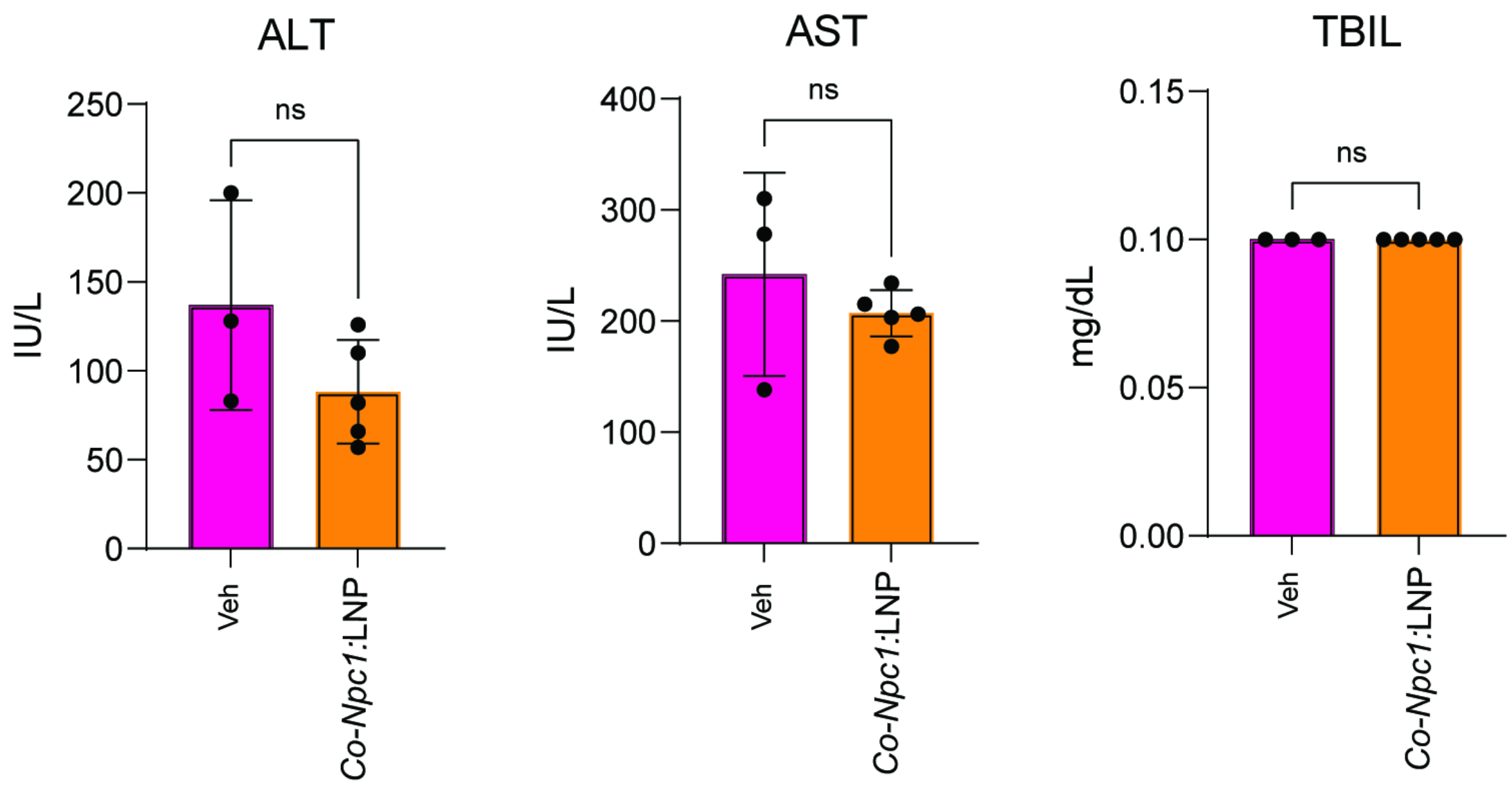

Figure S5. Liver enzyme tests 4 days after *Co-Npc1*:LNP treatment in *Npc1*$^{-/-}$ mice. 6-week-old *Npc1*$^{-/-}$ mice were injected once intravenously with 200 µL saline (Veh) or 1 mg/kg *Co-Npc1*:LNP. After 4 days, livers were harvested and analyzed for levels of the enzymes alanine aminotransferase (ALT), aspartate aminotransferase (AST), and total bilirubin (TBIL). ns, not significant by unpaired t test: (t=1.607, df=6), (t=0.8644, df=6), cannot conduct test due to identical values, from left to right. All values fell within the normal range for mouse liver enzyme function values. Data are shown as the mean ± SEM from n = 3-5/group.

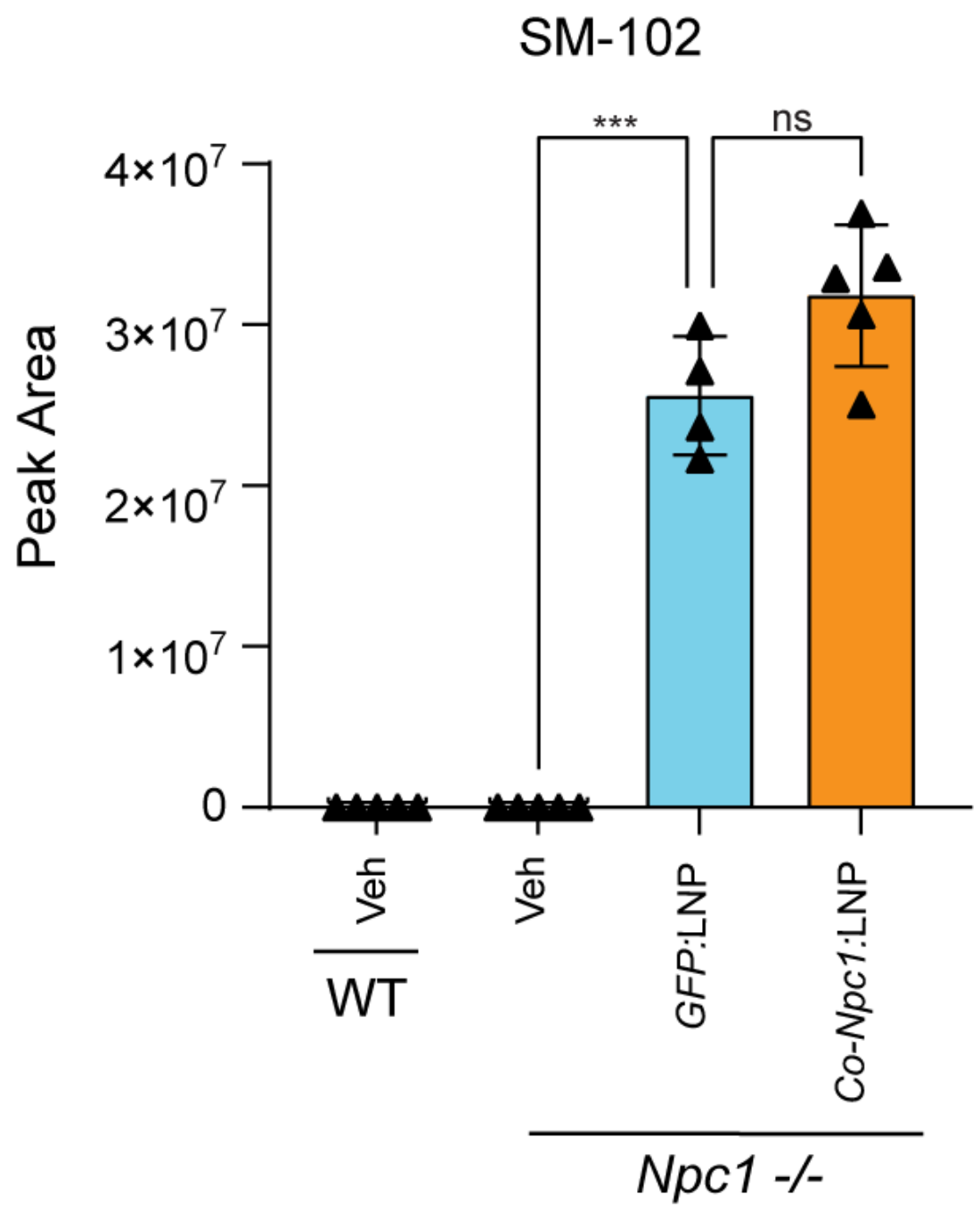

Figure S6. 3-week-old *Npc1*$^{+/+}$ (WT) and *Npc1*$^{-/-}$ mice treated with saline (Veh), 1 mg/kg *GFP*:LNP, or 1 mg/kg codon-optimized *Co-Npc1*:LNP. Livers were collected 48 hours after treatment. Levels of the synthetic lipid SM-102 were analyzed by liquid-chromatography-mass spectrometry. ns, not significant, ***$p \leq .001$ by one-way ANOVA with Tukey's multiple comparisons test; $F(3, 15) = 172$. Data are shown as the mean ± SEM from n = 3-5/group. Data points plotted as 0 represent samples in which SM-102 was below the limit of detection.

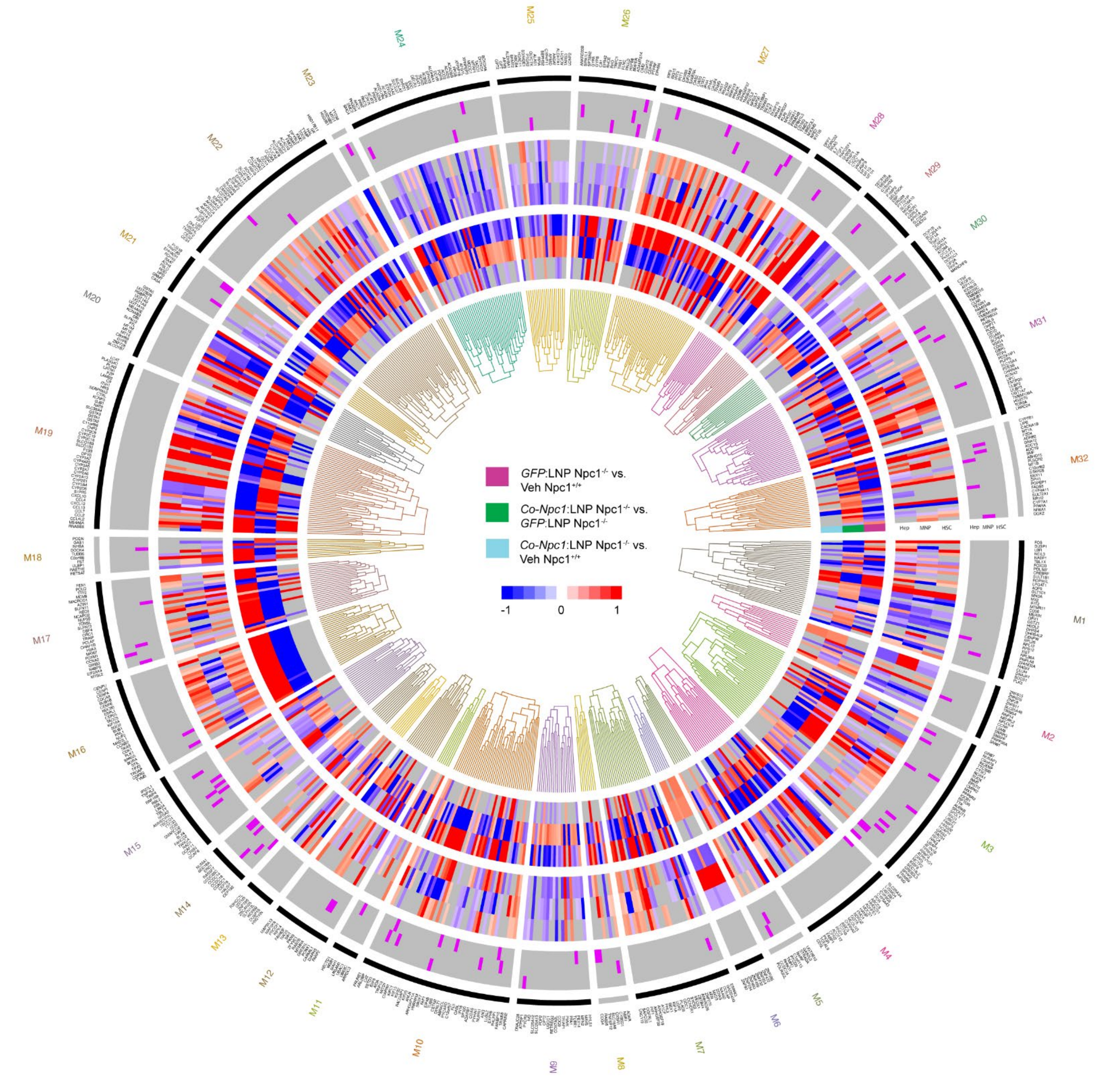

Figure S7. Integration of bulk RNA-seq data and snRNA-seq data reveals transcriptional correction across liver cell type populations following *Co-Npc1*:LNP treatment. Polar dendrogram visualization of MENTOR-derived gene modules integrating bulk RNA-seq and single-nucleus RNA-seq data. Genes are organized based on hierarchical clustering of RWR-derived dissimilarities across multiplex network, with modules (M1-M32) annotated along the outer ring. Heatmap tracks follow the module labels. The innermost heatmap track displays gene-level $\log_2$(FC) values for the bulk RNA-seq data. For the innermost track and the following tracks, the innermost column of displays *GFP:*LNP *Npc1*$^{-/-}$ vs. Vehicle *Npc1*$^{+/+}$*,* representing disease-associated transcriptional changes. The middle column shows *Co-Npc1*:LNP *Npc1*$^{-/-}$ vs. *GFP*:LNP *Npc1*$^{-/-}$, capturing treatment-induced transcriptional shifts. The last column represents *Co-Npc1*:LNP *Npc1*$^{-/-}$ vs. Vehicle *Npc1*$^{+/+}$, where non-differentially expressed genes (indicated in grey) show correction toward wild-type expression levels. Color scale ranges from blue (downregulation) to red (upregulation). The next heatmap (middle) track incorporates snRNA-seq results for hepatocytes (Hep), mononuclear phagocytes (MNP), and hepatic stellate cells (HSCs), displaying

the log_2(FC) values for each cell type. The next heatmap (outer) track indicates genes that are uniquely differentially expressed within a specific cell type (Hep, HSC, or MNP) and that also exhibit correction in the bulk RNA-seq data. The outermost track denotes module statistical significance, with black segments denoting modules that are significant relative to permutation-derived null distributions.

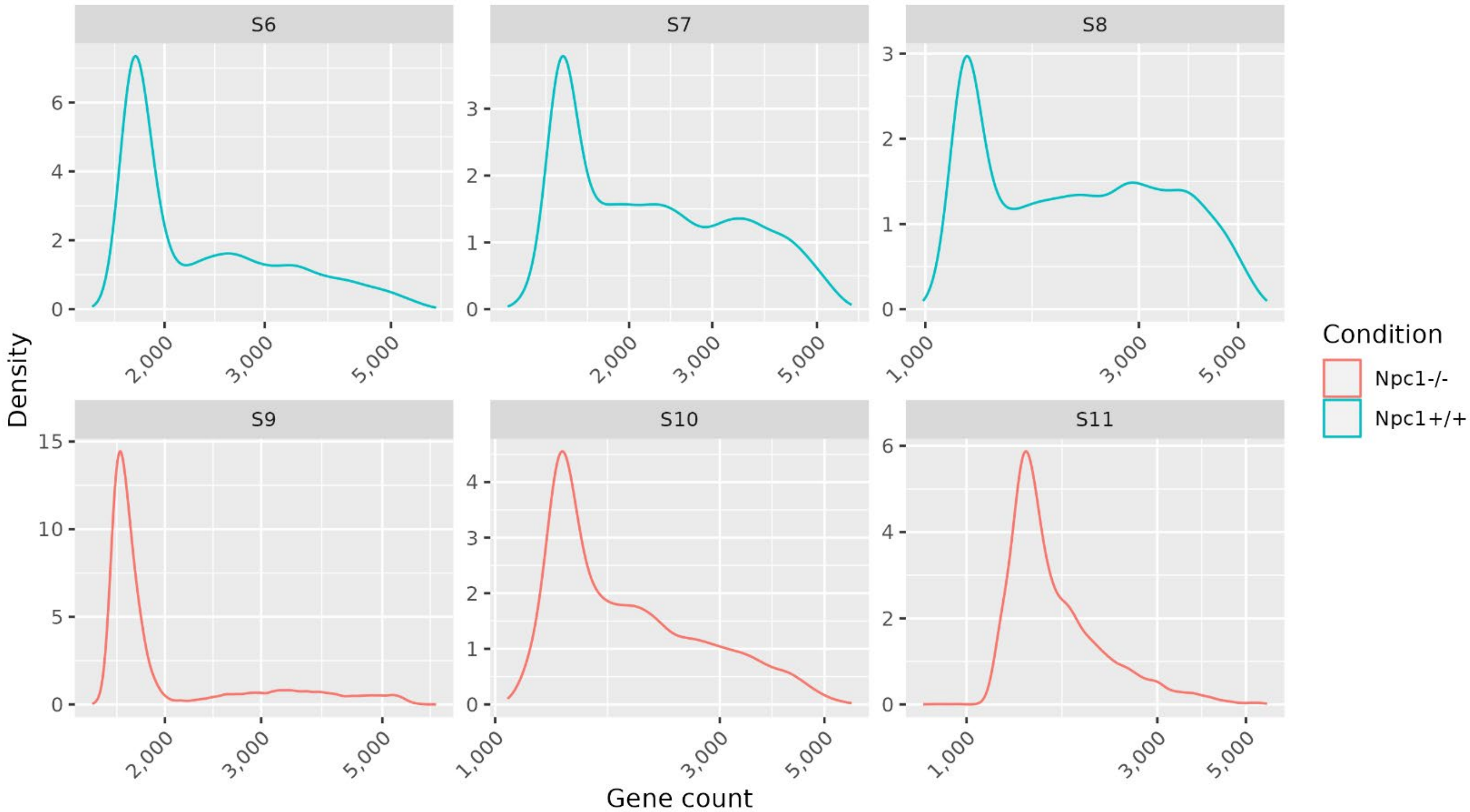

Figure S8. Single-nucleus RNA-sequencing data filtering. snRNA-seq was performed on liver samples from 7-week-old *Npc1*$^{+/+}$ and *Npc1*$^{-/-}$ mice. Distributions display unique gene count after quality control filtering per sample. n = 3/group.

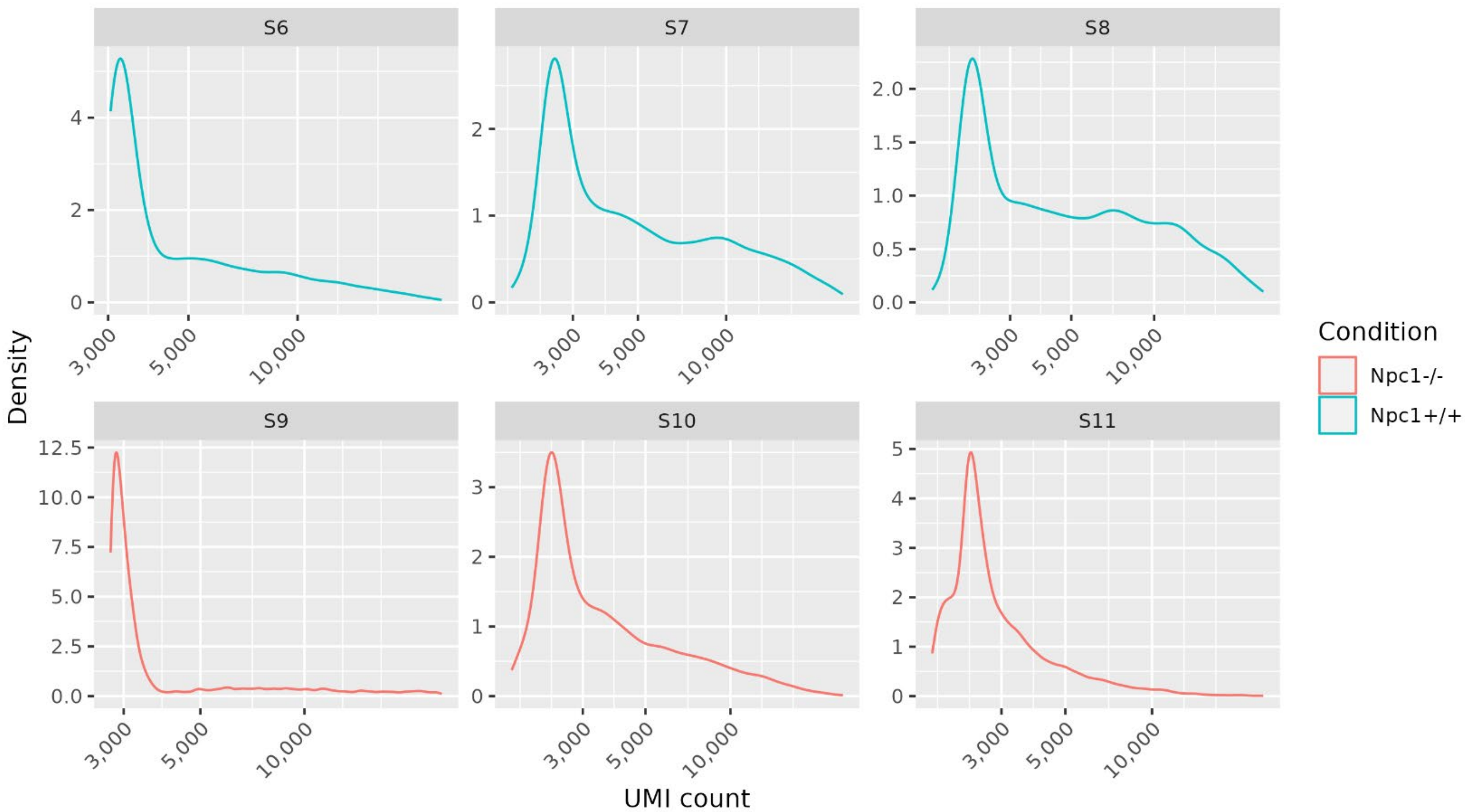

Figure S9. Single-nucleus RNA-sequencing data filtering. snRNA-seq was performed on liver samples from 7-week-old *Npc1*$^{+/+}$ and *Npc1*$^{-/-}$ mice. Distributions display total UMI after quality control filtering per sample. n=3/group.

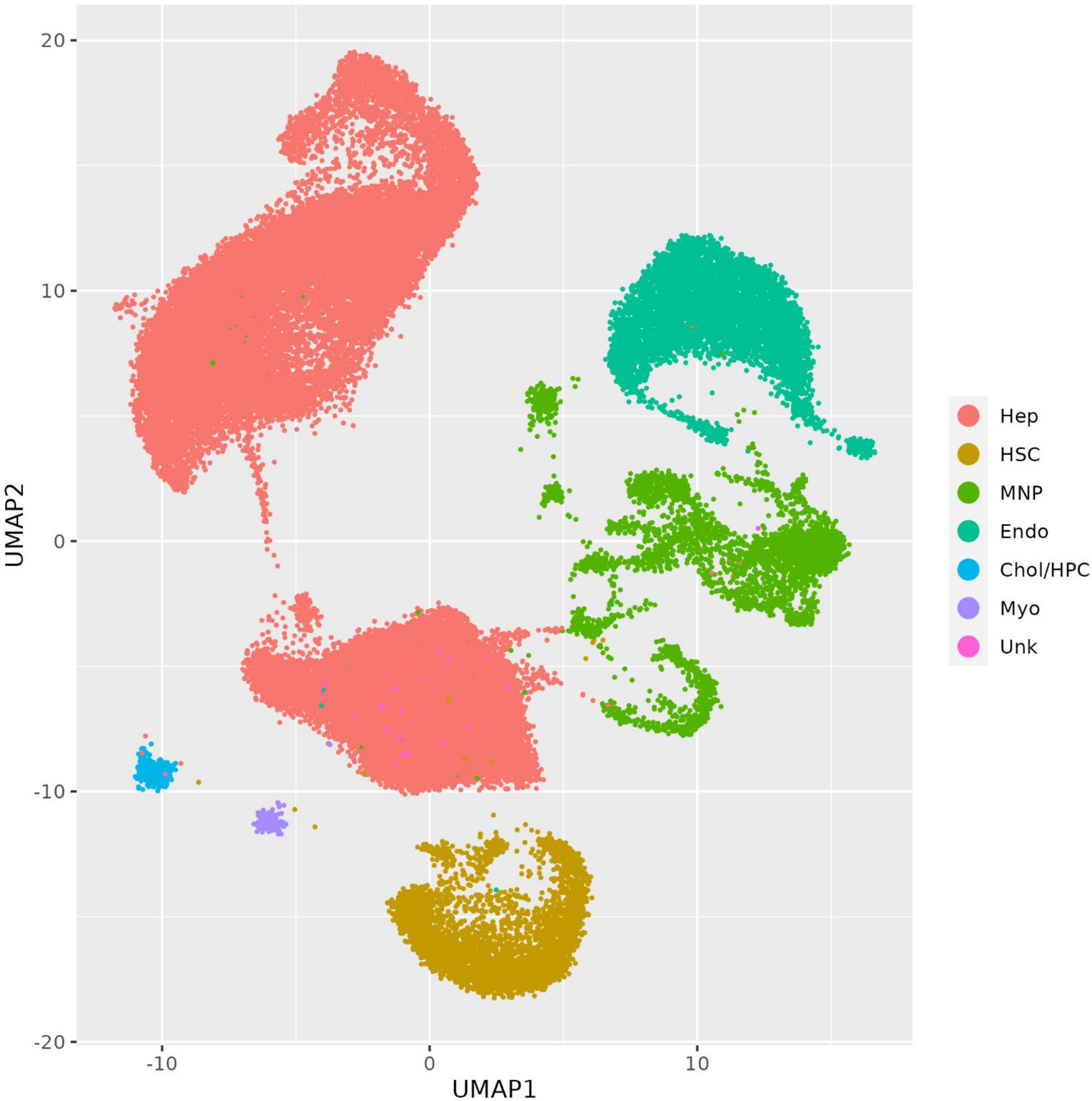

Figure S10. UMAP embeddings displaying results of Seurat dimensionality reduction, clustering, and differential expression analysis for manual annotation of major cell type populations. Cell types identified include hepatocytes (Hep), hepatic stellate cells (HSCs), mononuclear phagocytes (MNP), endothelial cells (Endo), cholinergic/hepatic progenitor cells (Chol/HPC), myofibroblasts (Myo), and a small population of unknown cells (Unk). Cell type clusters are preserved across genotypes.

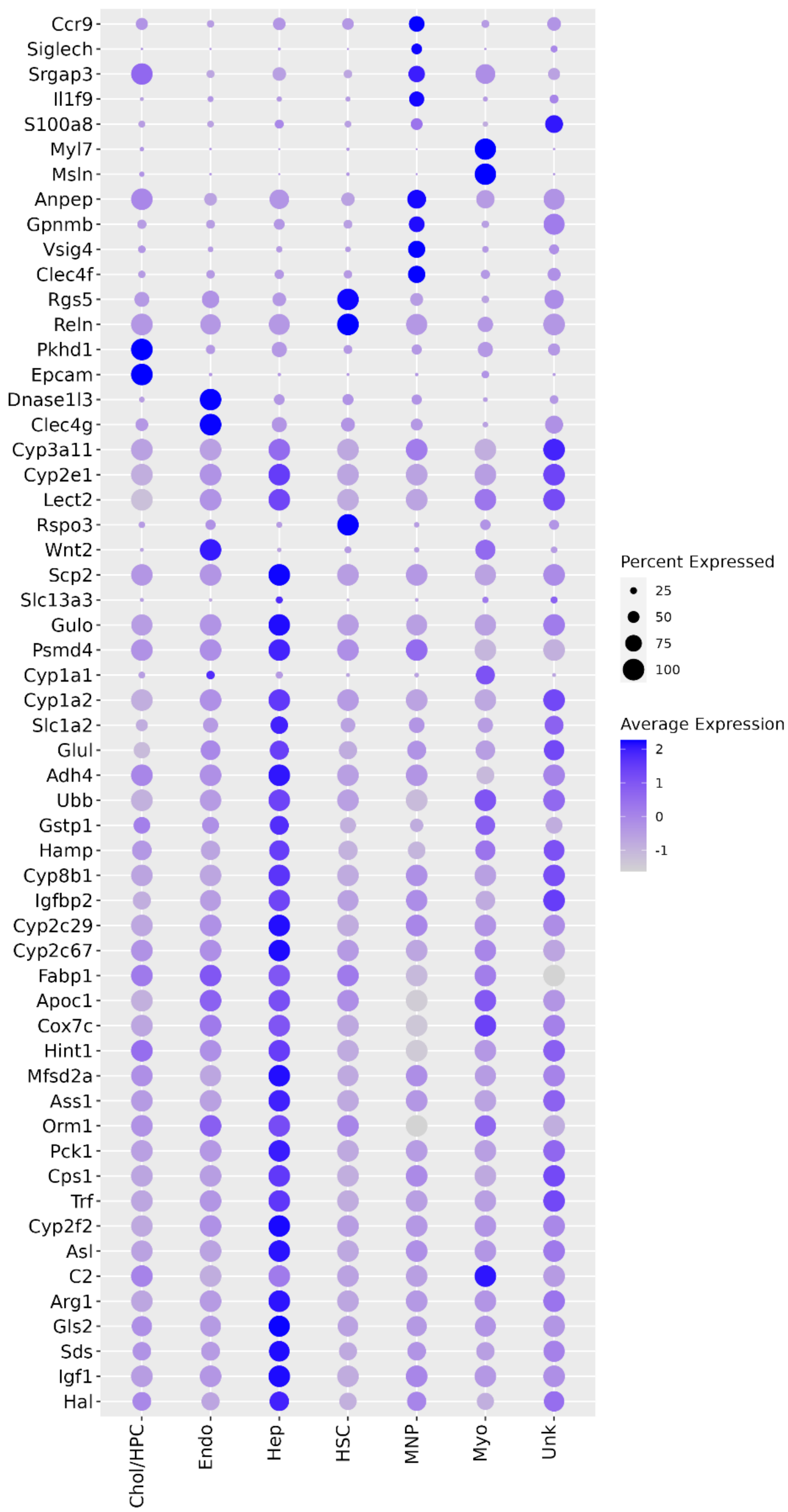

Figure S11. Dot plot visualization of cell type-specific marker gene expression across major cell type populations identified from snRNA-seq data. Rows correspond to genes and columns correspond to corresponding cell types, including hepatocytes (Hep), hepatic stellate cells (HSCs), mononuclear phagocytes (MNP), endothelial cells (Endo), cholinergic/hepatic progenitor cells (Chol/HPC), myofibroblasts (Myo), and a small population of unknown cells (Unk). Size of points represent percentages of cells within each cluster expressing the marker gene and color intensity represents scaled gene expression, ranging from low (light purple) to high (blue).

```
1 ATGGGAGCTC ATCACCCAGC TCTGGGACTT CTTTTGCTTC TGCTCTGTCC AGCACAAGTT
61 TTTTCACAAT CATGTGTATG GTACGGGGAG TGTGGAATAG CAACCGGCGA TAAACGCTAT
121 AATTGTAAAT ATTCAGGACC CCCAAAGCCC CTGCCAAAGG ACGGATATGA TTTGGTGCAG
181 GAGCTCTGTC CTGGCTTGTT TTTTGATAAC GTGTCACTGT GTTGTGACAT ACAACAACTC
241 CAGACTCTGA AGAGCAATCT TCAATTGCCC CTCCAGTTTC TCTCTAGGTG TCCATCTTGC
301 TTTTACAATC TTATGACCCT TTTTTGCGAG TTGACTTGTT CACCACATCA GAGTCAATTC
361 TTGAACGTCA CCGCCACCGA GGATTACTTT GACCCTAAGA CACAAGAAAA TAAGACTAAT
421 GTGAAGGAGT TGGAATACTT CGTTGGGCAG TCATTCGCAA ACGCAATGTA CAATGCTTGC
481 CGAGACGTGG AAGCACCCTC CAGCAACGAA AAAGCCCTTG GCCTTCTGTG TGGGCGGGAT
541 GCTCGCGCAT GCAATGCCAC AAACTGGATC GAATACATGT TCAACAAGGA CAATGGTCAG
601 GCCCCTTTTA CTATCATACC CGTATTCTCT GATTTGTCCA TATTGGGCAT GGAACCAATG
661 AGAAACGCTA CTAAGGGGTG CAATGAATCA GTGGATGAAG TGACTGGACC TTGCAGCTGT
721 CAAGATTGTT CCATTGTCTG TGGTCCAAAA CCACAGCCAC CTCCTCCACC AATGCCATGG
781 CGCATATGGG GACTCGATGC AATGTACGTC ATCATGTGGG TCACTTATGT CGCCTTCCTT
841 TTTGTTTTTT TTGGCGCACT TTTGGCAGTA TGGTGCCATC GGCGACGATA TTTCGTATCT
901 GAGTATACTC CCATAGATTC TAATATCGCT TTCAGTGTTA ATTCAAGCGA CAAAGGAGAA
961 GCCTCCTGCT GCGATCCTCT CGGAGCTGCC TTTGACGACT GTCTCAGACG CATGTTCACC
1021 AAGTGGGGAG CCTTTTGTGT CCGCAATCCA ACTTGTATCA TCTTTTTCTC CTTGGCATTT
1081 ATTACCGTGT GCAGTTCCGG ACTGGTTTTT GTCCAAGTTA CTACAAATCC AGTGGAACTG
1141 TGGAGTGCAC CCCACAGCCA AGCCAGACTG GAAAAGGAAT ACTTTGACAA GCACTTTGGA
1201 CCTTTCTTCC GCACAGAACA GCTGATAATC CAGGCCCCAA ATACCAGTGT GCATATATAT
1261 GAGCCTTATC CCGCCGGCGC CGACGTGCCT TTCGGTCCCC CTCTTAACAA AGAGATCCTC
1321 CATCAAGTCC TGGATCTCCA GATAGCCATC GAAAGTATCA CTGCAAGCTA CAACAATGAG
1381 ACTGTTACCT TGCAAGATAT ATGTGTAGCC CCACTGTCTC CCTATAATAA AAATTGTACA
1441 ATAATGTCCG TGCTCAATTA CTTTCAAAAT AGTCACGCTG TTTTGGACAG TCAAGTCGGC
1501 GATGATTTCT ACATCTATGC CGATTACCAC ACCCATTTTC TGTATTGCGT CCGAGCCCCT
1561 GCCAGCCTGA ACGACACAAG TCTTCTTCAC GGCCCATGTT TGGGTACCTT CGGAGGTCCC
1621 GTCTTTCCCT GGCTCGTGCT TGGAGGCTAC GACGATCAAA ATTACAATAA TGCCACAGCT
1681 CTGGTCATAA CTTTTCCTGT GAATAACTAC TACAACGACA CTGAAAGGTT GCAACGCGCC
1741 TGGGCCTGGG AAAAGGAGTT CATTAGTTTC GTTAAGAATT ACAAGAATCC AAATTTGACC
1801 ATCTCATTCA CTGCAGAACG GTCAATCGAA GATGAACTCA ACCGCGAAAG TAACTCTGAC
1861 GTATTCACCG TTATAATCTC CTATGTTGTG ATGTTTCTGT ACATCTCACT TGCATTGGGA
1921 CATATTCAAT CATGTTCACG GCTCCTCGTC GACTCTAAGA TCAGCTTGGG GATAGCCGGG
1981 ATTTTGATCG TACTGTCAAG TGTGGCCTGT AGCCTTGGGA TCTTTTCTTA TATGGGTATG
2041 CCACTGACAC TGATAGTGAT AGAGGTGATA CCTTTCTTGG TATTGGCCGT GGGGGTAGAT
2101 AACATTTTCA TTCTGGTCCA AACCTACCAA CGCGACGAAA GGCTGCAAGA GGAGACCCTG
2161 GACCAACAAC TCGGGAGAAT CCTTGGAGAG GTCGCCCCCA CAATGTTCCT TTCATCATTC
2221 TCCGAAACAT CTGCATTTTT CTTCGGTGCC CTTAGCTCCA TGCCCGCTGT TCATACTTTC
2281 AGCCTGTTTG CAGGAATGGC AGTCCTCATA GATTTTCTGT TGCAAATTAC CTGCTTCGTA
2341 TCATTGCTTG GCTTGGACAT AAAACGACAG GAAAAAAATC ATCTCGACAT ATTGTGCTGT
2401 GTACGCGGCG CCGACGACGG CCAAGGCTCA CACGCTTCTG AATCCTACCT GTTTAGGTTC
2461 TTCAAGAATT ACTTCGCACC CCTGCTCTTG AAAGACTGGC TTAGACCTAT TGTAGTAGCC
2521 GTGTTCGTCG GTGTACTGTC TTTCAGTGTA GCCGTCGTGA ATAAGGTCGA CATAGGGTTG
2581 GATCAATCCT TGTCCATGCC TAATGATAGC TATGTCATAG ACTACTTCAA GAGTCTGGCC
2641 CAGTACTTGC ATTCCGGACC TCCTGTTTAT TTTGTACTGG AGGAAGGATA TAATTATTCA
2701 AGTCGCAAGG GACAGAATAT GGTGTGTGGT GGAATGGGGT GCGACAACGA CTCACTGGTA
2761 CAACAGATCT TTAACGCCGC CGAATTGGAT ACATACACTA GGGTCGGGTT CGCCCCCTCT
2821 TCATGGATTG ATGACTACTT CGATTGGGTG AGCCCTCAAT CAAGCTGTTG TAGGCTCTAT
2881 AATGTAACAC ACCAGTTCTG TAACGCCTCC GTCATGGACC CAACTTGTGT TAGATGTCGC
2941 CCACTCACAC CCGAAGGAAA ACAAAGACCA CAGGGGAAAG AATTTATGAA GTTCCTCCCT
3001 ATGTTTTTGA GTGATAACCC AAATCCTAAG TGCGGAAAAG GGGGCCACGC TGCCTATGGC
3061 TCTGCAGTCA ACATCGTTGG CGATGATACA TACATTGGTG CCACTTACTT TATGACATAC
3121 CATACAATAC TTAAAACATC AGCCGACTAT ACTGACGCTA TGAAAAAGGC TAGGCTCATC
3181 GCATCAAACA TTACTGAAAC TATGCGCAGT AAGGGGAGTG ACTATCGAGT ATTCCCATAT
3241 AGTGTATTCT ACGTATTCTA TGAGCAATAT CTTACTATCA TCGATGATAC AATCTTTAAT
3301 CTTTCCGTTA GTCTGGGTTC TATTTTTTTG GTGACTCTCG TAGTTCTTGG GTGTGAATTG
3361 TGGTCCGCCG TAATAATGTG TATTACAATA GCTATGATTC TTGTTAATAT GTTTGGCGTG
3421 ATGTGGTTGT GGGGGATCTC CCTCAATGCT GTGTCATTGG TGAACTTGGT GATGAGCTGT
3481 GGAATCAGTG TAGAGTTCTG CAGTCACATC ACTAGAGCTT TTACCATGAG TACCAAGGGG
3541 TCTAGGGTTA GCCGAGCTGA GGAGGCTCTG GCCCACATGG GGAGTTCTGT ATTTTCTGGA
3601 ATTACACTGA CAAAATTTGG AGGCATCGTA GTGCTGGCAT TCGCCAAGTC TCAAATCTTT
3661 GAGATCTTCT ACTTTCGGAT GTATCTTGCT ATGGTATTGC TTGGCGCAAC CCACGGCCTT
3721 ATCTTCCTGC CCGTACTCTT GAGCTACATT GGTCCTAGTG TTAACAAGGC CAAGCGACAC
3781 ACCACATACG AACGGTACAG GGGTACCGAG AGAGAGCGGC TGCTTAATTT CTGA
```

Figure S12. mRNA sequence for codon-optimized *Npc1.* Base differences between codon-optimized and endogenous *Npc1* are delineated by bold and elevated text

Supplemental Tables

Table S1. Particle characterization of *GFP*:LNP, *Npc1*:LNP, and *Co-Npc1*:LNP formulations. Particle Size, polydispersity Index (PDI), and encapsulation efficiency (EE) are shown.

**Formulation**	**Size (nm)**	**PDI**	**EE**
GFP:LNP	84	0.097	99%
Npc1:LNP	100.9	0.127	98%
Co-Npc1:LNP	94.35	0.126	96%

Table S2. MENTOR-IA themes describing each module within the MENTOR dendrogram. Found in supplemental spreadsheet file.

Table S3. Marker genes used for cell type clustering of snRNA-seq data.

Marker gene symbol	Liver cell type
Hal	Hepatocytes (periportal)
Igf1	Hepatocytes (periportal)
Sds	Hepatocytes (periportal)
Gls2	Hepatocytes (periportal)
Arg1	Hepatocytes (periportal)
C2	Hepatocytes (periportal)
Asl	Hepatocytes (periportal)
Cyp2f2	Hepatocytes (periportal)
Trf	Hepatocytes (periportal)
Cps1	Hepatocytes (periportal)
Pck1	Hepatocytes (periportal)
Orm1	Hepatocytes (periportal)
Ass1	Hepatocytes (periportal)
Mfsd2a	Hepatocytes (periportal)
Hint1	Hepatocytes (midzonal)
Cox7c	Hepatocytes (midzonal)
Apoc1	Hepatocytes (midzonal)
Fabp1	Hepatocytes (midzonal)
Cyp2c67	Hepatocytes (midzonal)
Cyp2c29	Hepatocytes (midzonal)
Igfbp2	Hepatocytes (midzonal)
Cyp8b1	Hepatocytes (midzonal)
Hamp	Hepatocytes (midzonal)
Gstp1	Hepatocytes (midzonal)
Ubb	Hepatocytes (midzonal)
Adh4	Hepatocytes (pericentral)
Glul	Hepatocytes (pericentral)
Slc1a2	Hepatocytes (pericentral)
Cyp1a2	Hepatocytes (pericentral)
Cyp1a1	Hepatocytes (pericentral)
Psmd4	Hepatocytes (pericentral)
Gulo	Hepatocytes (pericentral)
Slc13a3	Hepatocytes (pericentral)
Scp2	Hepatocytes (pericentral)
Wnt2	Hepatocytes (pericentral)
Rspo3	Hepatocytes (pericentral)
Lect2	Hepatocytes (pericentral)
Cyp2e1	Hepatocytes (pericentral)
Cyp2c29	Hepatocytes (pericentral)
Cyp3a11	Hepatocytes (pericentral)
Clec4g	Endothelial cells
Dnase1l3	Endothelial cells

Epcam	Cholangiocyte/hepatic progenitor cells
Pkhd1	Cholangiocyte/hepatic progenitor cells
Reln	Hepatic stellate cells
Rgs5	Hepatic stellate cells
Clec4f	Kupffer cells
Vsig4	Kupffer cells
Gpnmb	Macrophages
Anpep	Macrophages
Msln	Myofibroblasts
Myl7	Myofibroblasts
S100a8	Neutrophils
Il1f9	Neutrophils
Srgap3	Plasmacytoid dendritic cells
Siglech	Plasmacytoid dendritic cells
Ccr9	Plasmacytoid dendritic cells

Table S4. Differential cell-type abundance in livers from *Npc1*$^{+/+}$ and *Npc1*$^{-/-}$ mice. Average proportion of each cell type across all samples (BaselineProp.Freq), mean proportion of cell type in *Npc1*$^{-/-}$ samples (PropMean.KO), mean proportion of cell type in *Npc1*$^{+/+}$ samples (PropMean.WT), *Npc1*$^{-/-}$ proportion divided by *Npc1*$^{+/+}$ proportion (PropRatio), test statistic (Tstatistic), unadjusted p-value (P.Value), and false-discovery rate-adjusted p-value.

Cell Type	BaselineProp.Freq	PropMean.KO	PropMean.WT	PropRatio	Tstatistic	P.Value	FDR
MNP	0.0681	0.098	0.0395	2.4795	3.0395	0.0056	0.0339
Myo	0.0029	0.0018	0.0039	0.4799	-2.1346	0.0432	0.1296
Endo	0.0901	0.0677	0.1112	0.6084	-1.8502	0.0766	0.1533
HSC	0.0625	0.0494	0.0747	0.662	-1.5076	0.1447	0.217
Chol/HPC	0.0058	0.0052	0.0063	0.8217	-0.8721	0.3918	0.4701
Hep	0.7707	0.7779	0.7644	1.0176	0.3211	0.7509	0.7509

Table S5. Materials and reagents**.** Reagents used for described experiments, type of reagent, dilution used when applicable, and vendor with product number when applicable.

Reagent/Kit	Type	Dilution	Vendor
NPC1	Primary antibody	1:500	Abcam (ab134113/clone EPR5209)
GFAP	Primary antibody	1:1000	Abcam (AB290)
Actin	Primary antibody	1:2000	Millipore (Sigma A5441/clone AC-15)
LC3B	Primary antibody	1:500	Cell Signaling (3868S/(D11))
Vinculin	Primary antibody	1:2000	Sigma (V9131/clone hVIN-1)
CD68	Primary antibody	1:250	Santa Cruz Biotechnology (sc-20060)
Goat anti-mouse IgG (H+L)-HRP conjugate	Secondary antibody	1:2000	Bio-Rad (170-6516)
Goat anti-rabbit IgG (H+L)-HRP conjugate	Secondary antibody	1:2000	Bio-Rad (170-6515)
Wheat germ agglutinin 488	Stain	1:1000	Invitrogen (W11261)
Filipin	Stain	1µg	Sigma (SAE0087-1ML)
D-Luciferin, Potassium Salt	synthetic luciferin	NA	Invitrogen (L2916)
SM-102 (MW 710.2) (33474)	ionizable cationic lipid	NA	Cayman Chemical
Cholesterol (MW 386.65) (C8667)	Sterol	NA	Sigma Aldrich
1,2-distearoyl-sn-glycero-3-phosphocholine (DSPC, 18:0 PC; MW 790.2) (850365P)	Phospholipid	NA	Avanti Polar Lipids
1,2-dimyristoyl-rac-glycero-3-methoxypolyethylene glycol-2000 (DMG-PEG2000; MW 2526) (880151P)	pegylated lipid	NA	Avanti Polar Lipids
Quant-iT™ RiboGreen™ RNA Assay Kit (R11490)	Kit	NA	ThermoFisher Scientific
CleanCap® *EGFP* mRNA (L-7601)	*eGFP* mRNA	NA	TriLink Biotechnologies
CleanCap® *Npc1* mRNA (L-7601)	*Npc1* mRNA	NA	TriLink Biotechnologies
Npc1 mRNA qPCR probe	qPCR probe	NA	Integrated DNA technologies
Codon-optimized *Npc1* mRNA qPCR probe	taqMan Assay qPCR probe<br><br>5' to 3' for all primers and probes:<br><br>Forward Primer GCATTCGCCAAGTCTCAAATC	NA	Integrated DNA technologies

	Reverse Primer:<br>AGAGTACGGGCAGGAAGATA<br><br>Probe:<br>GCTATGGTATTGCTTGGCGCAA		

Table S6. Multiple Reaction Monitoring transitions and optimized collision energies for LC-MS analysis of cholesterol and cholesteryl esters

Compound name	Precursor m/z	Product m/z	Transition type	RT (min)	CE (V)	iFunnel mode
CE 16:0	642.6	369.3	Quantifier	10.2	10	Fragile
CE 16:0	642.6	161.1	Qualifier	10.2	43	Fragile
CE 18:0	670.6	369.3	Quantifier	12	10	Fragile
CE 18:0	670.6	161.1	Qualifier	12	43	Fragile
CE 18:2	666.6	369.3	Qualifier	9.2	10	Fragile
CE 18:2	666.6	161.1	Quantifier	9.2	43	Fragile
CE 20:4	690.6	369.3	Qualifier	8.6	10	Fragile
CE 20:4	690.6	161.1	Quantifier	8.6	43	Fragile
CE 22:6	714.6	369.3	Quantifier	8	10	Fragile
CE 22:6	714.6	161.1	Qualifier	8	43	Fragile
CE 16:0-d7	649.6	376.4	Quantifier	10.2	10	Fragile
CE 16:0-d7	649.6	147.2	Qualifier	10.2	45	Fragile
Cholesterol	369.3	161.1	Qualifier	4.1	18	Standard
Cholesterol	369.3	81	Quantifier	4.1	45	Standard
Cholesterol-d7	376.3	161.1	Qualifier	4.1	22	Standard
Cholesterol-d7	376.3	81.1	Quantifier	4.1	45	Standard

Supplemental Methods

Transmission Electron Microscopy

The morphology of LNPs was observed using transmission electron microscopy (TEM) with negative staining. LNP samples were concentrated to a final RNA concentration of 0.25 mg/mL using 10 kDa MWCO Amicon Ultra filter units. The concentrated samples were then prepared by applying 4 µL of LNPs to a glow-discharged 400-mesh copper grid with formvar carbon film (Electron Microscopy Sciences) for 1 minute. The grids were washed and negative stained with 0.1% uranyl acetate. The samples were imaged using a 100kV FEI Morgagni electron microscope equipped with a Gatan Orius SC200 CCD detector at a magnification of 22,000X (2.1 Å/pixel).

Small Animal Imaging

For *in vivo* live imaging, mice were injected with 200 µL saline or 1 mg/kg LNP. After 24 hours, mice were injected with 15 mg/kg d-luciferin and imaged for luminescent signal 20 minutes after the d-luciferin injection. Imaging was done using the IVIS® Lumina™ S5 high-throughput 2-D optical imaging system from the Small Animal Imaging Core at the University of Iowa. Radiance values were obtained from ROIs encompassing each mouse on medium-binning and compared between treatment groups.

Immunohistochemistry

Mice were injected via tail vein with 1 mg/kg LNP and livers were collected after 48 hours. Tissue was sectioned and stained using a CD68 antibody and DAPI nuclear stain by the University of Iowa Histology Research Laboratory and Comparative Pathology Laboratory.
