# Supplementary material for: Codon-optimized Npc1 mRNA corrects Niemann-Pick type C1 disease phenotypes in vitro and in vivo
Source: Mol Ther Nucleic Acids. 2026 Jul 18;37(3):103022. doi: 10.1016/j.omtn.2026.103022 (PMC13521171; doi:10.1016/j.omtn.2026.103022)
Supplement: Supplementary file 2 [file mmc3.pdf]

Molecular Therapy
Nucleic Acids
Original Article

Codon-optimized *Npc1* mRNA corrects Niemann-Pick type C1 disease phenotypes *in vitro* and *in vivo*

Ellen R. Koufer,[1,2,10] Alice Townsend,[3,10] Youngseo Na,[4,10] Adele B. Correia,[5,10] Noah K. Latham,[1,10] Koralege C. Pathmasiri,[6] Olga Ilnytska,[7] Judith Storch,[7] Kyle A. Sullivan,[8] Troy Halseth,[4] Thaddeus J. Kunkel,[5] Elizabeth S. Phillippi,[1] Matthew L. Lieberman,[1] Stephanie M. Cologna,[6,9] Andrew P. Lieberman,[5] Daniel A. Jacobson,[8] Anna S. Schwendeman,[4] and Mark L. Schultz[1]

[1]Stead Family Department of Pediatrics, University of Iowa, Iowa City, IA 52242, USA; [2]Interdisciplinary Graduate Program in Genetics, University of Iowa, Iowa City, IA 52242, USA; [3]Bredesen Center for Interdisciplinary Research and Graduate Education, University of Tennessee-Knoxville, Knoxville, TN 37996, USA; [4]Department of Pharmaceutical Sciences, University of Michigan, Ann Arbor, MI 48109, USA; [5]Department of Pathology, University of Michigan, Ann Arbor, MI 48109, USA; [6]Department of Chemistry, College of Liberal Arts and Sciences, University of Illinois, Chicago, Chicago, IL 60607-7061, USA; [7]Department of Nutritional Sciences, Rutgers University, New Brunswick, NJ 08901, USA; [8]Computational and Predictive Biology, Oak Ridge National Laboratory, Oak Ridge, TN 37831, USA; [9]Laboratory for Integrative Neuroscience, University of Illinois at Chicago, Chicago, IL 60607, USA

Niemann-Pick type C1 disease is a lysosomal storage disorder caused by mutations in the *NPC1* gene, resulting in the accumulation of unesterified cholesterol in multiple tissues. Despite its severity, therapeutic options remain limited. Using codon-optimized *Npc1* mRNA delivered by lipid nanoparticles (*Co-Npc1*:LNPs), we achieved enhanced NPC1 protein expression and prolonged therapeutic activity *in vitro*, correcting both primary and secondary disease defects. In an *Npc1*$^{-/-}$ mouse model, a single intravenous injection of *Co-Npc1*:LNP restored hepatic NPC1 protein levels, normalized autophagic flux, improved lipid abnormalities, and altered markers of liver injury. To interpret transcriptional changes post-*Co-Npc1*:LNP administration, we performed bulk RNA sequencing and applied MENTOR, a network-based clustering algorithm, and MENTOR-IA, an unbiased functional annotation tool. We identified transcriptionally restored pathways including cholesterol metabolism, lysosomal and mitochondrial function, and liver homeostasis. Overall, the *Npc1*$^{-/-}$ mouse liver showed a transcriptional shift toward a more *Npc1*$^{+/+}$ state post-*Co-Npc1*:LNP treatment. Integration of single-nucleus RNA sequencing with bulk transcriptomics further revealed cell-type-specific correction of disease-associated gene expression across disease-relevant hepatic cell types. Together, we report the development and *in vivo* validation of the first mRNA-based therapeutic for Niemann-Pick type C1 disease.

INTRODUCTION

Niemann-Pick type C1 disease is a fatal, autosomal recessive disorder characterized by the accumulation of unesterified cholesterol in neuronal and peripheral tissues.[1,2] Affected individuals exhibit a heterogeneous clinical presentation including progressive neurodegeneration and hepatic dysfunction.[1,3] Many children with Niemann-Pick type C1 develop hepatomegaly, with liver pathology being particularly severe in those with neonatal onset.[1] In this subgroup with hepatomegaly, up to 10% of individuals develop fatal liver failure before 6 months of age.[3] Hepatic manifestations are poorly described, but include steatosis, hepatocyte (Hep) ballooning, giant cell formation, and cirrhosis, which may progress into hepatocellular carcinoma.[4–6] Interestingly, hepatomegaly often improves during adolescence, suggesting that early therapeutic interventions targeting liver dysfunction may help affected children survive the critical early years until hepatic function stabilizes.[3]

Niemann-Pick type C1 is caused by over 300 loss-of-function point mutations in the *NPC1* gene, which encodes a multipass transmembrane glycoprotein required for exporting cholesterol from late endosomes and lysosomes.[7,8] Loss of NPC1 function causes the accumulation of cholesterol and severe downstream secondary defects including lysosomal dysfunction, autophagy impairment, and mitochondrial defects.[9] These secondary phenotypes are complex,

Received 19 November 2025; accepted 16 July 2026;
https://doi.org/10.1016/j.omtn.2026.103022.

[10]These authors contributed equally

Correspondence: Andrew P. Lieberman, Department of Pathology, University of Michigan, Ann Arbor, MI 48109, USA.
E-mail: liebermn@med.umich.edu
Correspondence: Daniel A. Jacobson, Computational and Predictive Biology, Oak Ridge National Laboratory, Oak Ridge, TN 37831, USA.
E-mail: jacobsonda@ornl.gov
Correspondence: Anna S. Schwendeman, Department of Pharmaceutical Sciences, University of Michigan, Ann Arbor, MI 48109, USA.
E-mail: annaschw@umich.edu
Correspondence: Mark L. Schultz, Stead Family Department of Pediatrics, University of Iowa, Iowa City, IA 52242, USA.
E-mail: mark-schultz-1@uiowa.edu

Published by Elsevier Inc. on behalf of The American Society of Gene and Cell Therapy.
This is an open access article under the CC BY license (http://creativecommons.org/licenses/by/4.0/).

interconnected, and difficult to target individually. Therefore, therapeutic strategies that directly target the primary defect in Niemann-Pick type C1 are critical.

The monogenic nature of Niemann-Pick type C1 and significant hepatic involvement make mRNA replacement strategies an appealing therapeutic avenue.[10] Recent advances in molecular biology and chemical synthesis have greatly reduced mRNA immunogenicity (e.g., substitution with N1-methylpseudouridine), increased synthesis efficiency, and accelerated the clinical translation of mRNA therapeutics.[11–15] However, mRNA is rapidly degraded by extracellular and intracellular RNases. This challenge has been overcome through mRNA encapsulation in lipid nanoparticles (LNPs), nanoscale carriers composed of biocompatible synthetic lipids that protect mRNA and enable cellular uptake and translation under native biological conditions.[16,17] LNPs containing mRNA have demonstrated efficient liver transfection in both preclinical and clinical settings.[18,19]

mRNA therapeutics have the potential to restore functional NPC1 protein expression, correct NPC1 dysfunction, and mitigate downstream pathology. Several challenges remain regarding the efficient production of large transmembrane proteins such as NPC1 from exogenous mRNA, and it is unclear how diseased cells globally respond to short-term expression of a corrected protein in the context of genetic disease. Nevertheless, these findings highlight the promise of mRNA-based therapies and provide a foundation for future studies aimed at developing durable treatments for Niemann-Pick type C1 disease.

Here, we leveraged codon-optimization to enhance translation while maintaining proper folding of the NPC1 protein, correcting both primary and secondary cellular defects in an *in vitro* model of Niemann-Pick type C1. To test the potential of mRNA therapy *in vivo*, we utilized an *Npc1*$^{-/-}$ mouse model, which mimics many of the liver and molecular phenotypes seen in individuals with Niemann-Pick type C1. A single intravenous (i.v.) injection of LNPs containing codon-optimized *Npc1*-corrected autophagic dysfunction and liver cholesterol metabolism within 48 h. This revealed minimal toxicity with rapid, widespread correction of gene expression, which correlated with improved serum biomarkers of liver injury, including alkaline phosphatase (ALKP) and albumin (ALB). To further characterize the biological response to treatment, we applied bulk RNA sequencing (RNA-seq) and a novel computational algorithm, MENTOR (Multiplex Embedding of Networks for Team-Based Omics Research), to integrate multiple sequencing datasets. This approach enabled the identification of gene modules, which were classified using a language learning model. To resolve the cellular origin of these effects, we performed single-nucleus RNA sequencing (snRNA-seq) to define cell-type-specific transcriptional changes across hepatic populations. Integration of snRNA-seq with bulk transcriptomic data enabled the identification of cell-type-specific corrected gene programs, linking global transcriptional normalization to discrete cellular components. This analysis identified large functional processes, which were corrected via mRNA treatment and reinstated pathways associated with Niemann-Pick type C1, which require further exploration. Together, these data demonstrate the utility of mRNA therapeutics in treating liver defects in Niemann-Pick type C1 disease and highlight the value of computational analysis for understanding therapeutic effects at a systems level.

RESULTS

Development of mRNA lipid particles

To begin proof-of-concept studies of mRNA therapeutics for Niemann-Pick type C1, mouse *Npc1* and *GFP* mRNA transcripts were synthesized, DNase-treated, and purified using silica gel. mRNA was synthesized with 5′-capping and full substitution with N1-methyl-pseudo-U to enhance transcript stability and reduce immunogenicity. Transcripts were assessed for size, purity, and concentration, revealing sharp, narrow peaks on a bioanalyzer, consistent with predominantly full-length transcripts at the expected sizes, ∼1,000 nucleotides (nt) for *GFP* and ∼3,800 nt for *Npc1* (Figure 1A). mRNA was encapsulated into LNPs using microfluidic mixing with purified lipid components. The lipid formulation included polyethylene glycol (1,2-dimyristoyl-rac-glycero-3-methoxypolyethylene glycol-2000, or DMG-PEG 2k)-lipids, ionizable lipids (SM-102), helper lipids (distearoylphosphatidylcholine; DSPC), and cholesterol. DMG-PEG 2k localizes to the surface of the particle and modulates particle size and colloidal stability by reducing nonspecific interactions.[11,20] Ionizable lipids remain neutral at physiological pH and become protonated in the acidic environment of the endosome, promoting endosomal escape and mRNA release.[11,21] Helper lipids were added to enhance particle stability, facilitate RNA release, and enhance lipid bilayer rigidity, and overall particle stability.[22,23] Particle size and uniformity were assessed using dynamic light scattering (DLS), which revealed a consistent size distribution centered around ∼100 nm (Figure 1B; Table S1). Polydispersity index (PDI) was measured to indicate the breadth of distribution of the particle sizes within the solution. The particles generated had a low PDI, indicating a uniform distribution of sizes. Encapsulation efficiency (EE) was determined to be greater than 96%, indicating that the vast majority of mRNA was successfully packaged within the lipid particles (Table S1). Transmission electron microscopy (TEM) confirmed the ∼100 nm diameter and spherical morphology of the particles, consistent with well-formed LNPs and efficient mRNA encapsulation (Figure S1).

HeLa cells were treated with *GFP*-loaded LNPs (*GFP*:LNP) for 24 h at a concentration of 0.15 ng/μL and a cell proliferation assay was used to determine toxicity. At this dose, particles were non-toxic as indicated by cellular metabolic activity measurements compared to untreated and H_2O_2-treated cells (Figure 1C). To evaluate transfection efficiency, HeLa cells were treated with *GFP*:LNP for 24 h and analyzed for fluorescence intensity via flow cytometry. Consistent with high quality particle generation, this revealed >99% of cells are expressing GFP 24 h after treatment (Figure 1D). Together, these measurements indicate that the mRNA-LNPs are homogenous, have

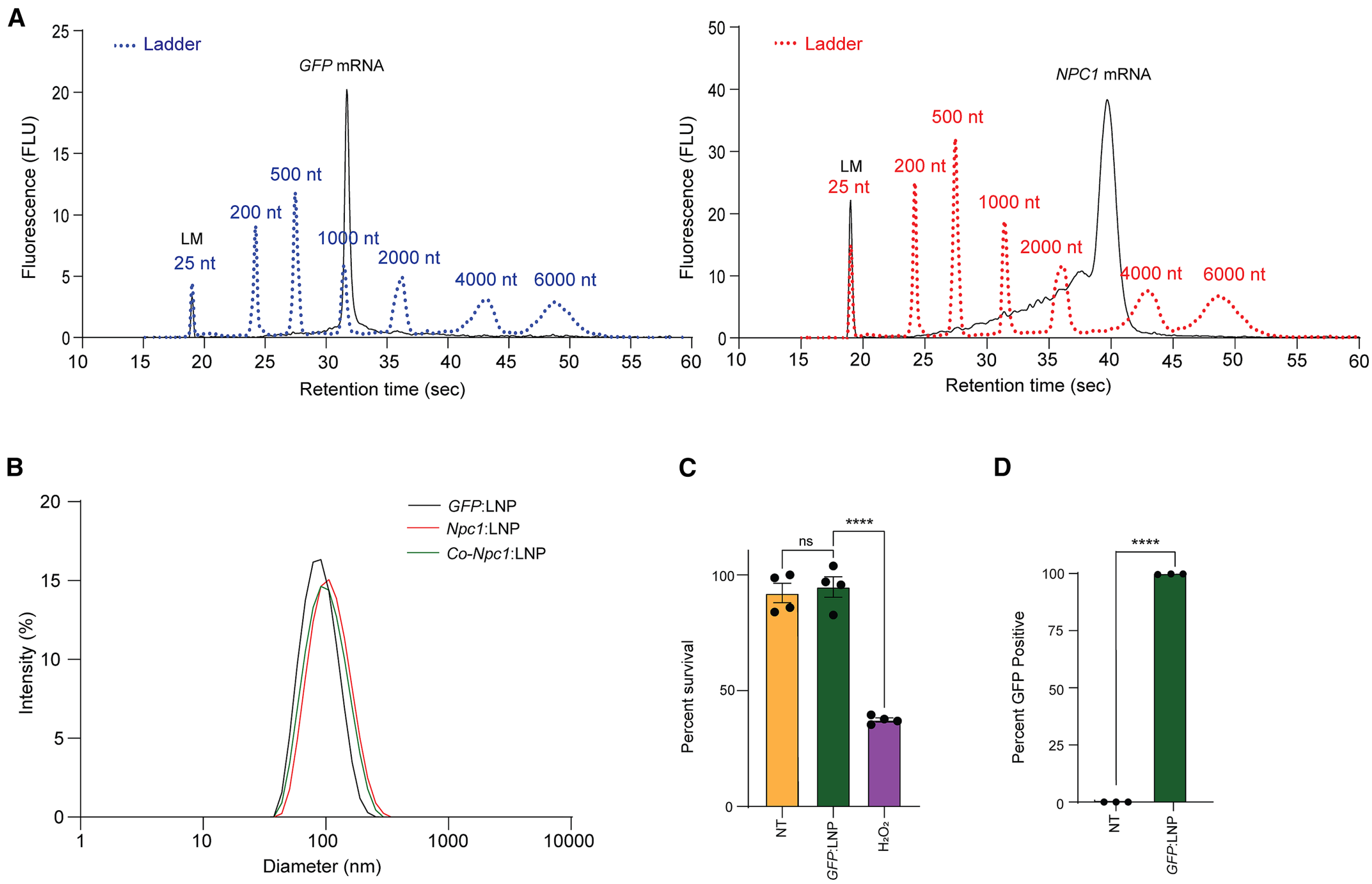

Figure 1. Synthesis and characterization of *Npc1* mRNA lipid particles

(A) Analysis of mRNA size, purity, and quantity using the Agilent 2100 Bioanalyzer for *GFP* (left) and *Npc1* (right). Dashed lines indicate ladder/standards with sizes indicated. LM, lower marker. (B) Size distribution for *GFP*:LNP, *Npc1*:LNP, and codon-optimized *Npc1* (*Co-Npc1*:LNP) as determined by dynamic light scattering. (C and D) Control HeLa cells were treated with lipid particles containing *GFP* mRNA (*GFP*:LNP) at a concentration of 0.15 ng/μL, no treatment (NT), or hydrogen peroxide (H_2O_2) for 24 h and analyzed for (C), percent survival by colorimetric analysis, or (D), GFP-positive cells determined by flow cytometry. ns, not significant; $^{*}p \leq 0.05$, $^{**}p \leq 0.01$, $^{****}p \leq 0.0001$ by (C), one-way ANOVA with Tukey's multiple comparisons test; F (2, 12) = 34.82, (D) unpaired *t* test; ($t = 2{,}950$, df = 4). Data are shown as the mean ± SEM from (C) $n = 5$/group, (D) $n = 3$/group.

high encapsulation efficiency (EE), and transfect cells *in vitro* with high efficiency.

Codon-optimized *Npc1*:LNPs correct primary and secondary Niemann-Pick type C1 cellular defects *in vitro*

To determine whether mRNA:LNP treatment could restore NPC1 protein expression in cells, we treated $NPC1^{-/-}$ HeLa cells with LNPs encapsulating mouse *Npc1* mRNA (*Npc1*:LNP) or with naked *Npc1* mRNA and assessed NPC1 protein levels by western blot 48 h post-treatment. *Npc1*:LNP treatment restored robust NPC1 protein expression, whereas naked *Npc1* mRNA failed to produce detectable NPC1 protein, demonstrating that LNP encapsulation is required for efficient *Npc1* mRNA delivery and translation (Figure S2). In addition, although N1-methyl-pseudouridine substitutions have been reported to induce ribosomal frameshifting and the production of aberrant peptide species, the NPC1 protein detected following *Npc1*:LNP treatment migrated at the expected molecular weight (Figure S2), consistent with correct translation of the encoded protein.[24]

Building on previous findings that codon usage can influence mRNA stability and protein output, we developed a codon-optimized version of *Npc1* mRNA (*Co-Npc1*:LNP).[25,26] Codon optimization involves replacing rare codons with synonymous codons that are more frequently used in the host, thereby increasing the utilization of cognate tRNAs and accelerating translation elongation.[27] To assess the persistence of *Npc1* transcripts following mRNA delivery, $NPC1^{-/-}$ HeLa cells were treated with either *Npc1*:LNP or *Co-Npc1*:LNP for 24 h, after which the media was replaced and *Npc1* transcript levels were quantified over time. *Npc1* transcript abundance declined progressively following treatment and no significant differences in transcript abundance were observed between *Npc1*:LNP and *Co-Npc1*:LNP treatments at any time point, indicating that codon optimization did not alter mRNA stability (Figure 2A).

NPC1 protein expression was evaluated over a 5-days washout period after a single 24-h treatment. *Co-Npc1*:LNP-treated cells exhibited consistently higher NPC1 protein levels compared to

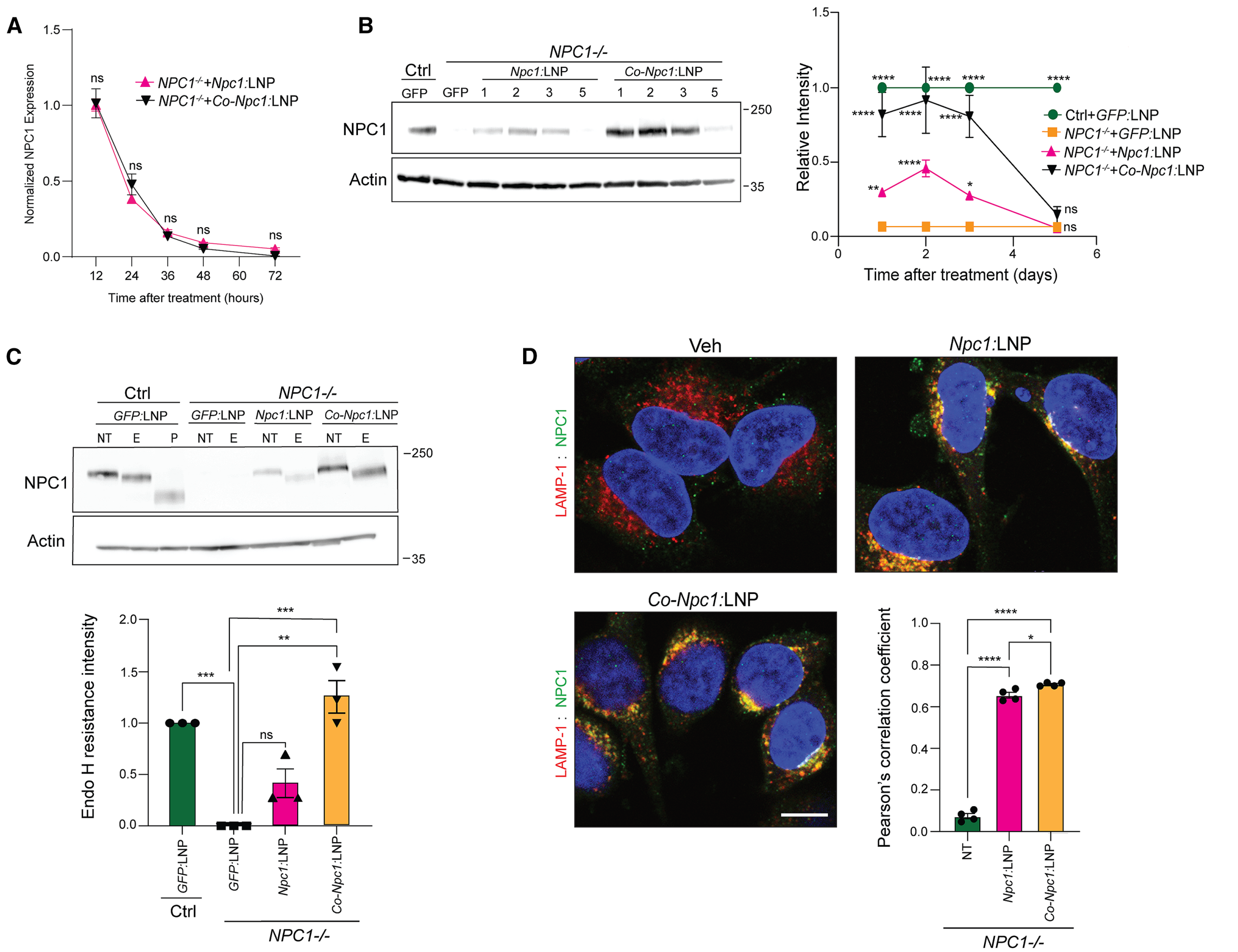

Figure 2. Codon optimization increases NPC1 protein levels

(A) *NPC1*$^{-/-}$ HeLa cells were treated with lipid particles containing *Npc1* (*Npc1*:LNP) or codon-optimized *Npc1* (*Co-Npc1*:LNP) for 24 h. After a media change, *Npc1* mRNA was analyzed 12, 24, 36, 48, 60, or 72 h later by RT-qPCR. (B) *NPC1*$^{-/-}$ or *NPC1*$^{+/+}$ cells were treated for 24 h with *GFP*:LNP, *Npc1*:LNP or *Co-Npc1*:LNP, then total NPC1 protein was analyzed 1, 2, 3, or 5 days later by western blot. NPC1 protein levels were normalized to β-actin. (C and D) Cells were treated for 24 h with lipid particles and assessed 24 h after a media change for localization by digestion with endoglycosidase H (E), PNGase F (P), or no treatment (NT), and analyzed by western blot. (D) Cells were treated for 24 h with lipid particles and assessed 24 h after a media change for localization using labeling for the lysosome marker LAMP-1 (red), NPC1 (green), and DAPI, then imaged via confocal microscopy. Pearson's colocalization coefficient was used to measure colocalization between LNP-delivered NPC1 or codon-optimized NPC1 and the lysosome. ns, not significant; $^{*}p \leq 0.05$, $^{**}p \leq 0.01$, $^{***}p \leq 0.001$, $^{****}p \leq 0.0001$ by (A and B) 2-way ANOVA with Bonferroni correction; (A) $F(4, 24) = 1.102$, (B) $F(9, 32) = 13.88$, or (C and D) one-way ANOVA with Tukey's multiple comparison test; (C) $F(3, 8) = 28.91$, (D) $F(2, 9) = 1007$. Data are shown as mean ± SEM from (A) $n = 5$/group, (B and C) $n = 3$/group, and (D) $n = 4$/group. (D) Scale bars, 10 μm.

Npc1:LNP-treated cells, confirming that codon optimization enhanced protein production (Figure 2B). Despite the robust increase in protein levels after *Npc1*:LNP and *Co-Npc1*:LNP treatment, the turnover rate of both NPC1 proteins remained similar and tracked closely with the degradation of mRNA over time (Figure 2B).

The NPC1 protein is a large (1,278 amino acids), heavily glycosylated lysosomal transmembrane protein that is prone to misfolding.[9,28–30] Therefore, careful analysis of NPC1 folding and cellular trafficking is essential to ensure therapeutic benefit. To assess this, we treated protein lysates with Endoglycosidase H (Endo H), an enzyme that cleaves immature high-mannose N-linked glycans from proteins. Misfolded NPC1 protein is retained in the endoplasmic reticulum (ER), rendering it sensitive to Endo H digestion due to failure to progress through the secretory pathway.[31] While there were differences in the total amount of both proteins, both the native and codon-optimized mRNAs yielded primarily Endo H resistant proteins, indicating correct folding and successful trafficking through the medial Golgi (Figure 2C).

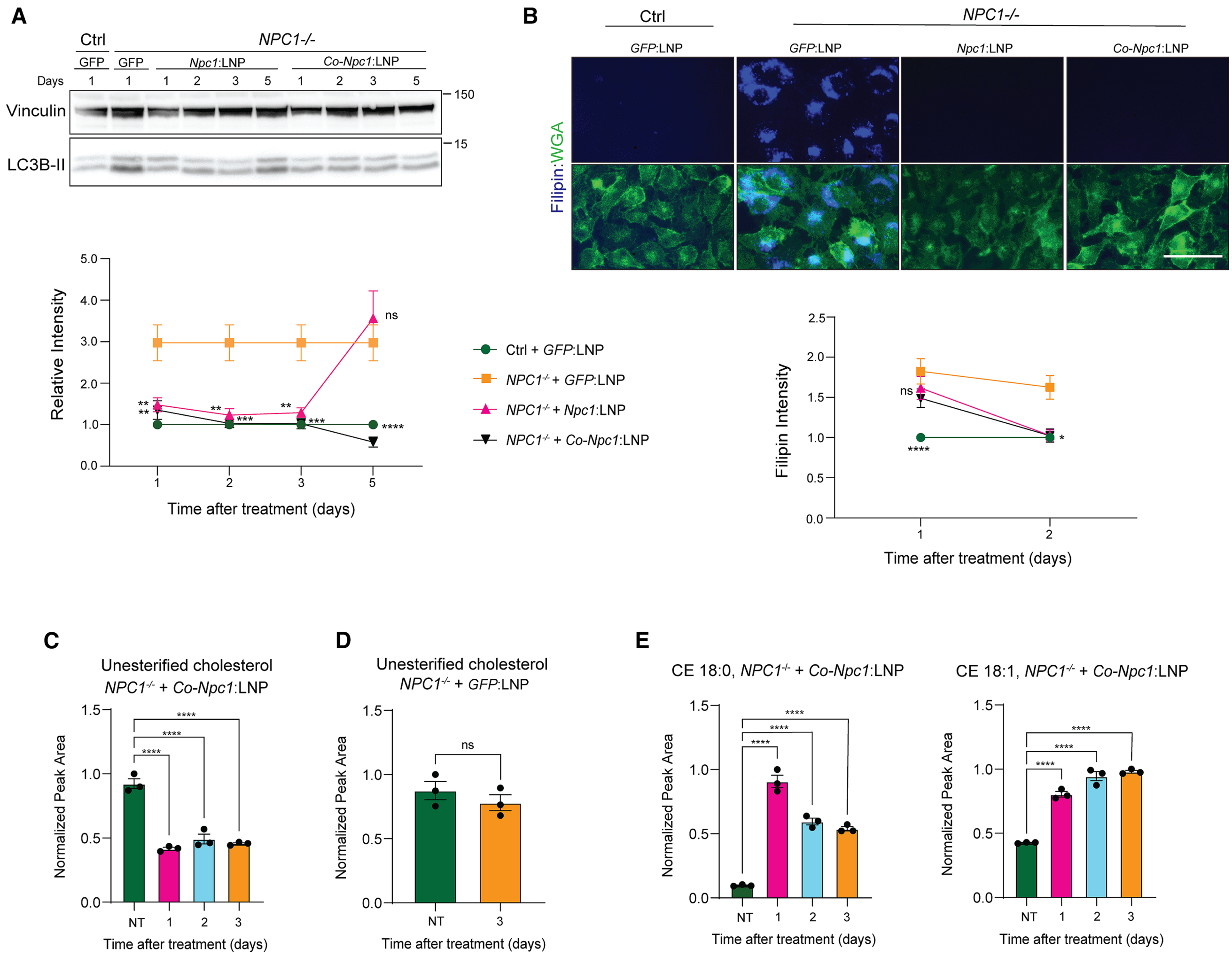

Figure 3. *Npc1* mRNA corrects primary and secondary Niemann-Pick type C1 cellular defects

(A and B) $NPC1^{-/-}$ and $NPC1^{+/+}$ (Ctrl) HeLa cells were treated for 24 h with lipid particles containing *GFP* (*GFP*:LNP), *Npc1* (*Npc1*:LNP) or codon-optimized *Npc1* (*Co-Npc1*:LNP) and, after a media change, were (A) analyzed for LC3B-II protein levels by western blot 1, 2, 3, or 5 days after a single treatment with LNPs. The upper band in the doublet is LC3B-I, whereas the lower band is LC3B-II. LC3B-II protein levels were normalized to vinculin. (B) Cells were stained and imaged 24 or 48 h post-treatment for cholesterol with Filipin (blue) and for cell membranes with wheat germ agglutinin (WGA) (green). $NPC1^{-/-}$ HeLa cells were treated for 1, 2, or 3 days with (C–E) *Co-Npc1*:LNP, or (E) *GFP*:LNP. Cells were then analyzed for (C and D) unesterified cholesterol, or (E) cholesteryl ester species via LC-MS. ns, not significant, $*p \leq 0.05$, $**p \leq 0.01$, $****p \leq 0.0001$ by (A and B) two-way ANOVA with Bonferroni correction; (A) F (9, 32) = 4.583, (B) F(3, 36) = 2.239, (C and E) one-way ANOVA with Tukey's multiple comparison test; (C) F (3, 8) = 69.03, (E) F (3, 8) = 132.3 and F (3, 8) = 139.6 from left to right, or (D) unpaired *t* test; (t = 18.09, df = 4). Data are shown as mean ± SEM from (A) n = 4–7/group, (B–E) n = 3/group. (A) Scale bars, 50 μm.

To determine whether the NPC1 proteins synthesized from mRNA:LNPs reached the proper intracellular destination, we assessed NPC1 colocalization with LAMP1, an established lysosomal marker. $NPC1^{-/-}$ HeLa cells were treated for 24-h and imaged 24 h later using confocal microscopy (Figure 2D). Pearson's colocalization coefficient (0.707) revealed significant overlap between NPC1 and LAMP1 signals, indicating successful trafficking of the NPC1 protein to the lysosome (Figure 2D).

Niemann-Pick type C1 is associated with several secondary cellular defects including defective autophagy, resulting in accumulation of the autophagic marker LC3B-II.[32–34] Once autophagy is initiated, cytosolic lipidated LC3B-II is recruited to autophagosomal membranes and becomes a hydrophilic, faster migrating band on a western blot which appears as the lower band of the LC3B doublet.[35] We treated cells for 24 h then assessed LC3B-II over a 5-days wash out period, corresponding to the duration of NPC1 protein expression following mRNA:LNP treatment (Figure 2B). As expected, $NPC1^{-/-}$ cells exhibited elevated LC3B-II levels relative to control cells. Treatment with either *Npc1*:LNP or *Co-Npc1*:LNP normalized LC3B-II levels within 24 h, consistent with reduced autophagosome accumulation following restoration of NPC1 function (Figure 3A).

However, the durability of this correction differed between formulations. LC3B-II levels in *Npc1*:LNP-treated cells progressively increased and returned to levels observed in vehicle-treated *NPC1*$^{-/-}$ cells by day 5, whereas *Co-Npc1*:LNP-treated cells maintained reduced LC3B-II levels throughout the 5-days washout period (Figure 3A).

NPC1 loss-of-function causes the accumulation of unesterified cholesterol, which can be labeled using a filipin stain.[36,37] *NPC1*$^{+/+}$ (Ctrl) and *NPC1*$^{-/-}$ cells were stained with filipin 24- and 48-h post-LNP treatment (Figure 3B). As expected, *NPC1*$^{-/-}$ cells have increased levels of filipin staining compared to the *NPC1*$^{+/+}$ control. Importantly, treatment of *NPC1*$^{-/-}$ cells with either *Npc1*:LNP or *Co-Npc1*:LNP reduced filipin fluorescence at 48 h (Figure 3B). There was no significant difference between the *Npc1* or *Co-Npc1* constructs, likely reflecting the initial, large bolus of protein produced by both formulations. This reduction in filipin signal indicates the trafficked NPC1 is functionally active and restores cholesterol trafficking.

To determine whether this observed clearance of cholesterol is maintained over time, we measured levels of unesterified cholesterol using liquid chromatography-mass spectrometry (LC-MS) one, two, and three days after *Co-Npc1*:LNP treatment. This revealed a significant drop in unesterified cholesterol compared to the untreated *NPC1*$^{-/-}$ cells that was maintained at every time point (Figure 3C). To ensure that cholesterol present in the low dose LNP formulation did not confound this effect, we treated *NPC1*$^{-/-}$ cells with *GFP*:LNP and observed no increase in unesterified cholesterol levels three days after treatment (Figure 3D). To determine whether restoring lysosomal delivery of NPC1 re-establishes cholesterol flux, we measured cholesteryl ester levels and observed a significant increase following *Co-Npc1*:LNP treatment in *NPC1*$^{-/-}$ cells, consistent with enhanced trafficking of cholesterol to the ER for re-esterification (Figure 3E). These results indicate that both *Npc1* mRNA:LNP formulations can transiently correct autophagic and cholesterol flux in NPC1-deficient cells. However, the codon-optimized *Co-Npc1*:LNP provides a more sustained correction, likely due to prolonged NPC1 protein expression, making this formulation the preferred candidate for *in vivo* testing.

Codon-optimized *Npc1*:LNPs target the liver and correct primary and secondary Niemann-Pick type C1 cellular defects *in vivo*

Next, we sought to determine whether the phenotypic corrections seen *in vitro* translate into an *in vivo* mouse model. To evaluate particle biodistribution, we employed a reporter system wherein the LNP delivered a firefly luciferase (*LUC*) mRNA via the tail vein at a dose of 1 mg/kg. 24 h later, mice were injected with D-luciferin potassium salt substrate to initiate luminescence and imaged on an *In Vivo* Imaging System (IVIS) to validate targeting of the particle. Robust luminescent signals were detected in the liver region at 24 h post-injection, confirming efficient liver targeting by the LNP formulation (Figure S3). We also tested whether *Co-Npc1*:LNP particle delivery activated the immune response by macrophage cell staining. There was no measurable change in macrophage-positive staining between the *GFP*:LNP and *Co-Npc1*:LNP-treated *Npc1*$^{-/-}$ groups (Figure S4), 48 h post-injection.

Next, we expanded testing into 3-weeks-old *Npc1*$^{+/+}$ and *Npc1*$^{-/-}$ mice to avoid confounding effects related to end-stage disease. Mice received a single i.v. injection of 1 mg/kg *Co-Npc1*:LNP or saline (Veh), and livers were harvested 48 h post-injection for analysis. *Co-Npc1*:LNP treatment significantly increased NPC1 protein levels in the liver of *Npc1*$^{-/-}$ mice, though did not match *Npc1*$^{+/+}$ levels (Figure 4A). All *Co-Npc1*:LNP-treated mice exhibited Endo H-resistant NPC1 protein, indicating effective ER escape/lysosomal trafficking of *Co-Npc1*:LNP-encoded NPC1 *in vivo* (Figure 4B). Similar to what was seen *in vitro*, a single treatment with *Co-Npc1*:LNP normalized expression of the autophagic marker LC3B-II (Figure 4C). These findings demonstrate that *Co-Npc1*:LNP-derived NPC1 protein corrects key phenotypic defects associated with Niemann-Pick type C1 in the mouse liver.

We characterized the extent of liver correction by performing liver function tests on 6-weeks-old mice, four days after a single *Co-Npc1*:LNP injection. At this early 6-weeks time point, liver function markers are not fully elevated in *Npc1* knockout models.[38,39] Both ALB and ALKP levels were significantly reduced in *Npc1*$^{-/-}$ mice treated with *Co-Npc1*:LNP compared to a saline-treated *Npc1*$^{-/-}$ control, while alanine aminotransferase (ALT), aspartate aminotransferase (AST), and total bilirubin (TBIL) levels remained unchanged (Figures 4D and S5). The change in ALB and ALKP levels only 4 days after a single injection suggests promising results for more long-term, multi-dose studies.

Codon-optimized *Npc1*:LNP-treated livers show increased cholesteryl esters in the lipidome

We quantified cholesterol levels in disease-state and LNP-treated *Npc1*$^{-/-}$ mice using LC-MS. *GFP*:LNP treatment in *Npc1*$^{-/-}$ mice showed an increase in both unesterified cholesterol and the synthetic lipid SM-102, indicating that LNP-derived lipids contribute to the measured cholesterol pool (Figures 5A and S6). This effect is likely due to the nanoparticle composition and the relatively high *in vivo* dosing, which may delay hepatic clearance of LNP-derived lipids. Accordingly, a portion of the measured cholesterol signal may reflect LNP-associated lipid burden rather than intrinsic defects in cholesterol homeostasis. To control for this formulation-driven effect, we directly compared Co-*Npc1*:LNP to *GFP*:LNP controls, which share an identical lipid composition but differ in therapeutic or control cargo. This approach isolates NPC1-dependent effects on cholesterol handling from non-specific lipid contributions associated with LNP delivery.

We observed increased levels of four cholesteryl ester species in *Co-Npc1*:LNP-treated livers, indicating enhanced cholesterol esterification after treatment (Figure 5B). *GFP*:LNP treatment did not increase esterification, showing that improved esterification and the

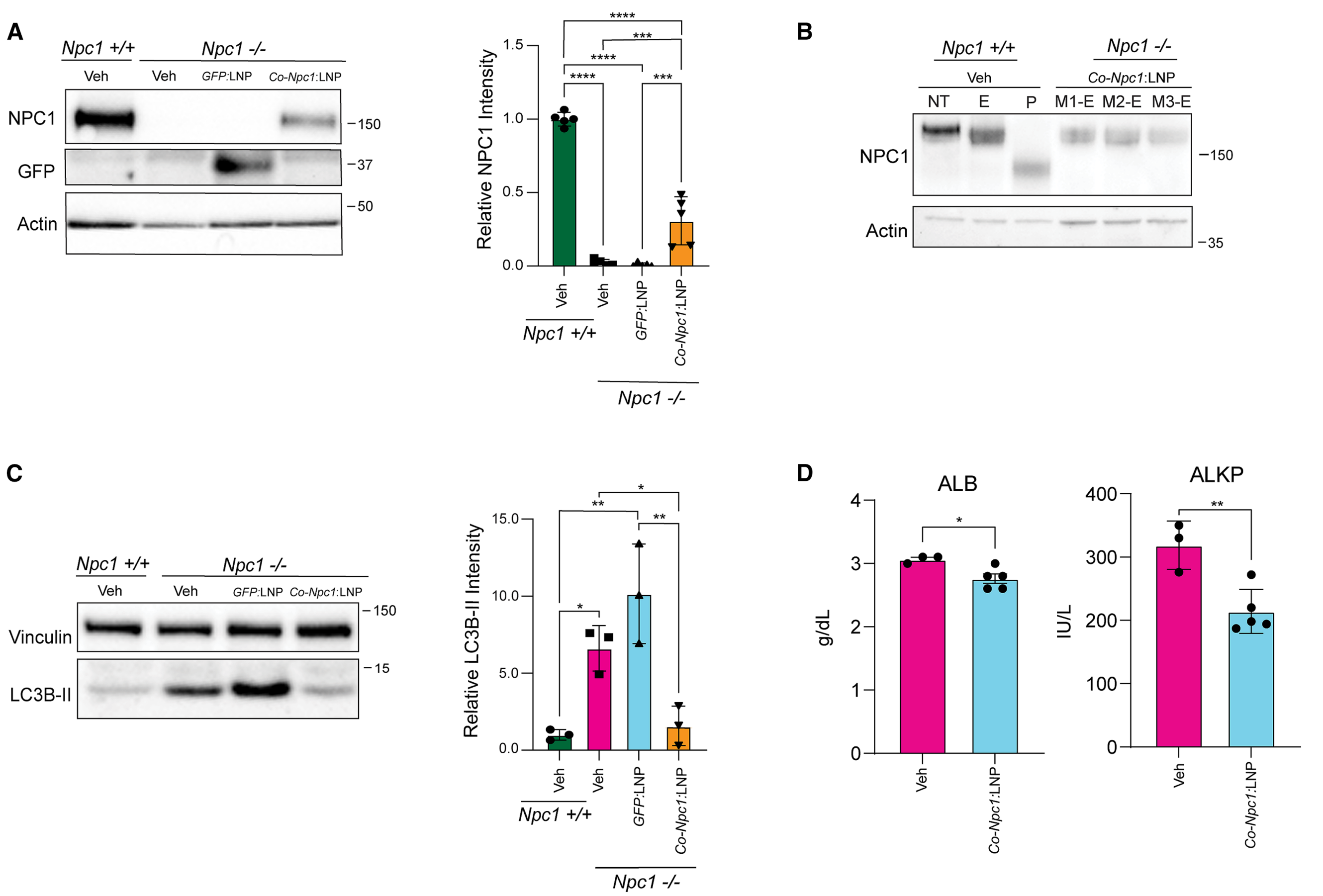

Figure 4. Lipid particle-mediated delivery of *Npc1* mRNA to *Npc1*$^{-/-}$ mice increases NPC1 protein and corrects secondary phenotypes

(A–D) Around 3-weeks-old *Npc1*$^{+/+}$ (WT) and *Npc1*$^{-/-}$ mice were injected once intravenously with saline (Veh), 1 mg/kg of an LNP containing *GFP* mRNA (*GFP*:LNP), or an LNP containing codon-optimized *Npc1* mRNA (*Co-Npc1*:LNP). Livers were collected after 48 h after treatment. Liver lysates were analyzed for (A) total NPC1 protein, and (B) EndoH sensitivity. NT, no treatment; E, EndoH treatment; P, PNGase F treatment. M1, M2, M3 refer to individual mice. NPC1 protein levels were normalized to β-actin. (C) Analysis of LC3B-II via western blot. LC3B-II protein levels were normalized to vinculin. (D) Six-weeks-old *Npc1*$^{-/-}$ mice were injected once intravenously with 200 μL saline or 1 mg/kg of *Co-Npc1*:LNP. After 4 days, livers were harvested and analyzed for levels of the enzymes ALKP and ALB. *$p \leq 0.05$, **$p \leq 0.01$, ***$p \leq 0.001$, ****$p \leq 0.0001$ by (A and C) one-way ANOVA with Tukey's multiple comparisons test; (A) F (3, 16) = 145.7, (C) F (3, 12) = 30.78, or (D) unpaired *t* test; (t = 2.986, df = 6), (t = 3.989, df = 6), from left to right. Data are shown as the mean ± SEM from (A) *n* = 5/group, (B) *n* = 5–6/group, (C and D) *n* = 3–5/group.

reduction in free cholesterol occur only when *Co-Npc1*:LNP drives NPC1 production. Consistent with restored cholesterol trafficking, *Hmgcs1*, a cholesterol regulatory gene upregulated in Niemann-Pick type C1, showed decreased expression in *Co-Npc1*:LNP-treated mice (Figure 5C). This suggests that cholesterol pools in *Co-Npc1*:LNP-treated mice reached sterol-sensing machinery and suppressed chronic *Hmgcs1* transcription seen in the disease state. Together, these data indicate that NPC1 produced from *Co-Npc1*:LNP restores cholesterol trafficking and corrects Niemann-Pick type C1 defects beyond merely lowering lysosomal cholesterol.

MENTOR and MENTOR-IA analysis of transcriptional correction following mRNA treatment

To elucidate and visualize the extent of Co-*Npc1*:LNP effects after a single treatment in mouse liver, we performed bulk RNA-seq on liver tissue. This revealed 714 differentially expressed genes (DEGs) for the comparison of *Co-Npc1*:LNP-treated *Npc1*$^{-/-}$ vs. *GFP*:LNP-treated *Npc1*$^{-/-}$ mice, and of those DEGs, 645 had opposite directionality to the comparison of *GFP*:LNP-treated *Npc1*$^{-/-}$ vs. saline-treated *Npc1*$^{+/+}$ mice. This list of 714 unique DEGs was analyzed using MENTOR, a network-based multi-omics integration tool, which reveals coordinated biological responses rather than isolated gene-level changes. MENTOR organizes DEGs into functionally related clusters, or "modules," based on a random walk with restart (RWR) exploration on a multiplex network.[40] This approach enables the identification of biologically meaningful relationships between genes that share similar functional or regulatory contexts, providing a systems-level view of how treatment impacts interconnected pathways. MENTOR identified 32 modules of functionally related genes (Figure S7). The outermost heatmap track in Figure S7 denotes module-level statistical

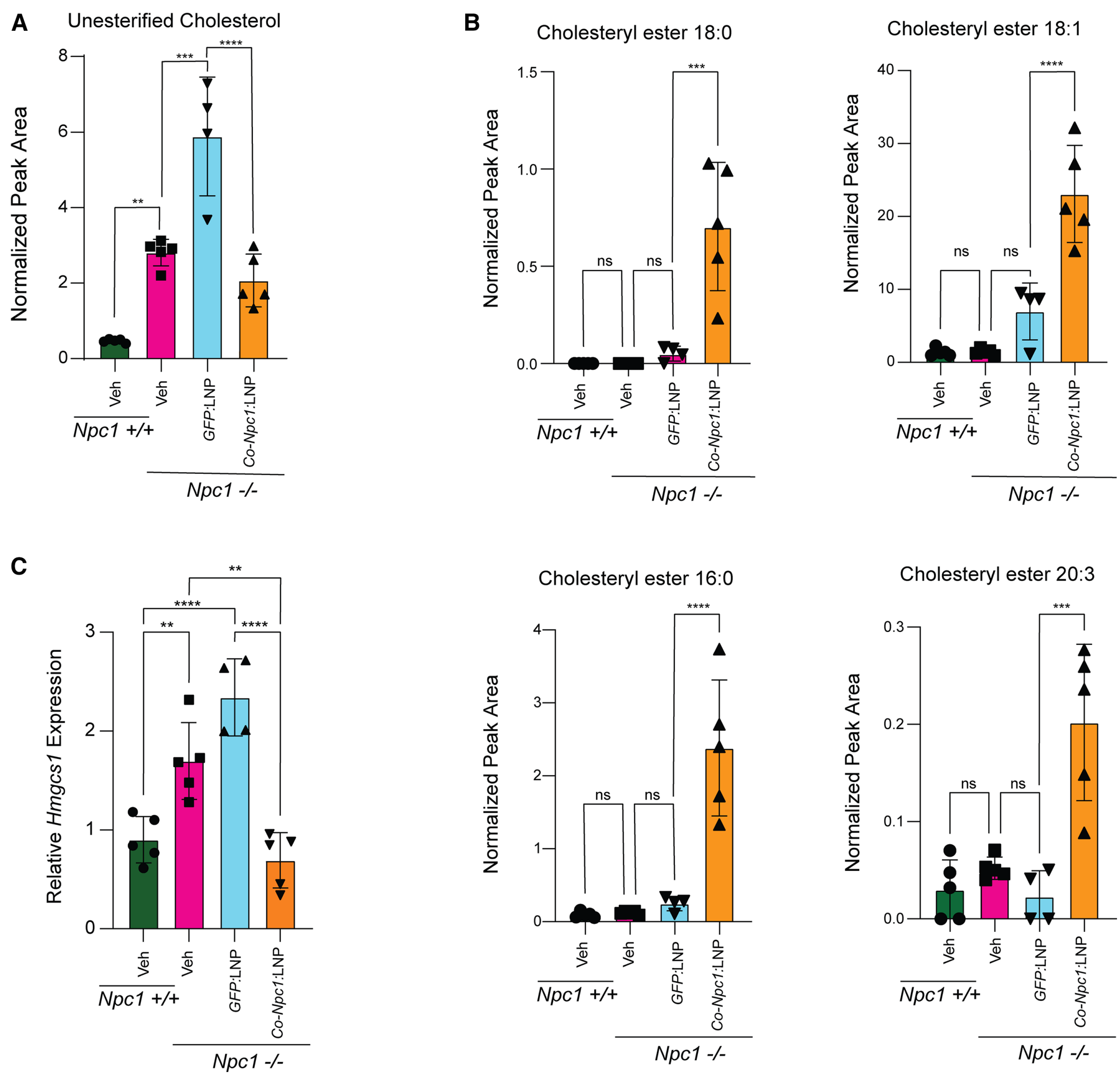

Figure 5. Codon-optimized LNP restores cholesterol esterification in *Npc1*$^{-/-}$ mice

Three-weeks-old *Npc1*$^{+/+}$ (WT) and *Npc1*$^{-/-}$ mice treated with saline (Veh), 1 mg/kg *GFP* mRNA (*GFP*:LNP), or 1 mg/kg codon-optimized *Npc1* mRNA (*Co-Npc1*:LNP). Livers were collected 48 h after treatment. (A and B) Levels of cholesteryl esters 18:0, 18:1, 16:0, 20:3, or unesterified cholesterol were analyzed by LC-MS. (C) *Hmgcs1* mRNA levels measured by RT-qPCR. *$p \leq 0.05$, **$p \leq 0.01$, ***$p \leq 0.001$, ****$p \leq 0.0001$ by one-way ANOVA with Tukey's multiple comparisons test; (A) F (3, 15) = 34.28, (B) F (3, 15) = 19.97, F (3, 15) = 35.09, F (3, 15) = 26.25, F (3, 15) = 16.11, from left to right, top to bottom, and C F (3, 15) = 24.09. Data are shown as the mean ± SEM from n = 4–5/group.

significance, with black segments indicating modules that are significant relative to permutation-derived null distributions. Notably, most modules are statistically significant, supporting the robustness and biological organization of the identified gene modules. The innermost track highlights gene expression changes between *Co-Npc1*:LNP-treated *Npc1*$^{-/-}$ vs. saline-treated *Npc1*$^{+/+}$ mice, where a gray $\log_2$ fold-change value illustrates correction of gene expression levels by *Co-Npc1*:LNP towards saline-treated *Npc1*$^{+/+}$ mice levels. For example, in module 4, *Hmgcs1* is downregulated following Co-*Npc1*:LNP treatment and corrected towards saline-treated *Npc1*$^{+/+}$ levels, consistent with restored cholesterol homeostasis.

To interpret each module's biological function, we applied the MENTOR-Interpretation Agent (MENTOR-IA) tool, which integrates gene expression data with multiple external databases.[41] MENTOR-IA utilizes the gene expression data and shortest paths analysis of the multiplex for each module to then query multiple application programming interfaces (APIs) including gene set enrichment analysis (GSEA) (for Gene Ontology, GO enrichment), mygene.info (for gene functions), and EBI (for relevant literature). The information gathered for the genes within a module is utilized with a task prompt to query the Microsoft Azure API, which returns a summary of the biological functions implicated within the

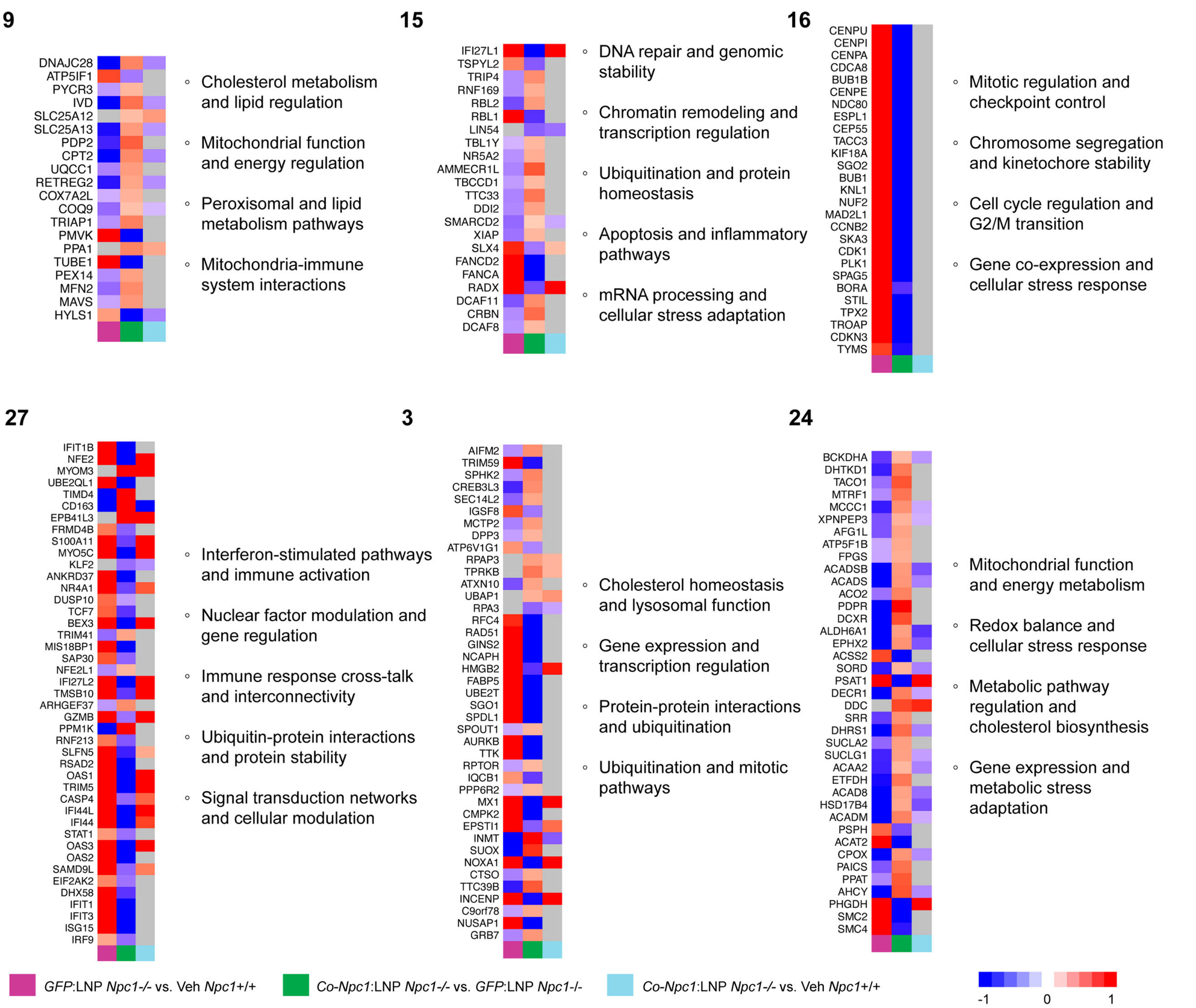

Figure 6. *Co-Npc1*:LNP-treatment in mice results in transcriptional changes

Three-weeks-old $Npc1^{+/+}$ and $Npc1^{-/-}$ mice treated with saline (Veh), *GFP* mRNA (*GFP*:LNP), or codon-optimized *Npc1* mRNA (*Co-Npc1*:LNP). Livers were collected 48 h after treatment and homogenized for bulk RNA-seq. Sequencing results were interpreted using MENTOR and MENTOR-IA analysis. (A–F) shows selected functional modules from MENTOR analysis and the respective MENTOR-IA thematic interpretation. $n = 3$/group.

module. By combining these data streams, MENTOR-IA provides unbiased, systems-level summaries of functional pathways, mitigating limitations of conventional ontology-based enrichment. The MENTOR-IA analysis revealed distinct modules representing restoration across multiple cellular and tissue processes (Figure 6; Table S2). From Figure 6, module 3 included pathways critical for NPC1 correction, such as cholesterol homeostasis and lysosomal function, while modules 15 and 16 reflected normalization of stress-response, homeostatic, and cell cycle pathways. Module 24 showed recovery of metabolic regulation, including energy metabolism, consistent with improved cholesterol processing. Notably, modules enriched for mitochondrial and peroxisomal genes, modules 24 and 9, demonstrated marked restoration toward $Npc1^{+/+}$ levels. Transcriptional normalization of both peroxisomal and mitochondrial genes in module 9 suggests a previously underappreciated transcriptomic association between mitochondria and peroxisomal processes in Niemann-Pick type C1 pathogenesis and response to mRNA-based correction. The shift toward $Npc1^{+/+}$ transcriptional levels for *Co-Npc1*:LNP-treated $Npc1^{-/-}$ mice in the immune-related module 27 suggests both a rescue of immune response caused by Niemann-Pick type C1 and low immunogenicity of the mRNA:LNP treatment. Overall, nearly every module

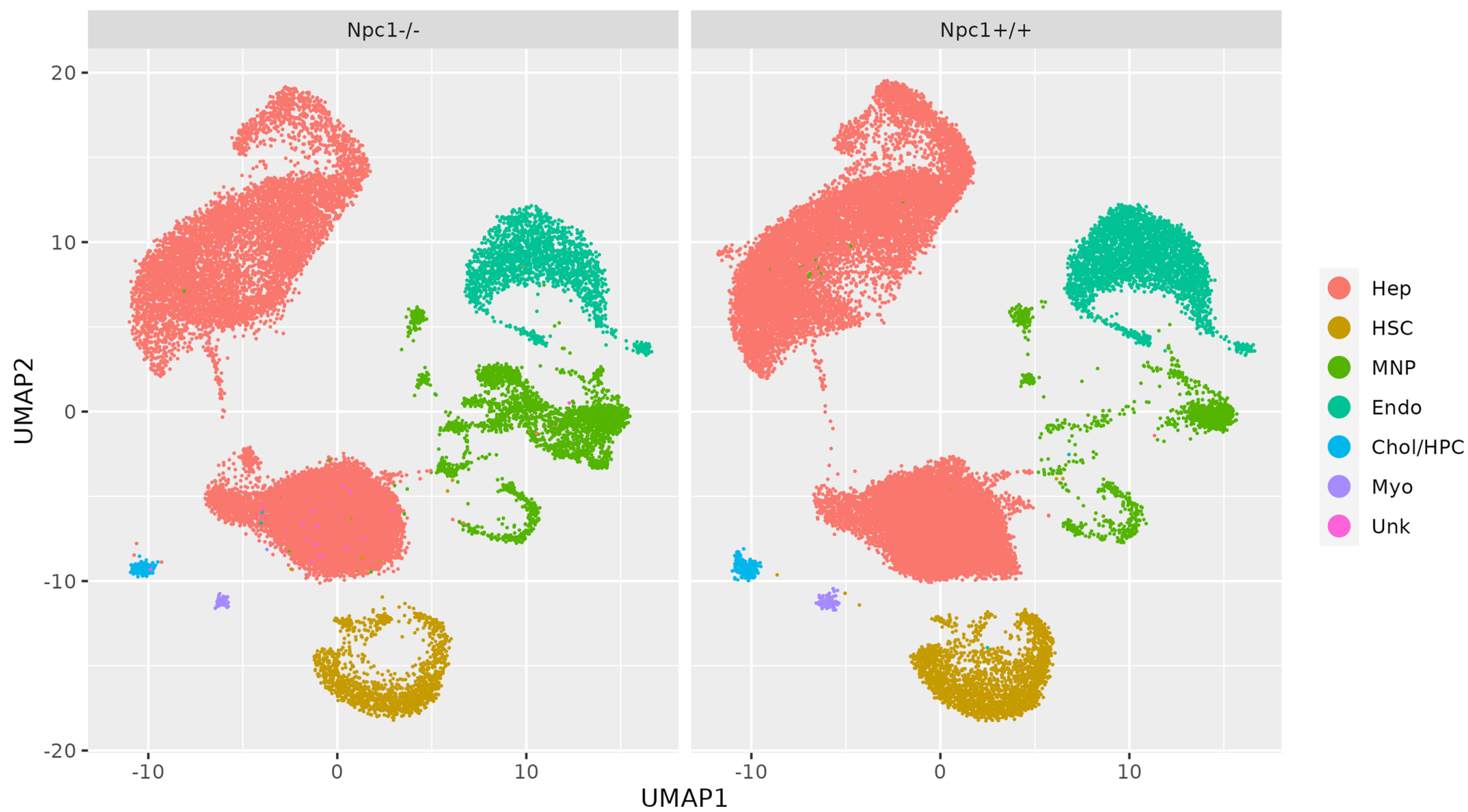

Figure 7. Major cell type populations identified in snRNA-seq data

UMAP embeddings displaying results of Seurat dimensionality reduction, clustering, and differential expression analysis for manual annotation of major cell type populations. Cell types identified include hepatocytes (Hep), hepatic stellate cells (HSC), mononuclear phagocytes (MNP), endothelial cells (Endo), cholinergic/hepatic progenitor cells (Chol/HPCs), myofibroblasts (Myos), and a small population of unknown cells (Unks). Cell type clusters are preserved across genotypes.

showed a significant shift of *Co-Npc1*:LNP-treated *Npc1*$^{-/-}$ mice toward *Npc1*$^{+/+}$ gene expression profiles (Figure S7).

We performed snRNA-seq on livers of *Npc1*$^{-/-}$ mice and littermate controls at 7 weeks of age (n = 3 per genotype) and integrated these data with MENTOR to map disease-associated, cell type-specific transcriptional changes. Integration across individual samples followed by quality control yielded 58,673 high-quality nuclei across both genotypes (Figures S8 and S9). Dimensionality reduction using uniform manifold approximation and projection (UMAP) and clustering revealed well-separated clusters corresponding to major hepatic cell populations, with consistent clustering observed between *Npc1*$^{-/-}$ and *Npc1*$^{+/+}$ conditions (Figures 7 and S10). Expression patterns of established marker genes were used to identify cell type-specific clusters encompassing the major liver cell populations (Figure S11; Table S3).[42–50] Seven primary cell types were identified including Hep, hepatic stellate cells (HSCs), mononuclear phagocytes (MNPs), endothelial cells (Endo), cholangiocytes/hepatic progenitor cells (Chol/HPCs), myofibroblasts (Myos), and a small population of unclassified cells (Unks). Of these seven cell types, only MNPs and Myos showed a significant increase in abundance, consistent with NPC1 deficiency resulting in expansion of hepatic MNPs, enhanced inflammatory responses, and immune cell recruitment (Table S4).

Differential expression (DE) analysis identified DEGs between *Npc1*$^{-/-}$ and *Npc1*$^{+/+}$ conditions across all cell types. Additional MENTOR heatmap tracks were used to incorporate snRNA-seq DEGs across Hep, MNP, and HSC displaying $\log_2$ fold-change values for each cell type and enabling visualization of cell type-specific transcriptional changes within each module, as well as DEGs unique to each cell type that were corrected in the bulk RNA-seq dataset (Figure S7). Quantification of unique cell type-specific DEGs revealed that Heps exhibited the largest number of unique DEGs (n = 78), followed by MNPs (n = 51) and HSCs (n = 19), indicating dominant contribution of Heps to transcriptional alterations. A substantial proportion of cell type-specific DEGs were also reflected as corrected in the bulk RNA-seq data following treatment, including 45 Hep-specific DEGs (58%), 33 MNP-specific DEGs (65%), and 14 HSC-specific DEGs (74%), reflecting the ability of *Co-Npc1*:LNPs to regulate the transcriptional shift of multiple relevant cell types (Table 1).

DISCUSSION

Early hepatic involvement in children with Niemann-Pick type C1 contributes to systematic metabolic disruption and may compromise patient stability during key developmental windows. As such, targeting the liver could provide early, foundational support to improve outcomes even before central nervous system symptoms fully emerge. mRNA-mediated treatment serves as an attractive approach to treat monogenic diseases such as Niemann-Pick type C1 due to the transient nature and strong safety profile. Here, we show the first mRNA replacement strategy for Niemann-Pick type C1, which temporally restores NPC1 protein.

Table 1. Cell type-specific transcriptional changes

Cell type	Unique DEGs	Unique DEGs with correction	Percent unique DEGs corrected
Hepatocytes	78	45	58
Hepatic stellate cells	19	14	74
Mononuclear phagocytes	51	33	65

Summary of three cell types of interest and the number of DEGs unique to each respective cell type uncovered in both the bulk and snRNA-seq datasets. "Unique DEGs with correction" describe the DEGs from *Co-Npc1*:LNP-treated *Npc1* $^{-/-}$ mice which show no difference in expression compared to vehicle/saline-treated *Npc1*$^{+/+}$ mice.

In this study, we demonstrate that a single dose of *Co-Npc1*:LNP, an mRNA-loaded LNP, corrects molecular defects of Niemann-Pick type C1 *in vitro* (Figures 2 and 3). The *Npc1* transcript used in this formulation was codon-optimized for murine expression to enhance translation efficiency and promote accurate protein folding. These optimizations likely contributed to the rapid and potent therapeutic effects observed in Figures 2 and 3. We furthered our study and tested *Co-Npc1*:LNP *in vivo*, finding potential for liver correction (Figures 4, 5, and 6). *Co-Npc1*:LNP restored essential aspects of NPC1 biology, including proper NPC1 protein localization and cholesterol trafficking both *in vitro* and *in vivo* (Figures 2, 3, 4, and 5). After *in vivo* administration, *GFP*:LNP-treated *Npc1*$^{-/-}$ mice exhibited an increase in unesterified cholesterol, but not esterified cholesterol, along with an increase in the synthetic lipid species SM-102 (Figures 5 and S6). In contrast, *Co-Npc1*:LNP-treated *Npc1*$^{-/-}$ mice showed an increase in esterified cholesterol and SM-102, but not unesterified cholesterol. These findings suggest that while LNP delivery contributes to accumulation of synthetic lipid components, restoration of NPC1 function shifts cholesterol toward esterified pools, consistent with improved intracellular trafficking and processing. We observed no apparent hepatic inflammation following mRNA:LNP administration, suggesting that lipid loading from a single dose does not exacerbate the Niemann-Pick type C1 phenotype; however, longer term studies are needed to assess potential cumulative effects. Overall, we saw corrections of key disease hallmarks, including NPC1 protein levels, autophagic flux, and cholesterol flux.

For investigation into systematic correction of the Niemann-Pick type C1 hepatic phenotype, we measured liver injury markers and found improvement in the treatment group compared to untreated *Npc1*$^{-/-}$ mice (Figures 4 and S5). To assess the broad transcriptional impact of treatment, we performed bulk RNA-seq and applied MENTOR, a network-based multi-omics integration tool (Figure S7; Table S2). MENTOR grouped DEGs into functionally related modules and visualized gene expression patterns across experimental conditions. These gene clusters were further interpreted by MENTOR-IA, a tool that utilizes network topology, gene-level information derived from multiple APIs, and the Microsoft Azure API to generate summaries of the biological functions implicated within each MENTOR module (Figure 6). This analysis suggested a partial restoration of *Npc1*$^{+/+}$-like expression patterns following treatment, with some modules showing more than 50% of genes with this transcriptional shift (Figure S7).

Importantly, MENTOR-IA revealed novel insights into the pathogenesis of Niemann-Pick type C1 liver disease. Prior studies have shown cholesterol accumulation in mitochondrial membranes resulting in impaired ATP production in brain tissue (Figure 6).[51] Along these lines, MENTOR-IA identified a gene network linking peroxisomal genes to key biological processes, including cholesterol metabolism and mitochondrial function (Figure 6). Although peroxisomal dysfunction has been reported in Niemann-Pick C1, its molecular basis remains poorly defined.[52] This connection between peroxisomal and mitochondrial pathways could imply that these processes may represent a unified metabolic response to cholesterol dysregulation, rather than independent, or loosely linked downstream effects. Together, these findings suggest that effective LNP correction in Niemann-Pick type C1 extends beyond restoring cholesterol homeostasis, encompassing coordinated correction of multiple pathways and system-level improvements.

Furthermore, MENTOR-IA provides a powerful framework for evaluating therapeutic efficacy from transcriptomic data. By quantifying the extent of pathway normalization across distinct biological functional modules, this approach enables objective comparison of how candidate therapies restore disease-altered pathways (Table S2). This feature is particularly valuable for complex disorders, such as Niemann-Pick type C1, where altered cholesterol homeostasis disrupts critical cellular processes. Therefore, therapeutic benefit likely involves coordinated correction of multiple pathways rather than targeting an isolated gene-level effect.

We report the first snRNA-seq dataset comparing *Npc1*$^{+/+}$ and *Npc1*$^{-/-}$ mouse liver and leverage this resource to resolve cell type-specific transcriptional changes underlying the bulk RNA-seq response to *Co-Npc1*:LNP treatment. Integration of these datasets revealed that greater than 50% of Heps, HSCs, and MNPs exhibit transcriptional shifts toward a wild-type/*Npc1*$^{+/+}$- like state following treatment, providing cell-type specific evidence of therapeutic correction (Table 1). Importantly, this dataset establishes a framework for deconvolving bulk transcriptomic responses and enables more precise attribution of therapeutic effects to specific liver cell populations.

Given the limited understanding of liver-specific pathology in Niemann-Pick type C1, these data address a critical gap by defining how NPC1 deficiency and disrupted cholesterol flux remodel the hepatic transcriptional landscape across multiple cellular compartments (Figures 7 and S7; Table 1). As an accessible resource, this snRNA-seq dataset will support future mechanistic studies and guide the development of targeted interventions aimed at correcting cell type–specific pathological processes in Niemann-Pick type C1-related liver disease.

Our work here focuses only on the effects of a single LNP:mRNA administration to determine whether this approach is a viable option for treating Niemann-Pick type C1. LNP-delivered mRNA therapies are gaining traction for monogenic diseases, particularly those characterized by substrate accumulation and progressive tissue degeneration, such as Fabry disease, Gaucher disease, and glycogen storage disease type 3.[12,14,53] LNP:mRNA platforms offer several advantages including high protein expression, minimal immunogenicity, repeat dosing potential, and reversibility in the event of adverse effects.[12–14] While our current formulation demonstrates robust hepatic delivery, as supported by liver luminescence data indicating predominant uptake in the liver, future studies will require refinement of tissue- and zone-specific targeting within Heps to optimize therapeutic distribution (Figure S3).

Moreover, as Niemann-Pick type C1 is a multisystem disorder affecting both peripheral organs and the central nervous system, broader biodistribution will be necessary for full disease correction. Prior studies have demonstrated that LNP systems can be engineered to target additional tissues, including brain, spleen, and lung, which are also relevant to disease pathology and warrant further investigation in the context of *Co-Npc1* mRNA delivery.[54–56] Further optimization of ionizable lipid components may also improve endosomal escape efficiency while reducing off-target immune activation and lipid-associated toxicity, which remain key considerations for clinical translation.[57,58] While we observed minimal immunogenicity with a single treatment, the effects of repeated dosing with optimized formulations that enhance tissue targeting and intracellular delivery will need to be carefully evaluated in future studies (Figure S4).

We targeted the liver as a proof-of-concept to improve pathology in Niemann-Pick type C1 and address a major source of early disease morbidity. However, durable clinical benefit will ultimately require strategies that also achieve effective delivery to the central nervous system to treat neurological manifestations. Development of approaches capable of coordinated hepatic and brain targeting will therefore be essential for comprehensive disease correction. Finally, we observed rapid clearance of *Co-Npc1* mRNA, indicating that improving transcript stability will be an important area for future optimization (Figure 2A). This may include alternative RNA architectures such as circular or self-amplifying mRNA platforms.[59] Collectively, these findings support LNP-delivered mRNA as a flexible platform for treating the peripheral manifestations of Niemann-Pick type C1 and potentially other lysosomal storage disorders with significant hepatic involvement, while highlighting clear pathways for further optimization toward full systemic and neurological disease correction.

MATERIALS AND METHODS

Reagents are listed in Table S5.

mRNA synthesis

mRNA was obtained from TriLink Biotechnologies as a modified transcript with full substitution of N1-methyl-pseudo-U, capped using CleanCap AG, and polyadenylated with 120 A's. mRNA was then DNase-treated and purified with silica membrane purification. Codon-optimized *Npc1* mRNA sequence is included in Figure S12.

mRNA stability

Bioanalyzer analysis

The quality of TriLink *Npc1* mRNA was assessed by examining RNA size distribution using RNA 6000 Nano Chips (Agilent Technologies, CA, USA) following the manufacturer's instructions. Briefly, 5 μL of RNA marker was added to all sample wells of the RNA 6000 Nano Chip, and 1 μL of either ladder or sample was added to the designated wells. The chip was then vortexed horizontally for 1 min at 2,400 rpm using an IKA vortexer, followed by processing on the Agilent 2100 Bioanalyzer using the total RNA electrophoresis program within 5 min.

Ion-pair reversed-phase liquid chromatography

The liquid chromatography (LC)-UV chromatogram for the immunoprecipitate (IP)-reversed phase (RP) separation of intact mRNA was recorded at a wavelength of 260 nm. Around 2 μg of mRNA sample was injected onto a DNAPac RP column (4 μm particles, 2.1 × 100 mm, Thermo Fisher Scientific), connected to an Acquity UPLC (Waters). The column was maintained at 65°C and operated for 7 min at a flow rate of 0.35 mL/min. The mobile phase consisted of 50 mM dibutyl ammonium acetate (DBAA) and 100 mM triethylammonium acetate (TEA) in water for Mobile Phase A, and 50 mM DBAA and 100 mM TEA in 50% acetonitrile (ACN) for Mobile Phase B. The gradient increased the percentage of Mobile Phase B from 30% to 70% over 5 min, followed by a return to 30% over the next 30 s.

Lipid particle synthesis

LNP preparation

Ionizable amino-lipid (SM-102), helper lipid (DSPC), cholesterol, and DMGPEG2k were dissolved in ethanol at 50:10:38.5:1.5 molar ratio. Each mRNA *GFP* mRNA, *Npc1* mRNA, and codon-optimized *Npc1* mRNA were prepared in 25 mM sodium acetate buffer (pH 4.0). The lipid solution and the mRNA solution were mixed at a 3:1 volume ratio with a total flow rate of 4 mL/min to achieve an N:P ratio (molar ratio of amine groups on SM-102 to phosphates on the mRNA) of 6:1 using the NanoGenerator Flex-S (Precigenome, CA, USA). The resultant LNPs were buffer exchanged into pH 7.4 1× PBS using Amicon 10 kDa molecular weight cut off centrifugal tubes (MilliporeSigma, MA, USA). The EE percent and concentration of mRNA were measured by Quant-iT Ribogreen RNA Assay kit (Invitrogen, Thermo Fisher Scientific, MA, USA) according to manufacturer's instructions. The particle size and PDI of LNPs were measured by DLS using a Zetasizer NanoZS (Malvern Panalytical Ltd., Worcestershire, UK) at a back scattering angle of 173°. Measurements were performed in triplicates for each sample, and the results were analyzed using the built-in software (Zetasizer Software v.7.11). The LNPs were then filtered through a 0.22 μm syringe filter before use.

Cell culture and lipid particle dosing

NPC1$^{+/+}$ or CRISPR-generated *NPC1*$^{-/-}$ HeLa cells were previously described.[60,61] Cells were maintained in DMEM (Cytiva, SH30023.01) media 10% FBS (Bio-Techne R&D systems S11150), 1% penicillin-streptomycin-glutamine (PSG) (Gibco 10378016) at 37°C with 5% CO_2. Cells were treated with 0.15 ng/μL LNP containing mRNA. Cell survival after treatment with lipid particles was measured with a CellTiter 96 Aqueous One Solution Cell Proliferation Assay (Promega G3582).

Western blot

Cultured mammalian cells

HeLa cells were lifted from cell culture plates by washing with PBS and scraping the cell monolayer off the plate with a cell scraper. Cells were lysed in RIPA (radio-immunoprecipitation assay) buffer and protein concentration was measured via dendritic cell (DC)-protein assay (Bio-Rad), then 40 μg protein was loaded onto a NuPAGE 4%–12% gradient Bis-Tris gel (Invitrogen WG1401BX10), blotted for protein, and analyzed via iBright software.[28] All blots were normalized to loading controls, actin or vinculin. Endoglycosidase H assays were performed as described in Azaria et al.[28]

Mouse tissue

At 48 h after injection, mice were perfused with saline and liver tissues were collected and flash frozen. Tissues were homogenized by a tissue homogenizer followed by sonication. The resulting solution was centrifuged at 3,000 g for 5 min at 4°C. Protein concentrations were calculated using the DC-protein assay. A total of 50 μg protein was loaded per well into NuPAGE 4%–12% gradient Bis-Tris gels, then blotted for protein and analyzed via iBright software as described in Azaria et al.[28] and Endoglycosidase H assays were performed.[28]

Immunocytochemistry

Cells were plated into a four-chamber slide and washed in 0.02% saponin in PBS (wash solution), then fixed in 4% PFA (paraformaldehyde) for 20 min at repetition time (RT). Cells were washed and then perforated with a 5-min at −20°C methanol incubation. Next, cells were permeabilized in 0.2% saponin in MiliQ H_2O for 10 min at RT, then blocked for 1 h (blocking buffer: 10% goat serum, 0.02% saponin, 1% BSA in PBS) at RT before primary antibody was added and incubated overnight at 4°C. After washing, cells were incubated in secondary antibody dissolved in blocking solution (secondary blocking solution: 5% goat serum, 1% BSA in PBS) for 1 h at RT.

Filipin staining

HeLa cells were stained with filipin and lectin wheat germ agglutinin (WGA) to visualize plasma membrane as previously described.[29] Slides were imaged on a Zeiss LSM710 confocal microscope.

Cholesterol and cholesteryl ester analysis

Lipid extraction for LC-MS

Cell pellets were resuspended in 1× PBS buffer and were lysed by probe sonication (Amplitude 30%, 1 s on, 1 s off, and 30 s of sonication). The total protein content of the lysed samples was determined using the Pierce BCA Protein Assay (Thermo Scientific). To an equivalent of 50 μg of protein from each sample, 1× PBS was added to the final volume of 40 μL followed by 10 μL of the internal standard mixture containing cholesterol-d7 (Cayman Chemical) 10 nmol and 16:0 cholesteryl-d7 ester (Avanti polar lipids) 3 nmol. To each sample, 800 μL of hexane: isopropyl alcohol (3:2, v/v) was added and vortexed. Samples were then incubated for 20 min on ice, including vortex rounds every 5 min. After the incubation time, 160 μL of 1× PBS was added and vortexed, followed by centrifugation at 1,500 rpm for 5 min at room temperature to induce phase separation. The upper organic phase was transferred into a fresh tube and dried under vacuum. Dried samples were resuspended in 50 μL of methanol/$CHCl_3$ (3:1, v/v).

LC-MS analysis

An Agilent 1290 Infinity II Bio UHPLC system outfitted with an Agilent Eclipse Plus C18 RRHD column (3 × 50 mm, 1.8 μm) was used for the lipid separation. Mobile phase solvents consisted of (A) water with 5 mM ammonium formate +0.1% formic acid and (B) isopropanol: ACN (1:1, v/v) with 5 mM ammonium formate -+0.1% formic acid. Mobile phase solvent flow rate was set to 400 μL/min while the column was maintained under 50°C in a column compartment. The chromatographic gradient is as follows: 75% B at 0–1 min, 75% B to 90% B from 1 to 4 min, 90% B at 4–5 min, 97% B at 5.2–11 min, 100% B at 11.5–15.5 min, and 75% B at 16 min. A post-column equilibration time of 3 min was used for all runs. Electrospray ionization (ESI) source parameters were as follows: gas temp (220°C), drying gas (12 L/min), nebulizer (35 psi), sheath gas temp (220°C), sheath gas flow (11 L/min), capillary voltage (3,500 V), and nozzle voltage (0 V). Data acquisition was performed using an Agilent 6495D triple quadrupole mass spectrometer in positive ion dynamic multiple reaction monitoring (dMRM) mode. Multiple reaction monitoring (MRM) transitions and optimized collision energies are listed in Table S6.

Data analysis

Agilent MassHunter Workstation Quantitative Analysis for QQQ (v.12.1, Build 12.1.938.3) was used to extract the peak areas for each MRM transition. The peak areas of cholesterol and cholesteryl esters were normalized using the cholesterol-d7 and the 16:0 cholesteryl-d7 ester internal standards, respectively. Generation of bar charts and statistical analysis was conducted using Prism GraphPad v.9.4.1. (https://www.graphpad.com/).

Flow cytometry

Cells were incubated in trypsin for 5 min, then centrifuged at 1,000 g for 5 min to form a pellet. Cells were resuspended in 500 μL solution (1% FBS in −/− PBS) and strained with a 70 μm strainer for flow analysis. Cells were analyzed for fluorescent intensity via an LSR flow cytometer with UV from the University of Iowa Flow Cytometry Facility. Gates were set for fetal calf serum (FSC)-A vs. saline sodium citrate (SSC)-A, FSC-A vs. FSC-W, and FSC-A vs. GFP-A to measure the levels of GFP intensity.

Mice

Npc1$^{-/-}$ BALB/cJ mice were obtained from Jackson Laboratory (#003092) and backcrossed to C57BL6/J for at least 10 generations. Mixes of both male and female mice were used for all experiments. Backcrossing BALB/cJ and C57BL6/J mice allows for hybrid vigor, or heterosis, in which improved health of the homozygous offspring allows for improved health and higher likelihood of Mendelian ratios after breeding due to the masking of other deleterious alleles. This breeding scheme enables Mendelian ratios in the progeny F1 hybrid mice and was described previously.[62] Serum liver function tests were performed by the University of Michigan In-Vivo Animal Core (IVAC). We have chosen to limit use of animals and therefore only include *GFP*:LNP-treated groups for reference. *GFP*:LNP groups control for the biological effects of cellular LNP uptake, immune response, and lipid exposure. Accordingly, results are limited to comparisons among LNP-treated groups, but we do not infer differences in results with injection-naïve animals. All animal procedures were approved by the University of Michigan Committee on the Use and Care of Animals (PRO00011667) and the University of Iowa (3042533) animal care and use committees. Animal experiments were conducted in accordance with both institutional and federal guidelines.

Liver function tests

Whole blood was collected into serum separator tubes, allowed to clot, and separated into serum by centrifugation. Serum chemistries were run on a Beckman Coulter AU480 (Beckman Coulter, Brea, CA, USA) automated clinical chemistry analyzer. Analysis was performed for the liver enzymes AST, ALT, TBIL, and ALKP. Assays were performed within the Unit for Laboratory Animal Medicine In Vivo Animal Core pathology laboratory at the University of Michigan. Quality control was performed daily using manufacturer-provided reagents, and the laboratory is a participant in an external independent quarterly quality assurance program (Veterinary Laboratory Association Quality Assurance Program).

RT-qPCR

Cultured mammalian cells

RNA was isolated using TRIzol Reagent (Ambion 15596018/Invitrogen 15596026), following manufacturer's instructions. RNA was converted to cDNA using the High-Capacity Reverse Transcription kit (Applied Biosystems 4368814). qPCR was conducted using 10 ng cDNA and TaqMan probes (Integrated DNA Technologies, IDT) for *Npc1* mRNA and codon-optimized *Npc1* mRNA. qPCR was performed using custom probes from IDT. Quantitative reverse-transcription (RT-qPCR) was performed using StepOnePlus Real-Time PCR Systems and relative expression was calculated by the 2 ^ (−delta delta Ct).

Mouse tissue

RNA was isolated using TRIzol Reagent (Ambion 15596018), following manufacturer's instructions. RNA was converted to cDNA using the High-Capacity Reverse Transcription kit (Applied Biosystems 4368814). RT-qPCR using 10 ng cDNA and TaqMan probes (Thermo Fisher) for *Hmgcs1* (Mm01304569_m1). RT-qPCR was performed using ABI 7900HT Sequence Detection System and relative expression was calculated by the 2 ^ (−delta delta Ct) method using Sequence Detection Systems software.

Lipidomics

Lipid extraction for LC-MS

Liver tissues were homogenized in 1× PBS buffer with phosphatase inhibitors (1 mM NaF, 1 mM β-Glycerophosphate, 1 mM PMSF, 1 mM Na_3VO_4) using a vortex homogenizer. Homogenized tissues were further lysed by probe sonication (Amplitude 40%, 1 s on, 1 s off, 30 s of sonication). The total protein content of the lysed samples was determined using the Pierce BCA Protein Assay (Thermo Scientific). To an equivalent of 300 μg of protein from each sample, 1× PBS was added to the final volume of 40 μL followed by 15 μL of the internal standard mixture containing cholesterol-d7 (Cayman chemical) 15 nmol and 16:0 cholesteryl-d7 ester (Avanti polar lipids) 4.5 nmol. To each sample, 800 μL of hexane: isopropyl alcohol (3:2, v/v) was added and vortexed. Samples were then incubated for 20 min on ice, including vortex rounds every 5 min. After the incubation time, 160 μL of 1× PBS was added and vortexed, followed by centrifugation at 1,500 rpm for 5 min at room temperature to induce phase separation. The upper organic phase was transferred into a fresh tube and dried under vacuum. Dried samples were resuspended in 100 μL of methanol/CHCl3 (3:1, v/v).

LC-MS analysis

An Agilent 1260 HPLC system outfitted with an Agilent Eclipse Plus C18 RRHD column (3 × 50 mm, 1.8 μm) was used for the lipid separation. Mobile phase solvents consisted of (A) water with 5 mM ammonium formate +0.1% formic acid and (B) isopropanol: ACN (1:1, v/v) with 5 mM ammonium formate +0.1% formic acid. Mobile phase solvent flow rate was set to 400 μL per minute while the column was maintained under 50°C in a column compartment. The chromatographic gradient is as follows: 75% B at 0–1 min, 75% B to 90% B from 1 to 4 min, 90% B at 4–8 min, 100% B at 8.2–20 min, 75% B at 20.5 min. A post-column equilibration time of 5 min was used for all runs. ESI source parameters were as follows: gas temp (220°C), drying gas (12 L/min), nebulizer (35 psi), sheath gas temp (220°C), sheath gas flow (11 L/min), VCap (3,000 V), and fragmentor (175 V). Data acquisition was performed using an Agilent 6550 quadrupole time-of-flight (Q-TOF) mass spectrometer in positive ion mode m/z range 200–1,700 in MS-only mode. A pooled sample of equal amounts of all biological samples was analyzed using the targeted MS/MS mode with a fixed collision energy of 25 eV.

Data analysis

Agilent Profinder software was used to integrate the extracted ion chromatograms for the lipid molecules. The peak areas of cholesterol and cholesteryl esters were normalized using the cholesterol-d7 and the 16:0 cholesteryl-d7 ester internal standards, respectively. Generation of bar charts and statistical analysis was conducted using Prism GraphPad v.9.4.1. (https://www.graphpad.com/).

Statistical analysis

Statistical tests were performed in GraphPad Prism (v.9.4.1). Multiple comparisons were analyzed by *t* test, 1-way, or 2-way ANOVA with Tukey's post hoc multiple comparisons test or Bonferroni correction, respectively. *p* values of less than or equal to 0.05 were considered significant.

Bulk RNA-seq

RNA was isolated using TRIzol Reagent (Ambion 15596018), according to the manufacturer's instructions, and 1 μg of total RNA was used for the construction of sequence libraries. RNA libraries for RNA-seq were prepared by the Advanced Genomics Core at the University of Michigan with the manufacturer's protocol for Poly-A enrichment. This pool was subjected to 151 bp paired-end sequencing according to the manufacturer's protocol (Illumina Nova-Seq). BCL Convert Conversion Software v.4.3.6 (Illumina) was used to generate de-multiplexed Fastq files. The raw reads were trimmed with Cutadapt v.4.8 and the quality of the trimmed reads were evaluated with FastQC v.0.11.8 while Fastq Screen v.0.15.3 was used to screen for various types of contamination (https://doi.org/10.14806/ej.17.1.200, https://www.bioinformatics.babraham.ac.uk/projects/fastqc/). The trimmed reads were then mapped to the mouse reference genome (GRCm38) using the STAR aligner v.2.7.9a, and the quality of the alignments were evaluated with MultiQC v.1.12.[63,64]

A STAR genome index was built prior to alignment using the reference genome FASTA file and the corresponding Ensembl v.102 GTF annotation file.[64,65] The reads were aligned with default STAR parameters, and the resulting alignment files were generated in BAM (binary alignment map) format. Aligned reads were quantified at the gene level using featureCounts v.2.0.1 with the same GTF file used for alignment, and the resulting raw count matrix was used for downstream analysis.[66] Genes that had less than ten counts across all samples were removed. DE analysis was performed in R with the DESeq2 package v.1.30.1, to conduct three separate comparisons: (1) *GFP*:LNP-treated *Npc1*$^{-/-}$ vs. no treatment *Npc1*$^{+/+}$ mice, (2) *Co-Npc1*:LNP-treated *Npc1*$^{-/-}$ vs. *GFP*:LNP-treated *Npc1*$^{-/-}$ mice, and (3) *Co-Npc1*:LNP-treated *Npc1*$^{-/-}$ vs. no treatment *Npc1*$^{+/+}$ mice (https://www.r-project.org/).[67] Genes for each comparison were considered differentially expressed if they had an adjusted *p* value of less than 0.05. DE analysis revealed 873 DEGs for *Co-Npc1*:LNP-treated *Npc1*$^{-/-}$ vs. *GFP*:LNP-treated *Npc1*$^{-/-}$ mice, 8,030 DEGs for GFP:LNP-treated *Npc1*$^{-/-}$ vs. saline-treated *Npc1*$^{+/+}$ *mice*, and 5,255 DEGs for *Co-Npc1*:LNP-treated *Npc1*$^{-/-}$ vs. saline-treated *Npc1*$^{+/+}$ mice (adj. *p* value <0.05).

The set of DEGs from the comparison of *Co-Npc1*:LNP-treated *Npc1*$^{-/-}$ vs. *GFP*:LNP-treated *Npc1*$^{-/-}$ mice were retained if they were DE in either of the other two comparisons and used as the input gene set for the MENTOR algorithm.[40] This filtered set of DEGs were then mapped to their human HGNC gene symbol orthologs.[68] A multiplex containing 22 layers was constructed with the RWRToolkit package in R, and was used with the input gene list for MENTOR to identify modules of functionally related genes.[69] The multiplex network consisted of six layers from the HumanNet v.3 project, and 16 cell type-specific predictive expression network layers created with iRF-LOOP (iterative random forest leave one out prediction) from publicly available GTEx human single cell liver data.[70–72] The MENTOR input gene set was also filtered to only retain genes present in at least one layer of the multiplex network. Statistical significance of MENTOR modules was assessed using a permutation-based framework. Modules were grouped by size (number of genes), and for each size group, 10,000 random gene sets of matched size were sampled from the multiplex network to construct null distributions of mean pairwise dissimilarity. For each MENTOR module, the observed mean within-modules dissimilarity was compared to the corresponding null distribution to compute an empirical *p* value. *p* values were adjusted for multiple testing with the Benjamini-Hochberg procedure across all modules.[73] The MENTOR-IA algorithm was employed to elucidate the biological functions implicated within each MENTOR module.[41]

SnRNA-seq

Livers were collected from 7-weeks-old mice, 3 *Npc1*$^{+/+}$ and 3 *Npc1*$^{-/-}$ following isoflurane euthanasia and decapitation. Liver tissue was dissected, flash frozen in liquid nitrogen, and homogenized with a glass Dounce tissue grinder (25× with pestle A and 25× with pestle B) in 2 mL of EZ PREP buffer (Sigma NUC-101). Homogenized liver tissue was incubated on ice for 5 min, followed by an addition of 2 mL of EZ PREP, then centrifuged at 500 g for 5 min at 4°C to pellet nuclei. Resulting nuclei were washed with 4 mL EZ PREP and incubated for 5 min on ice then centrifuged using the same parameters. Samples were washed with 4 mL nuclear suspension buffer (0.01% BSA, RNase inhibitor [Takara 2313 A]) and centrifuged using the same parameters. About 1 mL of nuclear suspension buffer was added to the pellet for resuspension, then filtered with a 35 μM cell strainer (Corning 352235) and counted. Nuclei were diluted to 800–1,500 cells/μL and libraries were generated with the 10× Chromium Controller system. Single-nucleus libraries were sequenced using the Illumina NovaSeq 6000. Alignment, filtering, ALRA (adaptive-threshold low-rank approximation), expression value calculation, and DE analysis were applied and measured according to Kunkel et al.[62]

Fastq files for each sample were aligned with STARSolo v.2.7.9a using the mouse reference genome (GRCm38).[64] Low quality cells were filtered using the STARSolo EmptyDrops methodology with default parameters.[74] Mitochondrial content was calculated as the percentage of total reads within each cell mapping to mitochondrial-encoded genes. Cells exhibiting mitochondrial gene expression greater than 0.10 were removed. Additional quality control filtering was applied to remove potential doublets. Specifically, a conservative global cutoff of 25,000 unique molecular identifiers (UMIs) was applied to remove high-count outlier cells across samples. Genes expressing a total of ten or less UMI counts across all cells were removed. Density distributions of total UMI and total unique gene counts were constructed with gggplot2 across samples to confirm

quality control filters.[75] Following filtering, nuclei were log-normalized with a default scale factor of 10,000 using Seurat. Imputation was performed on the normalized expression matrix using the adaptively thresholded low-rank approximation algorithm (ALRA) to impute dropout events.[76] The following tasks were completed using the Seurat toolkit.[77–81] For integration, Seurat objects per sample ID were normalized independently and the top 3,000 variable features were identified.[77–81] Integration anchors were computed using reciprocal principal component analysis (PCA) with 2,000 shared features, and integration was performed with FindIntegrationAnchors and IntegrateData functions in Seurat. Following integration, expression values for each gene were centered and scaled with the ScaleData function in Seurat. A shared nearest neighbor (SNN) graph was constructed using the top 50 principal components, and clustering was performed across a range of resolution parameters using the Seurat FindClusters function. Final clustering resolution was selected based on marker gene expression patterns and to identify broad cell type populations. Additional dimensionality reduction was performed using RunUMAP in Seurat with varying parameters for minimum distance and number of neighbors.

DE analysis was performed for marker gene identification across clusters using FindAllMarkers in Seurat, restricting analysis to variable features and curated marker gene sets, and filtering results using an adjusted p value threshold of 0.01 and absolute $\log_2$ fold-change threshold of 0.25. The Bonferroni method for multiple comparisons p value adjustment was utilized for all DE analyses.[82] A dot plot was generated with ggplot2 to visualize the expression of canonical liver cell type marker genes for each cell cluster.[75] Cell type labels were manually assigned to each cluster based on the results of the DE analysis and inspection of the dot plot visualization. Clusters were annotated using canonical liver cell type markers, resulting in identification of Chol/HPC, Endos, HSCs, Hep, MNPs, and Myos (Table S3).[42–50] Following cell type assignment, the FindMarkers function in Seurat was used to perform DE analysis between *Npc1*$^{-/-}$ and wild type (WT) livers for each cell type cluster independently, and DEGs were identified with an adjusted p value <0.01 and $|\log_2(\text{FC})| \geq 0.25$.[77–81]

DATA AND CODE AVAILABILITY

Data and supporting the findings of these studies are available from the corresponding authors upon reasonable request. The raw bulk RNA-seq and snRNA-seq data have been deposited in the GEO database under the series accession codes: GEO: GSE335560 and GEO: GSE335561, respectively.

ACKNOWLEDGMENTS

This work was funded by Life for Liam and Friends (to M.L.S.), Firefly Fund (to M.L.S.), NIH (R35GM154959 to M.L.S., T32 GM 145441 to E.R.K.; R01 NS122746 to A.P.L.). This research used resources of the Oak Ridge Leadership Computing Facility at the Oak Ridge National Laboratory, which is supported by the Office of Science of the U.S. Department of Energy under contract no. DE-AC05-00OR22725. Multiplex network methods development was supported by NIH grants DA051908 (DAJ, KAS, AT), DA051913 (DAJ, KAS, AT), and DA054071 (DAJ, KAS, AT). The manuscript was coauthored by UT-Battelle, LLC under Contract No. DE-AC05-00OR22725 with the US Department of Energy. The US Government retains and the publisher, by accepting the article for publication, acknowledges that the US Government retains a nonexclusive, paid-up, irrevocable, worldwide license to publish or reproduce the published form of this manuscript, or allow others to do so, for US Government purposes. The authors would like to acknowledge use of the Small Animal Imaging Core Facility, a core resource supported by the Department of Radiology at The University of Iowa with the IVIS purchased through the S10 award 1S10OD026835. The authors would also like to acknowledge use of the University of Iowa Central Microscopy Research Facility, a core resource supported by the University of Iowa Vice President for Research, and the Carver College of Medicine. Graphical abstract was created in BioRender. Koufer, E. (2026) https://BioRender.com/bghza3k.

AUTHOR CONTRIBUTIONS

E.R.K. designed and performed experiments, analyzed data, and wrote the manuscript; A.T. analyzed data and wrote the manuscript; Y.N., A.B.C., N.K.L., and K.C.P. designed experiments, analyzed data, and edited the manuscript; K.A.S. edited the manuscript; T.J.K., T.H., E.S.P., and M.L.L. performed experiments and analyzed data; J.S., O.I., S.M.C., A.P.L., D.A.J., A.S.S., M.L.S. designed experiments, analyzed data, and edited the manuscript.

DECLARATION OF INTERESTS

A US 596 A patent has been submitted based on this work: US application no. 63/656,343, filed on June 5, 2024.

SUPPLEMENTAL INFORMATION

Supplemental information can be found online at https://doi.org/10.1016/j.omtn.2026.103022.

REFERENCES

1. Vanier, M.T. (2010). Niemann-Pick disease type C. Orphanet J. Rare Dis. *5*, 16. https://doi.org/10.1186/1750-1172-5-16.
2. Peake, K.B., and Vance, J.E. (2010). Defective cholesterol trafficking in Niemann-Pick C-deficient cells. FEBS Lett. *584*, 2731–2739. https://doi.org/10.1016/j.febslet.2010.04.047.
3. Las Heras, M., Szenfeld, B., Ballout, R.A., Buratti, E., Zanlungo, S., Dardis, A., and Klein, A.D. (2023). Understanding the phenotypic variability in Niemann-Pick disease type C (NPC): a need for precision medicine. npj Genom. Med. *8*, 21. https://doi.org/10.1038/s41525-023-00365-w.
4. Kumagai, T., Terashima, H., Uchida, H., Fukuda, A., Kasahara, M., Kosuga, M., Okuyama, T., Tsunoda, T., Inui, A., Fujisawa, T., et al. (2019). A case of Niemann-Pick disease type C with neonatal liver failure initially diagnosed as neonatal hemochromatosis. Brain Dev. *41*, 460–464. https://doi.org/10.1016/j.braindev.2019.01.004.
5. Evans, W.R.H., Nicoli, E.R., Wang, R.Y., Movsesyan, N., and Platt, F.M. (2017). Case Report: Ursodeoxycholic acid treatment in Niemann-Pick disease type C; clinical experience in four cases. Wellcome Open Res. *2*, 75. https://doi.org/10.12688/wellcomeopenres.11854.1.
6. Hegarty, R., Deheragoda, M., Fitzpatrick, E., and Dhawan, A. (2018). Paediatric fatty liver disease (PeFLD): All is not NAFLD - Pathophysiological insights and approach to management. J. Hepatol. *68*, 1286–1299. https://doi.org/10.1016/j.jhep.2018.02.006.
7. Pentchev, P.G., Comly, M.E., Kruth, H.S., Vanier, M.T., Wenger, D.A., Patel, S., and Brady, R.O. (1985). A defect in cholesterol esterification in Niemann-Pick disease (type C) patients. Proc. Natl. Acad. Sci. USA *82*, 8247–8251. https://doi.org/10.1073/pnas.82.23.8247.
8. Carstea, E.D., Morris, J.A., Coleman, K.G., Loftus, S.K., Zhang, D., Cummings, C., Gu, J., Rosenfeld, M.A., Pavan, W.J., Krizman, D.B., et al. (1997). Niemann-Pick C1 disease gene: homology to mediators of cholesterol homeostasis. Science *277*, 228–231. https://doi.org/10.1126/science.277.5323.228.
9. Schultz, M.L., Krus, K.L., and Lieberman, A.P. (2016). Lysosome and endoplasmic reticulum quality control pathways in Niemann-Pick type C disease. Brain Res. *1649*, 181–188. https://doi.org/10.1016/j.brainres.2016.03.035.
10. Furtado, D., Cortez-Jugo, C., Hung, Y.H., Bush, A.I., and Caruso, F. (2022). mRNA Treatment Rescues Niemann-Pick Disease Type C1 in Patient Fibroblasts. Mol. Pharm. *19*, 3987–3999. https://doi.org/10.1021/acs.molpharmaceut.2c00463.

11. Hou, X., Zaks, T., Langer, R., and Dong, Y. (2021). Lipid nanoparticles for mRNA delivery. Nat. Rev. Mater. *6*, 1078–1094. https://doi.org/10.1038/s41578-021-00358-0.

12. Monfrini, E., Baso, G., Ronchi, D., Meneri, M., Gagliardi, D., Quetti, L., Verde, F., Ticozzi, N., Ratti, A., Di Fonzo, A., et al. (2024). Unleashing the potential of mRNA therapeutics for inherited neurological diseases. Brain *147*, 2934–2945. https://doi.org/10.1093/brain/awae135.

13. Rohner, E., Yang, R., Foo, K.S., Goedel, A., and Chien, K.R. (2022). Unlocking the promise of mRNA therapeutics. Nat. Biotechnol. *40*, 1586–1600. https://doi.org/10.1038/s41587-022-01491-z.

14. Martini, P.G.V., and Guey, L.T. (2019). A New Era for Rare Genetic Diseases: Messenger RNA Therapy. Hum. Gene Ther. *30*, 1180–1189. https://doi.org/10.1089/hum.2019.090.

15. Qin, S., Tang, X., Chen, Y., Chen, K., Fan, N., Xiao, W., Zheng, Q., Li, G., Teng, Y., Wu, M., and Song, X. (2022). mRNA-based therapeutics: powerful and versatile tools to combat diseases. Signal Transduct. Targeted Ther. *7*, 166. https://doi.org/10.1038/s41392-022-01007-w.

16. Zeng, C., Zhang, C., Walker, P.G., and Dong, Y. (2022). Formulation and Delivery Technologies for mRNA Vaccines. Curr. Top. Microbiol. Immunol. *440*, 71–110. https://doi.org/10.1007/82_2020_217.

17. Huang, X., Ma, Y., Ma, G., and Xia, Y. (2024). Unlocking the Therapeutic Applicability of LNP-mRNA: Chemistry, Formulation, and Clinical Strategies. Research *7*, 370. https://doi.org/10.34133/research.0370.

18. Trepotec, Z., Lichtenegger, E., Plank, C., Aneja, M.K., and Rudolph, C. (2019). Delivery of mRNA Therapeutics for the Treatment of Hepatic Diseases. Mol. Ther. *27*, 794–802. https://doi.org/10.1016/j.ymthe.2018.12.012.

19. Cacicedo, M.L., Limeres, M.J., and Gehring, S. (2022). mRNA-Based Approaches to Treating Liver Diseases. Cells *11*, 3328. https://doi.org/10.3390/cells11203328.

20. Harvie, P., Wong, F.M., and Bally, M.B. (2000). Use of poly(ethylene glycol)-lipid conjugates to regulate the surface attributes and transfection activity of lipid-DNA particles. J. Pharmaceut. Sci. *89*, 652–663. https://doi.org/10.1002/(SICI)1520-6017(200005)89:5<652::AID-JPS11>3.0.CO;2-H.

21. Li, B., Raji, I.O., Gordon, A.G.R., Sun, L., Raimondo, T.M., Oladimeji, F.A., Jiang, A.Y., Varley, A., Langer, R.S., and Anderson, D.G. (2024). Accelerating ionizable lipid discovery for mRNA delivery using machine learning and combinatorial chemistry. Nat. Mater. *23*, 1002–1008. https://doi.org/10.1038/s41563-024-01867-3.

22. Cheng, X., and Lee, R.J. (2016). The role of helper lipids in lipid nanoparticles (LNPs) designed for oligonucleotide delivery. Adv. Drug Deliv. Rev. *99*, 129–137. https://doi.org/10.1016/j.addr.2016.01.022.

23. Zhang, R., El-Mayta, R., Murdoch, T.J., Warzecha, C.C., Billingsley, M.M., Shepherd, S.J., Gong, N., Wang, L., Wilson, J.M., Lee, D., and Mitchell, M.J. (2021). Helper lipid structure influences protein adsorption and delivery of lipid nanoparticles to spleen and liver. Biomater. Sci. *9*, 1449–1463. https://doi.org/10.1039/d0bm01609h.

24. Mulroney, T.E., Pöyry, T., Yam-Puc, J.C., Rust, M., Harvey, R.F., Kalmar, L., Horner, E., Booth, L., Ferreira, A.P., Stoneley, M., et al. (2024). N(1)-methylpseudouridylation of mRNA causes +1 ribosomal frameshifting. Nature *625*, 189–194. https://doi.org/10.1038/s41586-023-06800-3.

25. Horstick, E.J., Jordan, D.C., Bergeron, S.A., Tabor, K.M., Serpe, M., Feldman, B., and Burgess, H.A. (2015). Increased functional protein expression using nucleotide sequence features enriched in highly expressed genes in zebrafish. Nucleic Acids Res. *43*, e48. https://doi.org/10.1093/nar/gkv035.

26. Presnyak, V., Alhusaini, N., Chen, Y.H., Martin, S., Morris, N., Kline, N., Olson, S., Weinberg, D., Baker, K.E., Graveley, B.R., and Coller, J. (2015). Codon optimality is a major determinant of mRNA stability. Cell *160*, 1111–1124. https://doi.org/10.1016/j.cell.2015.02.029.

27. Gustafsson, C., Govindarajan, S., and Minshull, J. (2004). Codon bias and heterologous protein expression. Trends Biotechnol. *22*, 346–353. https://doi.org/10.1016/j.tibtech.2004.04.006.

28. Azaria, R.D., Correia, A.B., Schache, K.J., Zapata, M., Pathmasiri, K.C., Mohanty, V., Nannapaneni, D.T., Ashfeld, B.L., Helquist, P., Wiest, O., et al. (2024). Mutant induced neurons and humanized mice enable identification of Niemann-Pick type C1 proteostatic therapies. JCI Insight *9*, e179525. https://doi.org/10.1172/jci.insight.179525.

29. Schultz, M.L., Schache, K.J., Azaria, R.D., Kuiper, E.Q., Erwood, S., Ivakine, E.A., Farhat, N.Y., Porter, F.D., Pathmasiri, K.C., Cologna, S.M., et al. (2022). Species-specific differences in NPC1 protein trafficking govern therapeutic response in Niemann-Pick type C disease. JCI Insight *7*, e160308. https://doi.org/10.1172/jci.insight.160308.

30. Brodsky, J.L., and Skach, W.R. (2011). Protein folding and quality control in the endoplasmic reticulum: Recent lessons from yeast and mammalian cell systems. Curr. Opin. Cell Biol. *23*, 464–475. https://doi.org/10.1016/j.ceb.2011.05.004.

31. Gelsthorpe, M.E., Baumann, N., Millard, E., Gale, S.E., Langmade, S.J., Schaffer, J.E., and Ory, D.S. (2008). Niemann-Pick type C1 I1061T mutant encodes a functional protein that is selected for endoplasmic reticulum-associated degradation due to protein misfolding. J. Biol. Chem. *283*, 8229–8236. https://doi.org/10.1074/jbc.M708735200.

32. Lieberman, A.P., Puertollano, R., Raben, N., Slaugenhaupt, S., Walkley, S.U., and Ballabio, A. (2012). Autophagy in lysosomal storage disorders. Autophagy *8*, 719–730. https://doi.org/10.4161/auto.19469.

33. Sarkar, S., Carroll, B., Buganim, Y., Maetzel, D., Ng, A.H.M., Cassady, J.P., Cohen, M.A., Chakraborty, S., Wang, H., Spooner, E., et al. (2013). Impaired autophagy in the lipid-storage disorder Niemann-Pick type C1 disease. Cell Rep. *5*, 1302–1315. https://doi.org/10.1016/j.celrep.2013.10.042.

34. Kabeya, Y., Mizushima, N., Ueno, T., Yamamoto, A., Kirisako, T., Noda, T., Kominami, E., Ohsumi, Y., and Yoshimori, T. (2000). LC3, a mammalian homologue of yeast Apg8p, is localized in autophagosome membranes after processing. EMBO J. *19*, 5720–5728. https://doi.org/10.1093/emboj/19.21.5720.

35. Tanida, I., Ueno, T., and Kominami, E. (2004). LC3 conjugation system in mammalian autophagy. Int. J. Biochem. Cell Biol. *36*, 2503–2518. https://doi.org/10.1016/j.biocel.2004.05.009.

36. Wang, C., Scott, S.M., Subramanian, K., Loguercio, S., Zhao, P., Hutt, D.M., Farhat, N.Y., Porter, F.D., and Balch, W.E. (2019). Quantitating the epigenetic transformation contributing to cholesterol homeostasis using Gaussian process. Nat. Commun. *10*, 5052. https://doi.org/10.1038/s41467-019-12969-x.

37. Maxfield, F.R., and Wüstner, D. (2012). Analysis of cholesterol trafficking with fluorescent probes. Methods Cell Biol. *108*, 367–393. https://doi.org/10.1016/B978-0-12-386487-1.00017-1.

38. Sayre, N.L., Rimkunas, V.M., Graham, M.J., Crooke, R.M., and Liscum, L. (2010). Recovery from liver disease in a Niemann-Pick type C mouse model. J. Lipid Res. *51*, 2372–2383. https://doi.org/10.1194/jlr.M007211.

39. Schultz, M.L., Fawaz, M.V., Azaria, R.D., Hollon, T.C., Liu, E.A., Kunkel, T.J., Halseth, T.A., Krus, K.L., Ming, R., Morin, E.E., et al. (2019). Synthetic high-density lipoprotein nanoparticles for the treatment of Niemann-Pick diseases. BMC Med. *17*, 200. https://doi.org/10.1186/s12916-019-1423-5.

40. Sullivan, K.A., Miller, J.I., Townsend, A., Morgan, M., Lane, M., Pavicic, M., Shah, M., Cashman, M., and Jacobson, D.A. (2024). MENTOR: Multiplex Embedding of Networks for Team-Based Omics Research. Preprint at bioRxiv. https://doi.org/10.1101/2024.07.17.603821.

41. Vlot, A.H., Lane, M., Sullivan, K.A., Kruse, P., Dandy, J., Kaplanoglu, S., Townsend, A., Merlet, J., and Jacobson, D.A. (2025). The MENTOR Interpretation Agent: From Network Embeddings to Mechanistic Narratives via Retrieval-Augmented LLMs. https://linklings.s3.amazonaws.com/organizations/pasc/pasc25/submissions/stype108/qFjMh-pos156s2.pdf.

42. Paris, J., and Henderson, N.C. (2022). Liver zonation, revisited. Hepatology *76*, 1219–1230. https://doi.org/10.1002/hep.32408.

43. Ang, C.H., Arandjelovic, P., Cheng, J., Yang, J., Guo, F., Yu, Y., Nelameham, S., Whitehead, L., Li, J., Silver, D.L., et al. (2025). Self-maintenance of zonal hepatocytes during adult homeostasis and their complex plasticity upon distinct liver injuries. Cell Rep. *44*, 115093. https://doi.org/10.1016/j.celrep.2024.115093.

44. Carlessi, R., Denisenko, E., Boslem, E., Köhn-Gaone, J., Main, N., Abu Bakar, N.D.B., Shirolkar, G.D., Jones, M., Beasley, A.B., Poppe, D., et al. (2023). Single-nucleus RNA sequencing of pre-malignant liver reveals disease-associated hepatocyte state with

HCC prognostic potential. Cell Genom. *3*, 100301. https://doi.org/10.1016/j.xgen.2023.100301.

45. Halpern, K.B., Shenhav, R., Matcovitch-Natan, O., Toth, B., Lemze, D., Golan, M., Massasa, E.E., Baydatch, S., Landen, S., Moor, A.E., et al. (2017). Single-cell spatial reconstruction reveals global division of labour in the mammalian liver. Nature *542*, 352–356. https://doi.org/10.1038/nature21065.

46. Xiao, Y., Batmanov, K., Hu, W., Zhu, K., Tom, A.Y., Guan, D., Jiang, C., Cheng, L., McCright, S.J., Yang, E.C., et al. (2023). Hepatocytes demarcated by EphB2 contribute to the progression of nonalcoholic steatohepatitis. Sci. Transl. Med. *15*, eadc9653. https://doi.org/10.1126/scitranslmed.adc9653.

47. Karri, K., and Waxman, D.J. (2023). TCDD dysregulation of lncRNA expression, liver zonation and intercellular communication across the liver lobule. Toxicol. Appl. Pharmacol. *471*, 116550. https://doi.org/10.1016/j.taap.2023.116550.

48. Goldfarb, C.N., Karri, K., Pyatkov, M., and Waxman, D.J. (2022). Interplay Between GH-regulated, Sex-biased Liver Transcriptome and Hepatic Zonation Revealed by Single-Nucleus RNA Sequencing. Endocrinology *163*, bqac059. https://doi.org/10.1210/endocr/bqac059.

49. Andrews, T.S., Atif, J., Liu, J.C., Perciani, C.T., Ma, X.Z., Thoeni, C., Slyper, M., Eraslan, G., Segerstolpe, A., Manuel, J., et al. (2022). Single-Cell, Single-Nucleus, and Spatial RNA Sequencing of the Human Liver Identifies Cholangiocyte and Mesenchymal Heterogeneity. Hepatol. Commun. *6*, 821–840. https://doi.org/10.1002/hep4.1854.

50. Hu, S., Liu, S., Bian, Y., Poddar, M., Singh, S., Cao, C., McGaughey, J., Bell, A., Blazer, L.L., Adams, J.J., et al. (2022). Single-cell spatial transcriptomics reveals a dynamic control of metabolic zonation and liver regeneration by endothelial cell Wnt2 and Wnt9b. Cell Rep. Med. *3*, 100754. https://doi.org/10.1016/j.xcrm.2022.100754.

51. Yu, W., Gong, J.S., Ko, M., Garver, W.S., Yanagisawa, K., and Michikawa, M. (2005). Altered cholesterol metabolism in Niemann-Pick type C1 mouse brains affects mitochondrial function. J. Biol. Chem. *280*, 11731–11739. https://doi.org/10.1074/jbc.M412898200.

52. Schedin, S., Sindelar, P.J., Pentchev, P., Brunk, U., and Dallner, G. (1997). Peroxisomal impairment in Niemann-Pick type C disease. J. Biol. Chem. *272*, 6245–6251. https://doi.org/10.1074/jbc.272.10.6245.

53. Zhu, X., Yin, L., Theisen, M., Zhuo, J., Siddiqui, S., Levy, B., Presnyak, V., Frassetto, A., Milton, J., Salerno, T., et al. (2019). Systemic mRNA Therapy for the Treatment of Fabry Disease: Preclinical Studies in Wild-Type Mice, Fabry Mouse Model, and Wild-Type Non-human Primates. Am. J. Hum. Genet. *104*, 625–637. https://doi.org/10.1016/j.ajhg.2019.02.003.

54. Bian, X., Guo, Q., Yau, L.F., Yang, L., Wang, X., Zhao, S., Wu, S., Qin, X., Jiang, Z.H., and Li, C. (2025). Berberine-inspired ionizable lipid for self-structure stabilization and brain targeting delivery of nucleic acid therapeutics. Nat. Commun. *16*, 2368. https://doi.org/10.1038/s41467-025-57488-0.

55. Suzuki, Y., Yakuwa, M., Sato, M., Samaridou, E., Beck-Broichsitter, M., Maeki, M., Tokeshi, M., Yamada, Y., Harashima, H., and Sato, Y. (2025). Splenic B cell-targeting lipid nanoparticles for safe and effective mRNA vaccine delivery. J. Contr. Release *382*, 113687. https://doi.org/10.1016/j.jconrel.2025.113687.

56. Sun, H., Zhang, Y., Wang, J., Su, J., Zhou, D., Yu, X., Xu, Y., and Yang, W. (2023). Application of Lung-Targeted Lipid Nanoparticle-delivered mRNA of soluble PD-L1 via SORT Technology in Acute Respiratory Distress Syndrome. Theranostics *13*, 4974–4992. https://doi.org/10.7150/thno.86466.

57. Kawai, A., Shimizu, T., Tanaka, H., Shichinohe, S., Anindita, J., Hirose, M., Kawahara, E., Senpuku, K., Shimooka, M., Quynh Mai, L.T., et al. (2025). Low-inflammatory lipid nanoparticle-based mRNA vaccine elicits protective immunity against H5N1 influenza virus with reduced adverse reactions. Mol. Ther. *33*, 529–547. https://doi.org/10.1016/j.ymthe.2024.12.032.

58. Wang, J., Ding, Y., Chong, K., Cui, M., Cao, Z., Tang, C., Tian, Z., Hu, Y., Zhao, Y., and Jiang, S. (2024). Recent Advances in Lipid Nanoparticles and Their Safety Concerns for mRNA Delivery. Vaccines (Basel) *12*, 1148. https://doi.org/10.3390/vaccines12101148.

59. Chen, R., Wang, S.K., Belk, J.A., Amaya, L., Li, Z., Cardenas, A., Abe, B.T., Chen, C.K., Wender, P.A., and Chang, H.Y. (2023). Author Correction: Engineering circular RNA for enhanced protein production. Nat. Biotechnol. *41*, 293. https://doi.org/10.1038/s41587-022-01472-2.

60. Ilnytska, O., Lai, K., Gorshkov, K., Schultz, M.L., Tran, B.N., Jeziorek, M., Kunkel, T.J., Azaria, R.D., McLoughlin, H.S., Waghalter, M., et al. (2021). Enrichment of NPC1-deficient cells with the lipid LBPA stimulates autophagy, improves lysosomal function, and reduces cholesterol storage. J. Biol. Chem. *297*, 100813. https://doi.org/10.1016/j.jbc.2021.100813.

61. McCauliff, L.A., Langan, A., Li, R., Ilnytska, O., Bose, D., Waghalter, M., Lai, K., Kahn, P.C., and Storch, J. (2019). Intracellular cholesterol trafficking is dependent upon NPC2 interaction with lysobisphosphatidic acid. eLife *8*, e50832. https://doi.org/10.7554/eLife.50832.

62. Kunkel, T.J., Townsend, A., Sullivan, K.A., Merlet, J., Schuchman, E.H., Jacobson, D.A., and Lieberman, A.P. (2023). The cholesterol transporter NPC1 is essential for epigenetic regulation and maturation of oligodendrocyte lineage cells. Nat. Commun. *14*, 3964. https://doi.org/10.1038/s41467-023-39733-6.

63. Ewels, P., Magnusson, M., Lundin, S., and Käller, M. (2016). MultiQC: summarize analysis results for multiple tools and samples in a single report. Bioinformatics *32*, 3047–3048. https://doi.org/10.1093/bioinformatics/btw354.

64. Dobin, A., Davis, C.A., Schlesinger, F., Drenkow, J., Zaleski, C., Jha, S., Batut, P., Chaisson, M., and Gingeras, T.R. (2013). STAR: ultrafast universal RNA-seq aligner. Bioinformatics *29*, 15–21. https://doi.org/10.1093/bioinformatics/bts635.

65. Yates, A.D., Achuthan, P., Akanni, W., Allen, J., Allen, J., Alvarez-Jarreta, J., Amode, M.R., Armean, I.M., Azov, A.G., Bennett, R., et al. (2020). Ensembl 2020. Nucleic Acids Res. *48*, D682–D688. https://doi.org/10.1093/nar/gkz966.

66. Liao, Y., Smyth, G.K., and Shi, W. (2014). featureCounts: an efficient general purpose program for assigning sequence reads to genomic features. Bioinformatics *30*, 923–930. https://doi.org/10.1093/bioinformatics/btt656.

67. Love, M.I., Huber, W., and Anders, S. (2014). Moderated estimation of fold change and dispersion for RNA-seq data with DESeq2. Genome Biol. *15*, 550. https://doi.org/10.1186/s13059-014-0550-8.

68. Baldarelli, R.M., Smith, C.L., Ringwald, M., Richardson, J.E., and Bult, C.J.; Mouse Genome Informatics Group (2024). Mouse Genome Informatics: an integrated knowledgebase system for the laboratory mouse. Genetics *227*, iyae031. https://doi.org/10.1093/genetics/iyae031.

69. Kainer, D., Lane, M., Sullivan, K.A., Miller, J.I., Cashman, M., Morgan, M., Cliff, A., Romero, J., Walker, A., Dakota Blair, D., et al. (2025). RWRtoolkit: multi-omic network analysis using random walks on multiplex networks in any species. GigaScience *14*, giaf028. https://doi.org/10.1093/gigascience/giaf028.

70. Kim, C.Y., Baek, S., Cha, J., Yang, S., Kim, E., Marcotte, E.M., Hart, T., and Lee, I. (2022). HumanNet v3: an improved database of human gene networks for disease research. Nucleic Acids Res. *50*, D632–D639. https://doi.org/10.1093/nar/gkab1048.

71. Cliff, A., Romero, J., Kainer, D., Walker, A., Furches, A., and Jacobson, D. (2019). A High-Performance Computing Implementation of Iterative Random Forest for the Creation of Predictive Expression Networks. Genes *10*, 996. https://doi.org/10.3390/genes10120996.

72. Eraslan, G., Drokhlyansky, E., Anand, S., Fiskin, E., Subramanian, A., Slyper, M., Wang, J., Van Wittenberghe, N., Rouhana, J.M., Waldman, J., et al. (2022). Single-nucleus cross-tissue molecular reference maps toward understanding disease gene function. Science *376*, eabl4290. https://doi.org/10.1126/science.abl4290.

73. Benjamini, Y., and Hochberg, Y. (1995). Controlling the False Discovery Rate: A Practical and Powerful Approach to Multiple Testing. J. Roy. Stat. Soc. B *57*, 289–300. https://doi.org/10.1111/j.2517-6161.1995.tb02031.x.

74. Lun, A.T.L., Riesenfeld, S., Andrews, T., Dao, T.P., Gomes, T.; participants in the 1st Human Cell Atlas Jamboree, and Marioni, J.C. (2019). EmptyDrops: distinguishing cells from empty droplets in droplet-based single-cell RNA sequencing data. Genome Biol. *20*, 63. https://doi.org/10.1186/s13059-019-1662-y.

75. Wickham, H. (2016). ggplot2: Elegant Graphics for Data Analysis (Springer-Verlag). https://link.springer.com/book/10.1007/978-3-319-24277-4.

76. Linderman, G.C., Zhao, J., Roulis, M., Bielecki, P., Flavell, R.A., Nadler, B., and Kluger, Y. (2022). Zero-preserving imputation of single-cell RNA-seq data. Nat. Commun. *13*, 192. https://doi.org/10.1038/s41467-021-27729-z.

77. Hao, Y., Stuart, T., Kowalski, M.H., Choudhary, S., Hoffman, P., Hartman, A., Srivastava, A., Molla, G., Madad, S., Fernandez-Granda, C., and Satija, R. (2024). Dictionary learning for integrative, multimodal and scalable single-cell analysis. Nat. Biotechnol. *42*, 293–304. https://doi.org/10.1038/s41587-023-01767-y.

78. Hao, Y., Hao, S., Andersen-Nissen, E., Mauck, W.M., Zheng, S., Butler, A., Lee, M.J., Wilk, A.J., Darby, C., Zager, M., et al. (2021). Integrated analysis of multimodal single-cell data. Cell *184*, 3573–3587.e29. https://doi.org/10.1016/j.cell.2021.04.048.

79. Stuart, T., Butler, A., Hoffman, P., Hafemeister, C., Papalexi, E., Mauck, W.M., Hao, Y., Stoeckius, M., Smibert, P., and Satija, R. (2019). Comprehensive Integration of Single-Cell Data. Cell *177*, 1888–1902.e21. https://doi.org/10.1016/j.cell.2019.05.031.

80. Butler, A., Hoffman, P., Smibert, P., Papalexi, E., and Satija, R. (2018). Integrating single-cell transcriptomic data across different conditions, technologies, and species. Nat. Biotechnol. *36*, 411–420. https://doi.org/10.1038/nbt.4096.

81. Satija, R., Farrell, J.A., Gennert, D., Schier, A.F., and Regev, A. (2015). Spatial reconstruction of single-cell gene expression data. Nat. Biotechnol. *33*, 495–502. https://doi.org/10.1038/nbt.3192.

82. Bland, J.M., and Altman, D.G. (1995). Multiple significance tests: the Bonferroni method. Br. Med. J. *310*, 170. https://doi.org/10.1136/bmj.310.6973.170.

OMTN, Volume 37

Supplemental information

Codon-optimized *Npc1* mRNA corrects Niemann-Pick type C1 disease phenotypes *in vitro* and *in vivo*

Ellen R. Koufer, Alice Townsend, Youngseo Na, Adele B. Correia, Noah K. Latham, Koralege C. Pathmasiri, Olga Ilnytska, Judith Storch, Kyle A. Sullivan, Troy Halseth, Thaddeus J. Kunkel, Elizabeth S. Phillippi, Matthew L. Lieberman, Stephanie M. Cologna, Andrew P. Lieberman, Daniel A. Jacobson, Anna S. Schwendeman, and Mark L. Schultz

Supplemental Figures

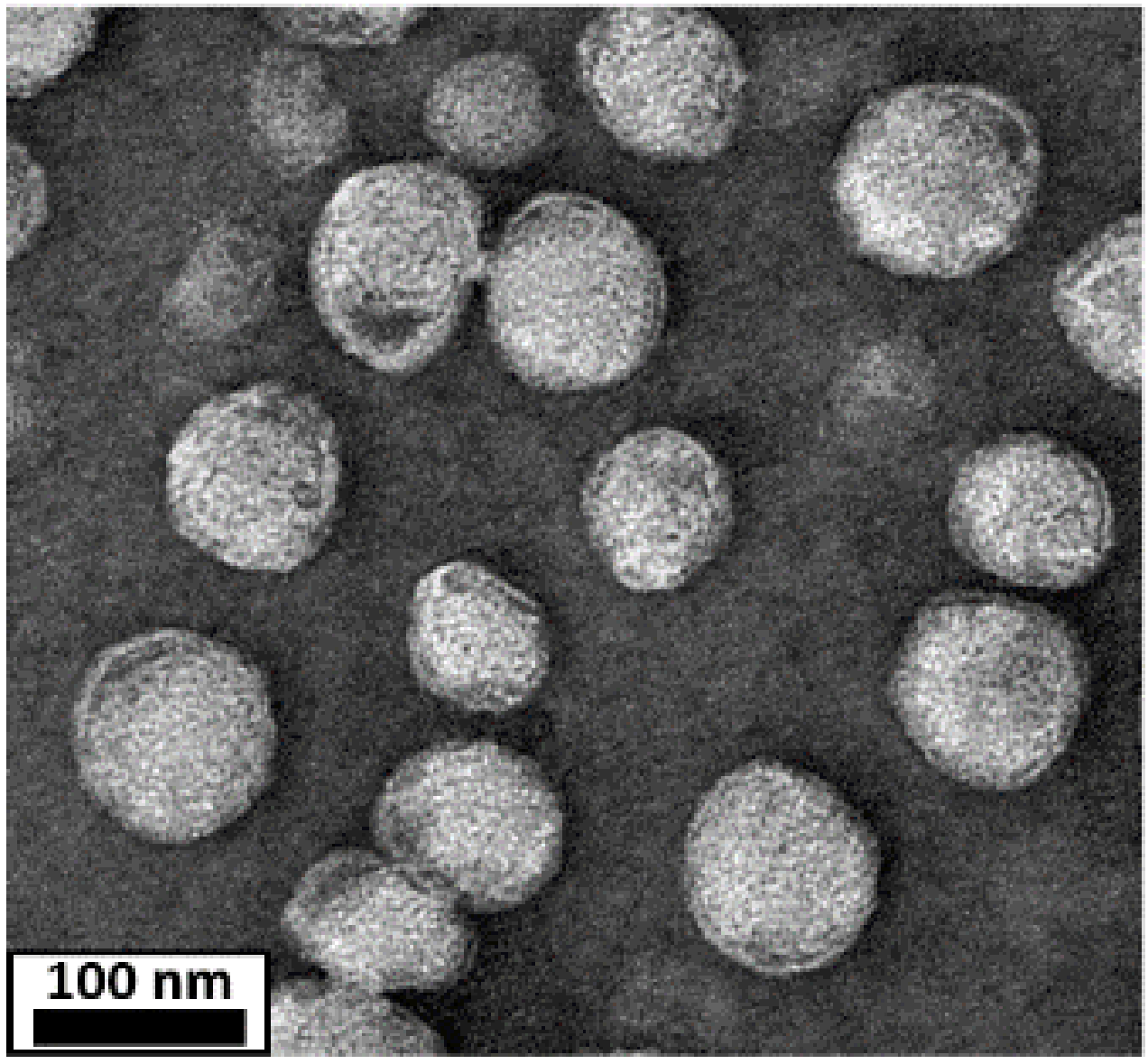

Figure S1. Transmission electron microscopy showing *GFP*:LNP particle diameter. Scale bar = 100 nm.

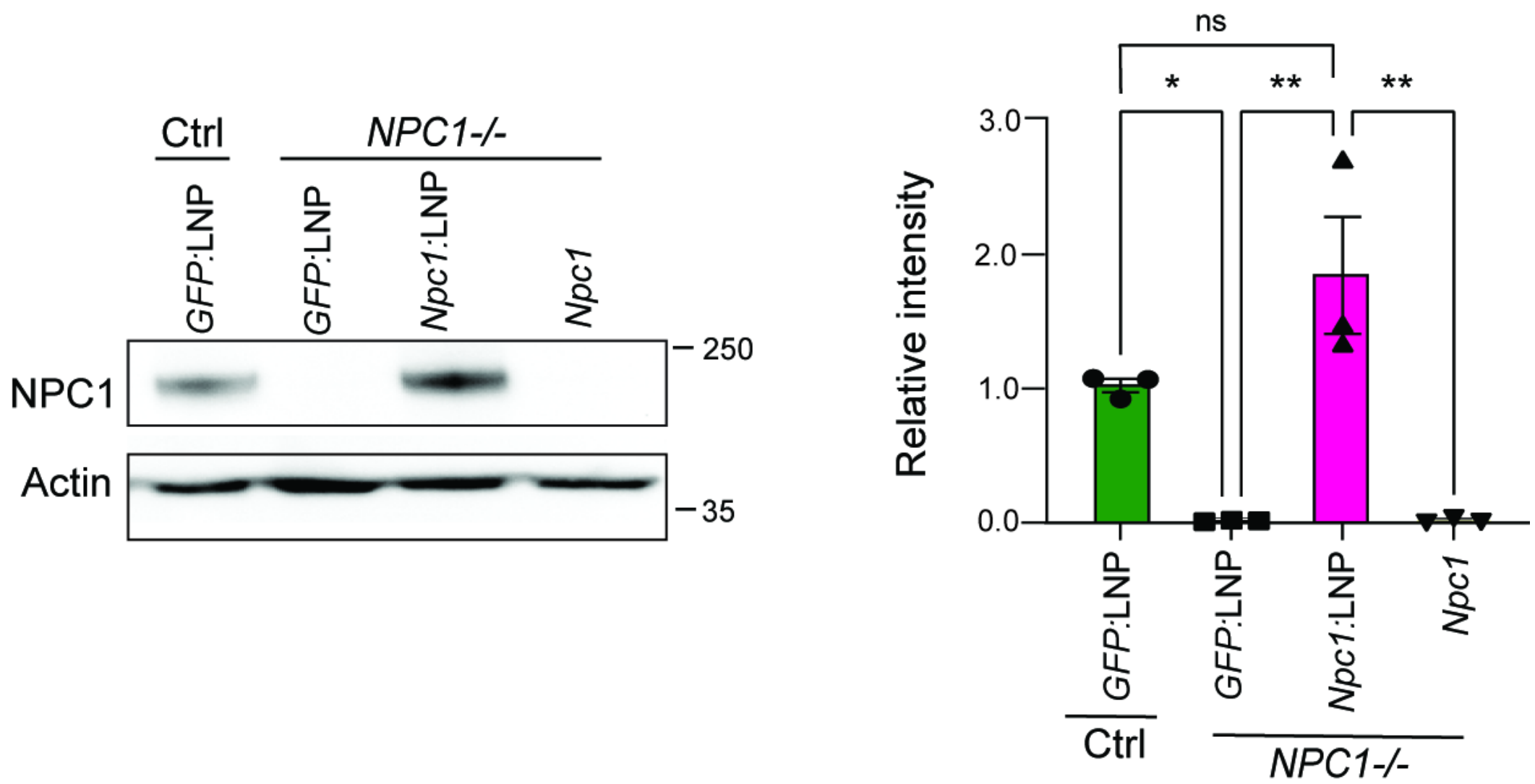

Figure S2. Total protein western blot for NPC1 after *Npc1*:LNP delivery. HeLa cells homozygous for null NPC1 (*NPC1*$^{-/-}$) or WT NPC1 (Ctrl) were treated for 24 hours with 1 µg lipid particle containing *GFP* or *Npc1* mRNA (*GFP*:LNP, *Npc1*:LNP), or 'naked' *Npc1* mRNA (not encapsulated within an LNP). After the 24-hour treatment, cells were given fresh media and analyzed 24 hours later by western blot for NPC1 protein levels. ns, not significant, *$p \leq .05$, **$p \leq .01$, ****$p \leq .0001$ by one-way ANOVA with Tukey's multiple comparisons test: F (3, 8) = 16.39. Data are shown as the mean ± SEM from n = 3/group.

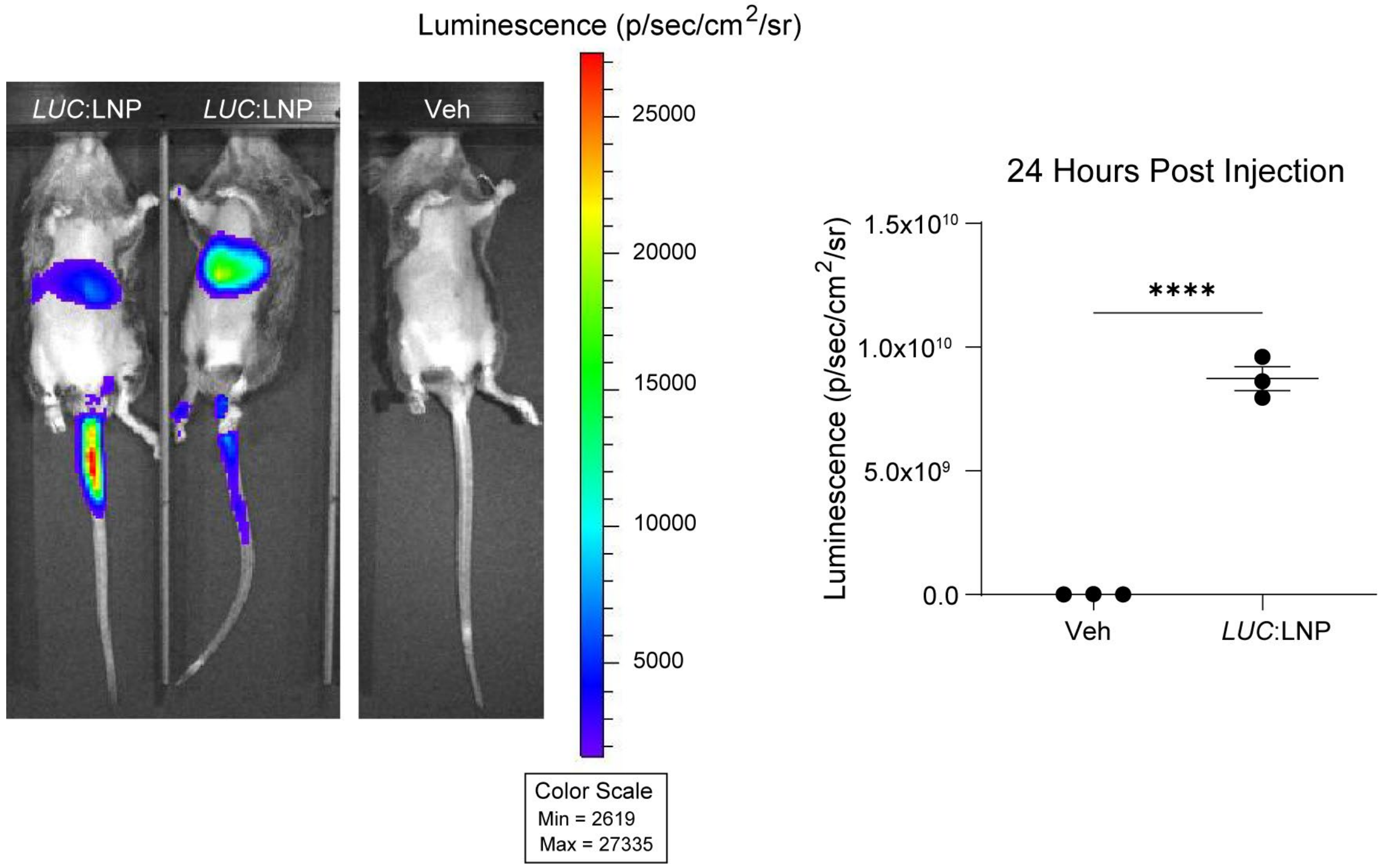

Figure S3. Image from the PerkinElmer IVIS Lumina optical imaging system showing localization of bioluminescent activity after LNP injection. Mice were injected once intravenously with 200 µL saline or 1 mg/kg of LNP containing luciferase mRNA (*LUC*:LNP). 24 hours after injection, mice were injected with 15 mg/kg d-luciferin and imaging was performed 20 minutes later. ****$p \leq 0.0001$ by unpaired t test: ($t=18.09$, $df=4$). Data are shown as the mean ± SEM from n = 3/group.

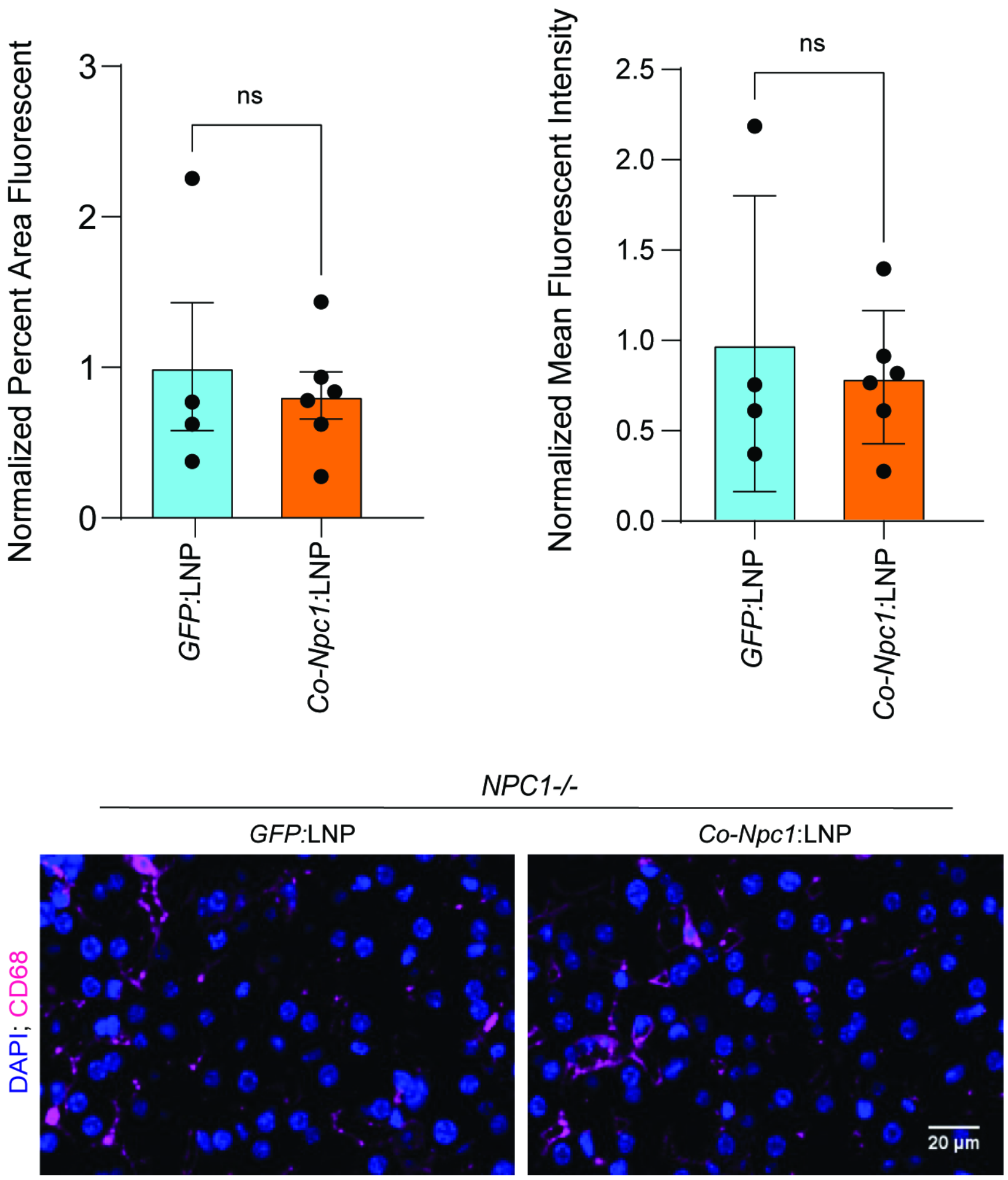

Figure S4. Macrophage populations in the mouse liver. *Npc1*$^{-/-}$ mice were injected intravenously with 1 mg/kg of *GFP*:LNP or *Co-Npc1:*LNP. 48 hours after injeciton, livers were perfused, collected, and stained with the macrophage marker CD68 (pink) and DAPI (blue). ns, not significant by unpaired t test: (t=0.4927, df=8), (t=0.4927, df=8), from left to right. Data are shown as the mean ± SEM from n = 4-6/group.

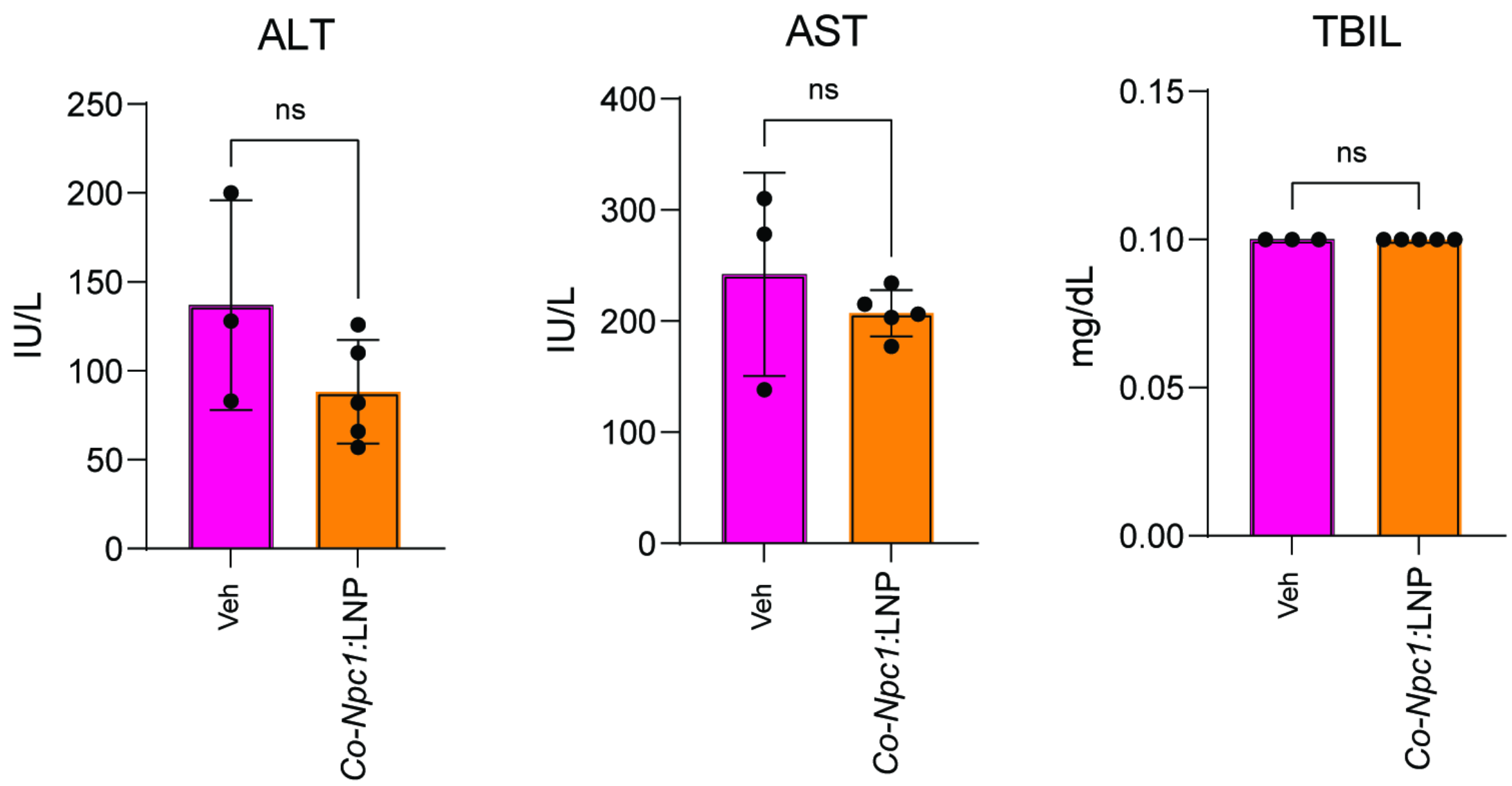

Figure S5. Liver enzyme tests 4 days after *Co-Npc1*:LNP treatment in *Npc1*$^{-/-}$ mice. 6-week-old *Npc1*$^{-/-}$ mice were injected once intravenously with 200 µL saline (Veh) or 1 mg/kg *Co-Npc1*:LNP. After 4 days, livers were harvested and analyzed for levels of the enzymes alanine aminotransferase (ALT), aspartate aminotransferase (AST), and total bilirubin (TBIL). ns, not significant by unpaired t test: (t=1.607, df=6), (t=0.8644, df=6), cannot conduct test due to identical values, from left to right. All values fell within the normal range for mouse liver enzyme function values. Data are shown as the mean ± SEM from n = 3-5/group.

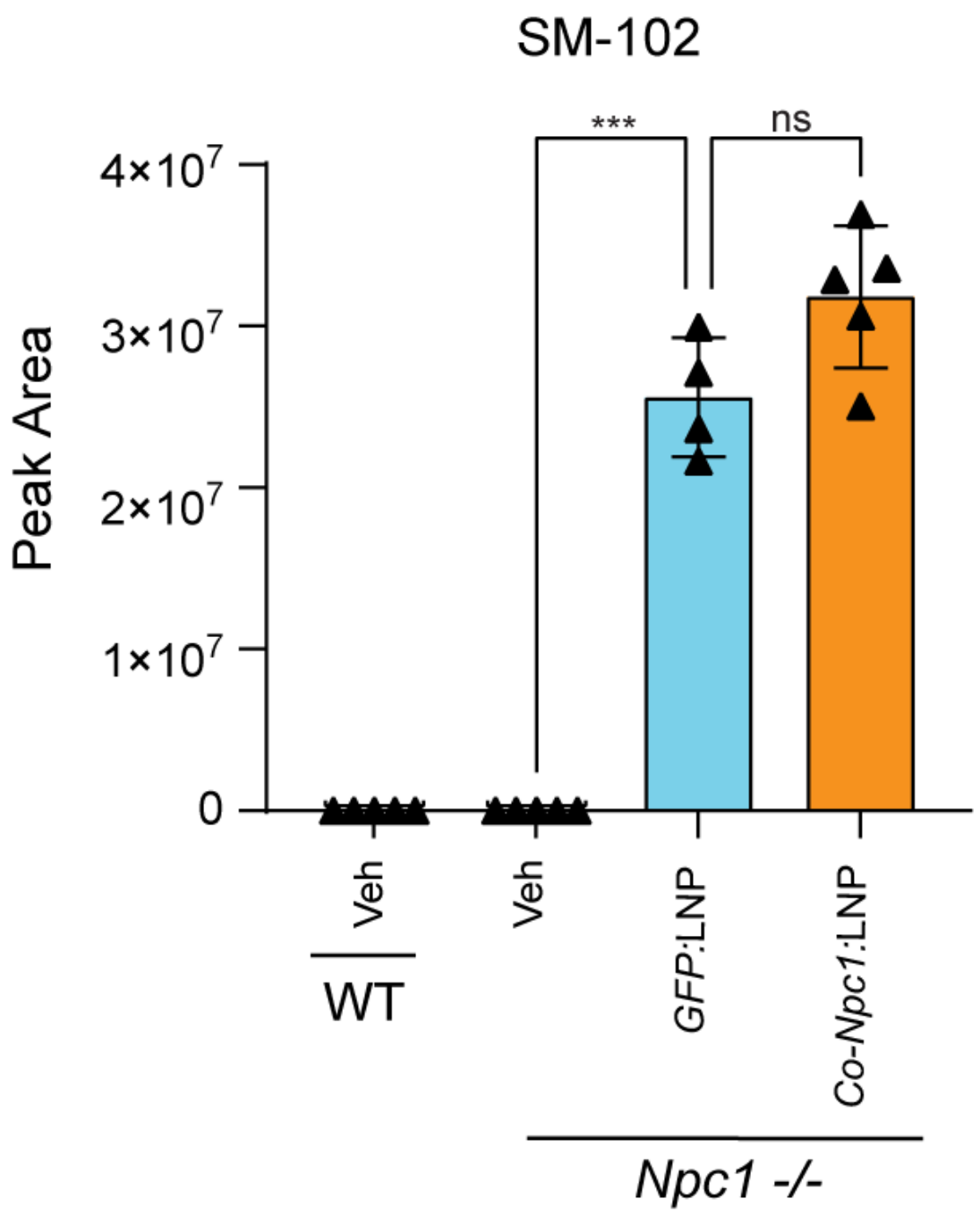

Figure S6. 3-week-old *Npc1*$^{+/+}$ (WT) and *Npc1*$^{-/-}$ mice treated with saline (Veh), 1 mg/kg *GFP*:LNP, or 1 mg/kg codon-optimized *Co-Npc1*:LNP. Livers were collected 48 hours after treatment. Levels of the synthetic lipid SM-102 were analyzed by liquid-chromatography-mass spectrometry. ns, not significant, ***$p \leq .001$ by one-way ANOVA with Tukey's multiple comparisons test; $F(3, 15) = 172$. Data are shown as the mean ± SEM from n = 3-5/group. Data points plotted as 0 represent samples in which SM-102 was below the limit of detection.

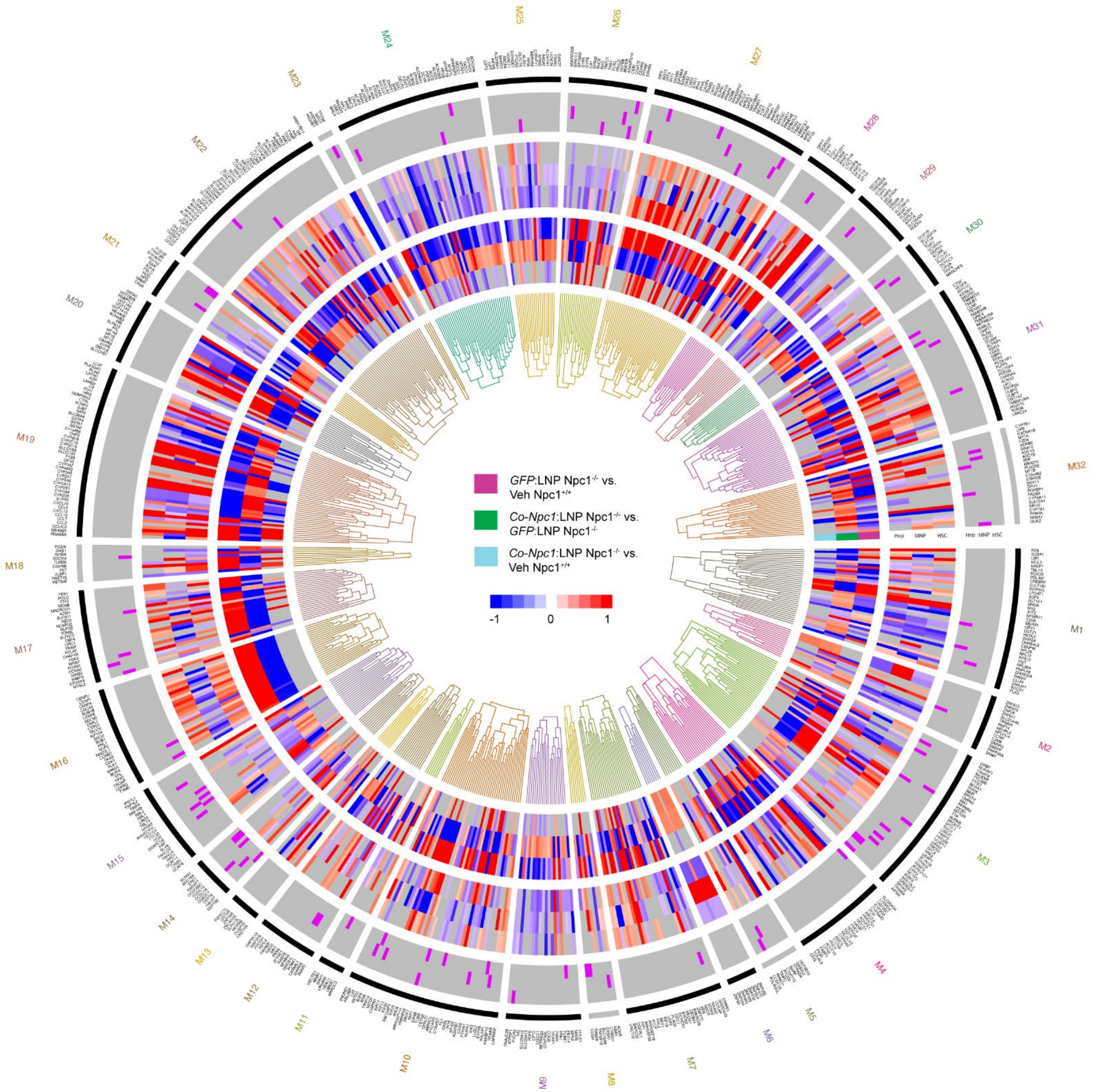

Figure S7. Integration of bulk RNA-seq data and snRNA-seq data reveals transcriptional correction across liver cell type populations following *Co-Npc1*:LNP treatment. Polar dendrogram visualization of MENTOR-derived gene modules integrating bulk RNA-seq and single-nucleus RNA-seq data. Genes are organized based on hierarchical clustering of RWR-derived dissimilarities across multiplex network, with modules (M1-M32) annotated along the outer ring. Heatmap tracks follow the module labels. The innermost heatmap track displays gene-level $\log_2$(FC) values for the bulk RNA-seq data. For the innermost track and the following tracks, the innermost column of displays *GFP:*LNP *Npc1*$^{-/-}$ vs. Vehicle *Npc1*$^{+/+}$, representing disease-associated transcriptional changes. The middle column shows *Co-Npc1*:LNP *Npc1*$^{-/-}$ vs. *GFP*:LNP *Npc1*$^{-/-}$, capturing treatment-induced transcriptional shifts. The last column represents *Co-Npc1*:LNP *Npc1*$^{-/-}$ vs. Vehicle *Npc1*$^{+/+}$, where non-differentially expressed genes (indicated in grey) show correction toward wild-type expression levels. Color scale ranges from blue (downregulation) to red (upregulation). The next heatmap (middle) track incorporates snRNA-seq results for hepatocytes (Hep), mononuclear phagocytes (MNP), and hepatic stellate cells (HSCs), displaying

the $\log_2$(FC) values for each cell type. The next heatmap (outer) track indicates genes that are uniquely differentially expressed within a specific cell type (Hep, HSC, or MNP) and that also exhibit correction in the bulk RNA-seq data. The outermost track denotes module statistical significance, with black segments denoting modules that are significant relative to permutation-derived null distributions.

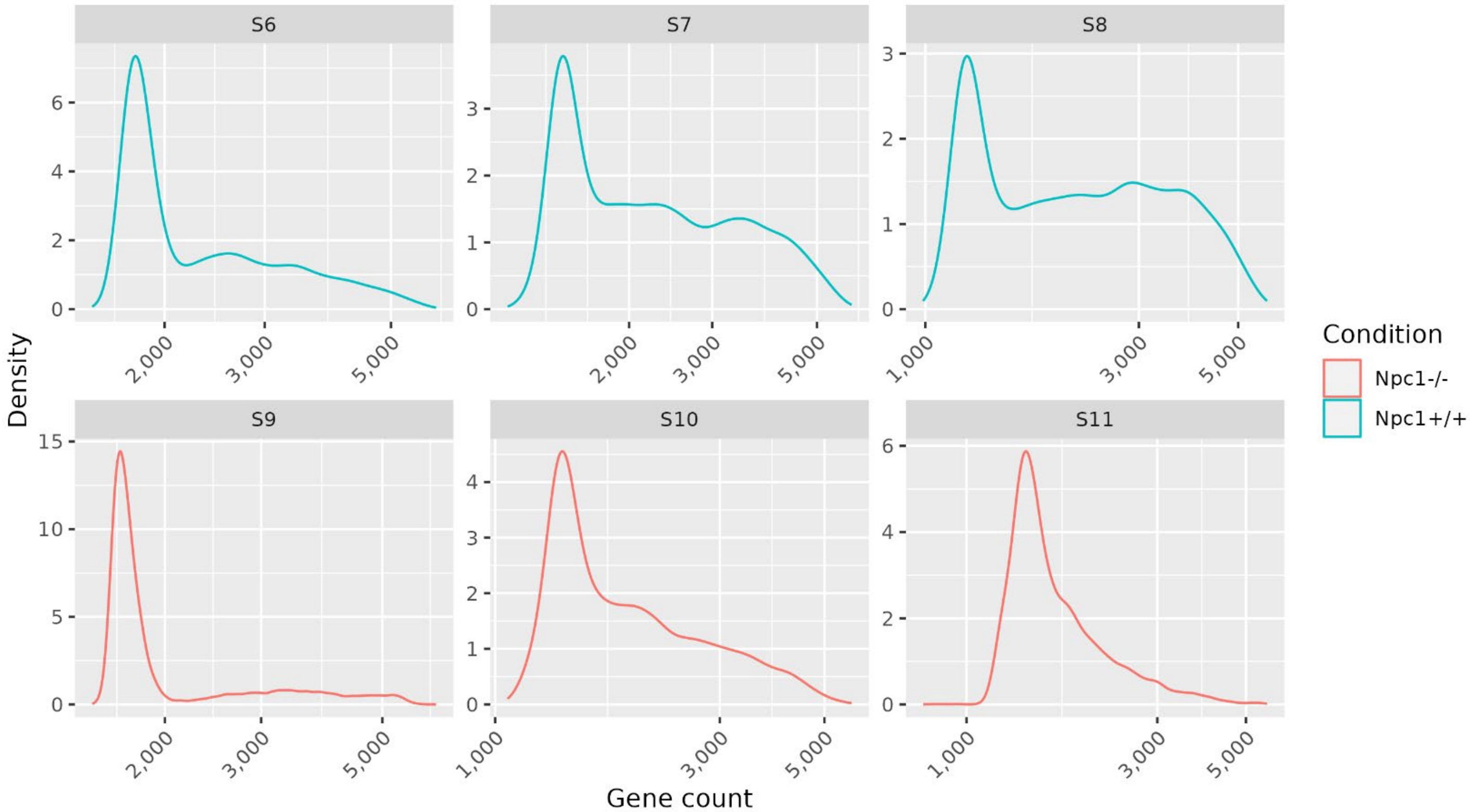

Figure S8. Single-nucleus RNA-sequencing data filtering. snRNA-seq was performed on liver samples from 7-week-old *Npc1*$^{+/+}$ and *Npc1*$^{-/-}$ mice. Distributions display unique gene count after quality control filtering per sample. n = 3/group.

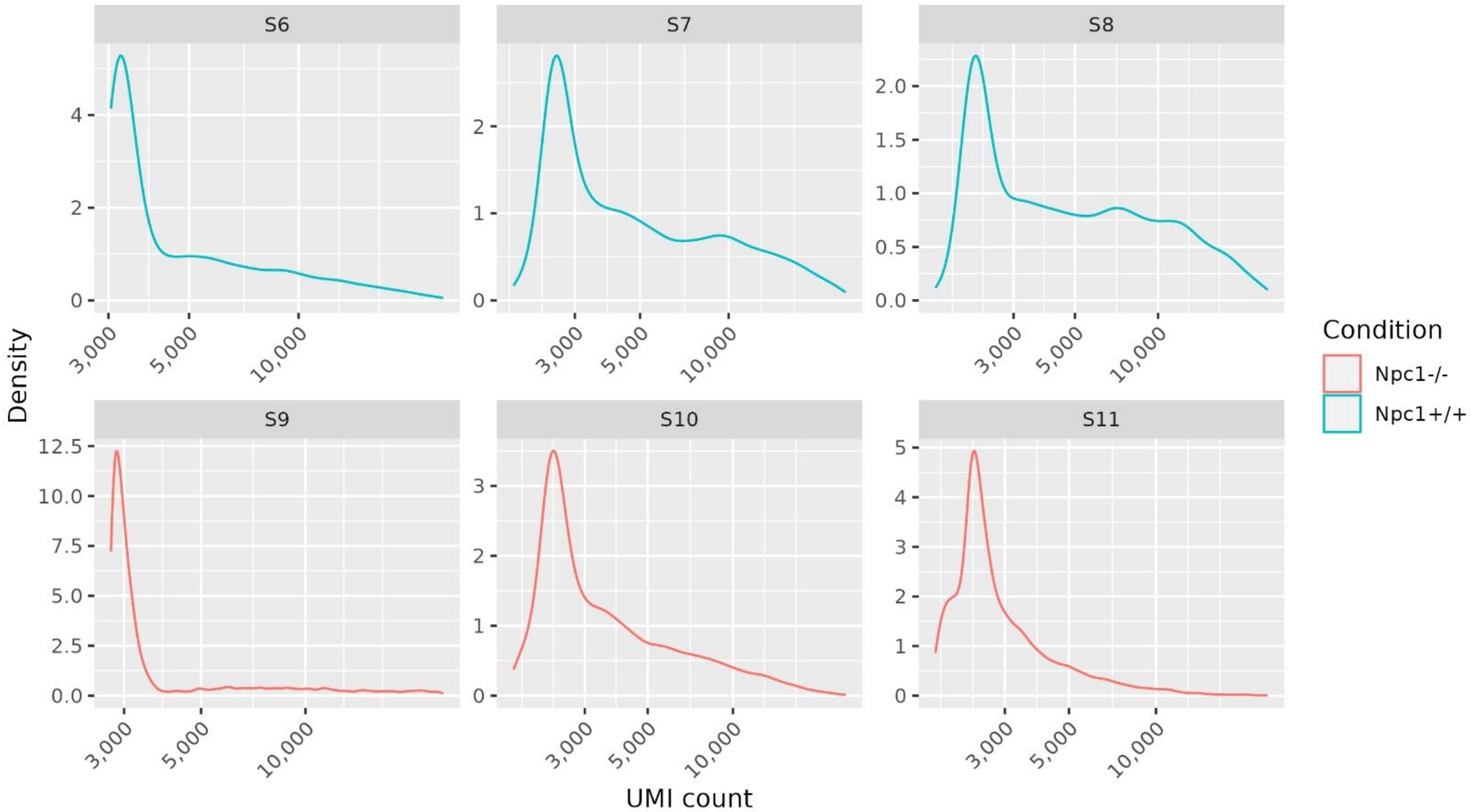

Figure S9. Single-nucleus RNA-sequencing data filtering. snRNA-seq was performed on liver samples from 7-week-old *Npc1*$^{+/+}$ and *Npc1*$^{-/-}$ mice. Distributions display total UMI after quality control filtering per sample. n=3/group.

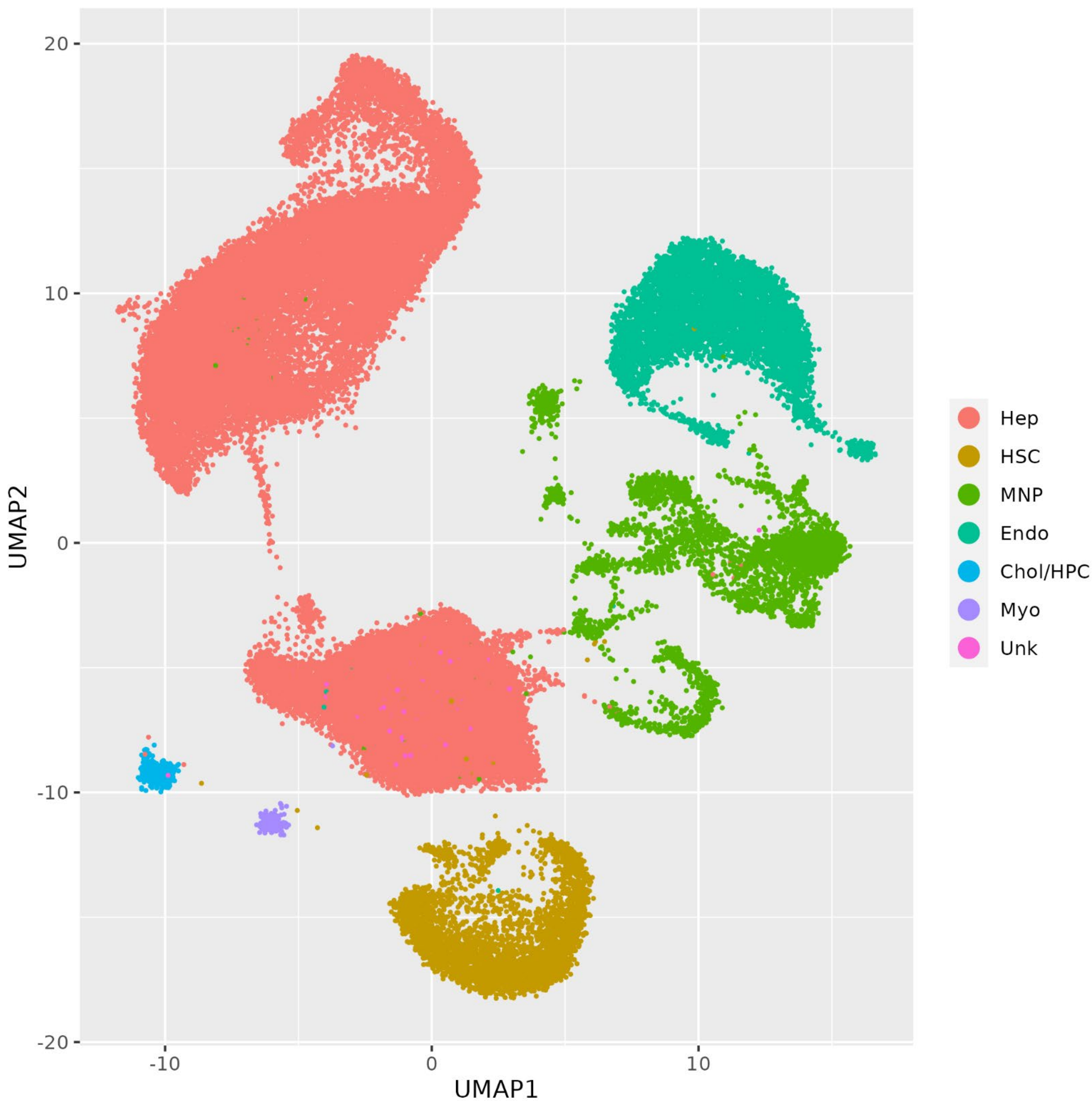

Figure S10. UMAP embeddings displaying results of Seurat dimensionality reduction, clustering, and differential expression analysis for manual annotation of major cell type populations. Cell types identified include hepatocytes (Hep), hepatic stellate cells (HSCs), mononuclear phagocytes (MNP), endothelial cells (Endo), cholinergic/hepatic progenitor cells (Chol/HPC), myofibroblasts (Myo), and a small population of unknown cells (Unk). Cell type clusters are preserved across genotypes.

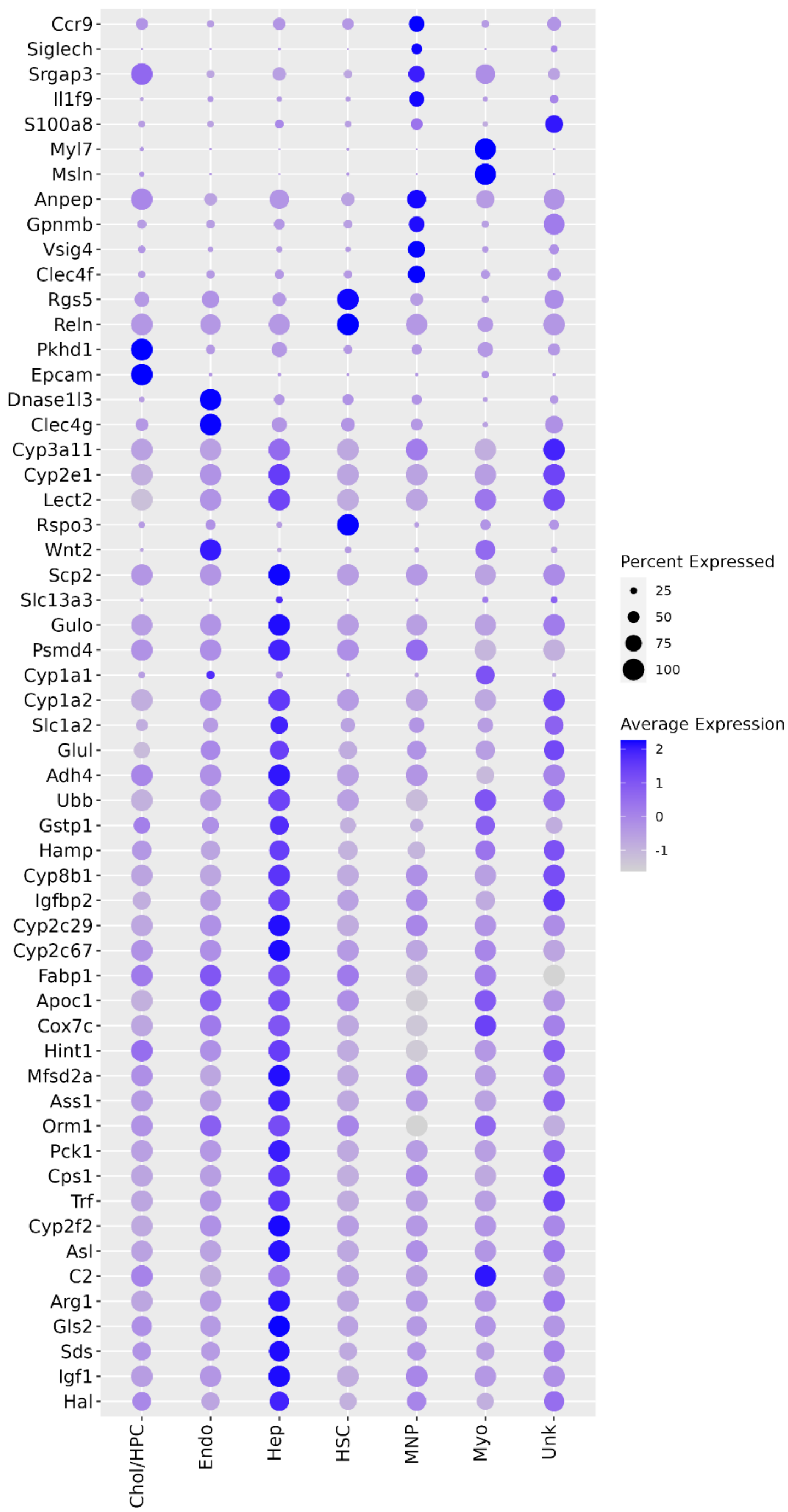

Figure S11. Dot plot visualization of cell type-specific marker gene expression across major cell type populations identified from snRNA-seq data. Rows correspond to genes and columns correspond to corresponding cell types, including hepatocytes (Hep), hepatic stellate cells (HSCs), mononuclear phagocytes (MNP), endothelial cells (Endo), cholinergic/hepatic progenitor cells (Chol/HPC), myofibroblasts (Myo), and a small population of unknown cells (Unk). Size of points represent percentages of cells within each cluster expressing the marker gene and color intensity represents scaled gene expression, ranging from low (light purple) to high (blue).

```
   1 ATGGGAGCTC ATCACCCAGC TCTGGGACTT CTTTTGCTTC TGCTCTGTCC AGCACAAGTT
  61 TTTTCACAAT CATGTGTATG GTACGGGGAG TGTGGAATAG CAACCGGCGA TAAACGCTAT
 121 AATTGTAAAT ATTCAGGACC CCCAAAGCCC CTGCCAAAGG ACGGATATGA TTTGGTGCAG
 181 GAGCTCTGTC CTGGCTTGTT TTTTGATAAC GTGTCACTGT GTTGTGACAT ACAACAACTC
 241 CAGACTCTGA AGAGCAATCT TCAATTGCCC CTCCAGTTTC TCTCTAGGTG TCCATCTTGC
 301 TTTTACAATC TTATGACCCT TTTTTGCGAG TTGACTTGTT CACCACATCA GAGTCAATTC
 361 TTGAACGTCA CCGCCACCGA GGATTACTTT GACCCTAAGA CACAAGAAAA TAAGACTAAT
 421 GTGAAGGAGT TGGAATACTT CGTTGGGCAG TCATTCGCAA ACGCAATGTA CAATGCTTGC
 481 CGAGACGTGG AAGCACCCTC CAGCAACGAA AAAGCCCTTG GCCTTCTGTG TGGGCGGGAT
 541 GCTCGCGCAT GCAATGCCAC AAACTGGATC GAATACATGT TCAACAAGGA CAATGGTCAG
 601 GCCCCTTTTA CTATCATACC CGTATTCTCT GATTTGTCCA TATTGGGCAT GGAACCAATG
 661 AGAAACGCTA CTAAGGGGTG CAATGAATCA GTGGATGAAG TGACTGGACC TTGCAGCTGT
 721 CAAGATTGTT CCATTGTCTG TGGTCCAAAA CCACAGCCAC CTCCTCCACC AATGCCATGG
 781 CGCATATGGG GACTCGATGC AATGTACGTC ATCATGTGGG TCACTTATGT CGCCTTCCTT
 841 TTTGTTTTTT TTGGCGCACT TTTGGCAGTA TGGTGCCATC GGCGACGATA TTTCGTATCT
 901 GAGTATACTC CCATAGATTC TAATATCGCT TTCAGTGTTA ATTCAAGCGA CAAAGGAGAA
 961 GCCTCCTGCT GCGATCCTCT CGGAGCTGCC TTTGACGACT GTCTCAGACG CATGTTCACC
1021 AAGTGGGGAG CCTTTTGTGT CCGCAATCCA ACTTGTATCA TCTTTTTCTC CTTGGCATTT
1081 ATTACCGTGT GCAGTTCCGG ACTGGTTTTT GTCCAAGTTA CTACAAATCC AGTGGAACTG
1141 TGGAGTGCAC CCCACAGCCA AGCCAGACTG GAAAAGGAAT ACTTTGACAA GCACTTTGGA
1201 CCTTTCTTCC GCACAGAACA GCTGATAATC CAGGCCCCAA ATACCAGTGT GCATATATAT
1261 GAGCCTTATC CCGCCGGCGC CGACGTGCCT TTCGGTCCCC CTCTTAACAA AGAGATCCTC
1321 CATCAAGTCC TGGATCTCCA GATAGCCATC GAAAGTATCA CTGCAAGCTA CAACAATGAG
1381 ACTGTTACCT TGCAAGATAT ATGTGTAGCC CCACTGTCTC CCTATAATAA AAATTGTACA
1441 ATAATGTCCG TGCTCAATTA CTTTCAAAAT AGTCACGCTG TTTTGGACAG TCAAGTCGGC
1501 GATGATTTCT ACATCTATGC CGATTACCAC ACCCATTTTC TGTATTGCGT CCGAGCCCCT
1561 GCCAGCCTGA ACGACACAAG TCTTCTTCAC GGCCCATGTT TGGGTACCTT CGGAGGTCCC
1621 GTCTTTCCCT GGCTCGTGCT TGGAGGCTAC GACGATCAAA ATTACAATAA TGCCACAGCT
1681 CTGGTCATAA CTTTTCCTGT GAATAACTAC TACAACGACA CTGAAAGGTT GCAACGCGCC
1741 TGGGCCTGGG AAAAGGAGTT CATTAGTTTC GTTAAGAATT ACAAGAATCC AAATTTGACC
1801 ATCTCATTCA CTGCAGAACG GTCAATCGAA GATGAACTCA ACCGCGAAAG TAACTCTGAC
1861 GTATTCACCG TTATAATCTC CTATGTTGTG ATGTTTCTGT ACATCTCACT TGCATTGGGA
1921 CATATTCAAT CATGTTCACG GCTCCTCGTC GACTCTAAGA TCAGCTTGGG GATAGCCGGG
1981 ATTTTGATCG TACTGTCAAG TGTGGCCTGT AGCCTTGGGA TCTTTTCTTA TATGGGTATG
2041 CCACTGACAC TGATAGTGAT AGAGGTGATA CCTTTCTTGG TATTGGCCGT GGGGGTAGAT
2101 AACATTTTCA TTCTGGTCCA AACCTACCAA CGCGACGAAA GGCTGCAAGA GGAGACCCTG
2161 GACCAACAAC TCGGGAGAAT CCTTGGAGAG GTCGCCCCCA CAATGTTCCT TTCATCATTC
2221 TCCGAAACAT CTGCATTTTT CTTCGGTGCC CTTAGCTCCA TGCCCGCTGT TCATACTTTC
2281 AGCCTGTTTG CAGGAATGGC AGTCCTCATA GATTTTCTGT TGCAAATTAC CTGCTTCGTA
2341 TCATTGCTTG GCTTGGACAT AAAACGACAG GAAAAAAATC ATCTCGACAT ATTGTGCTGT
2401 GTACGCGGCG CCGACGACGG CCAAGGCTCA CACGCTTCTG AATCCTACCT GTTTAGGTTC
2461 TTCAAGAATT ACTTCGCACC CCTGCTCTTG AAAGACTGGC TTAGACCTAT TGTAGTAGCC
2521 GTGTTCGTCG GTGTACTGTC TTTCAGTGTA GCCGTCGTGA ATAAGGTCGA CATAGGGTTG
2581 GATCAATCCT TGTCCATGCC TAATGATAGC TATGTCATAG ACTACTTCAA GAGTCTGGCC
2641 CAGTACTTGC ATTCCGGACC TCCTGTTTAT TTTGTACTGG AGGAAGGATA TAATTTATTCA
2701 AGTCGCAAGG GACAGAATAT GGTGTGTGGT GGAATGGGGT GCGACAACGA CTCACTGGTA
2761 CAACAGATCT TTAACGCCGC CGAATTGGAT ACATACACTA GGGTCGGGTT CGCCCCCTCT
2821 TCATGGATTG ATGACTACTT CGATTGGGTG AGCCCTCAAT CAAGCTGTTG TAGGCTCTAT
2881 AATGTAACAC ACCAGTTCTG TAACGCCTCC GTCATGGACC CAACTTGTGT TAGATGTCGC
2941 CCACTCACAC CCGAAGGAAA ACAAAGACCA CAGGGGAAAG AATTTATGAA GTTCCTCCCT
3001 ATGTTTTTGA GTGATAACCC AAATCCTAAG TGCGGAAAAG GGGGCCACGC TGCCTATGGC
3061 TCTGCAGTCA ACATCGTTGG CGATGATACA TACATTGGTG CCACTTACTT TATGACATAC
3121 CATACAATAC TTAAAACATC AGCCGACTAT ACTGACGCTA TGAAAAAGGC TAGGCTCATC
3181 GCATCAAACA TTACTGAAAC TATGCGCAGT AAGGGGAGTG ACTATCGAGT ATTCCCATAT
3241 AGTGTATTCT ACGTATTCTA TGAGCAATAT CTTACTATCA TCGATGATAC AATCTTTAAT
3301 CTTTCCGTTA GTCTGGGTTC TATTTTTTTG GTGACTCTCG TAGTTCTTGG GTGTGAATTG
3361 TGGTCCGCCG TAATAATGTG TATTACAATA GCTATGATTC TTGTTAATAT GTTTGGCGTG
3421 ATGTGGTTGT GGGGGATCTC CCTCAATGCT GTGTCATTGG TGAACTTGGT GATGAGCTGT
3481 GGAATCAGTG TAGAGTTCTG CAGTCACATC ACTAGAGCTT TTACCATGAG TACCAAGGGG
3541 TCTAGGGTTA GCCGAGCTGA GGAGGCTCTG GCCCACATGG GGAGTTCTGT ATTTTCTGGA
3601 ATTACACTGA CAAAATTTGG AGGCATCGTA GTGCTGGCAT TCGCCAAGTC TCAAATCTTT
3661 GAGATCTTCT ACTTTCGGAT GTATCTTGCT ATGGTATTGC TTGGCGCAAC CCACGGCCTT
3721 ATCTTCCTGC CCGTACTCTT GAGCTACATT GGTCCTAGTG TTAACAAGGC CAAGCGACAC
3781 ACCACATACG AACGGTACAG GGGTACCGAG AGAGAGCGGC TGCTTAATTT CTGA
```

Figure S12. mRNA sequence for codon-optimized *Npc1.* Base differences between codon-optimized and endogenous *Npc1* are delineated by bold and elevated text

Supplemental Tables

Table S1. Particle characterization of *GFP*:LNP, *Npc1*:LNP, and *Co-Npc1*:LNP formulations. Particle Size, polydispersity Index (PDI), and encapsulation efficiency (EE) are shown.

**Formulation**	**Size (nm)**	**PDI**	**EE**
GFP:LNP	84	0.097	99%
Npc1:LNP	100.9	0.127	98%
Co-Npc1:LNP	94.35	0.126	96%

Table S2. MENTOR-IA themes describing each module within the MENTOR dendrogram. Found in supplemental spreadsheet file.

Table S3. Marker genes used for cell type clustering of snRNA-seq data.

Marker gene symbol	Liver cell type
Hal	Hepatocytes (periportal)
Igf1	Hepatocytes (periportal)
Sds	Hepatocytes (periportal)
Gls2	Hepatocytes (periportal)
Arg1	Hepatocytes (periportal)
C2	Hepatocytes (periportal)
Asl	Hepatocytes (periportal)
Cyp2f2	Hepatocytes (periportal)
Trf	Hepatocytes (periportal)
Cps1	Hepatocytes (periportal)
Pck1	Hepatocytes (periportal)
Orm1	Hepatocytes (periportal)
Ass1	Hepatocytes (periportal)
Mfsd2a	Hepatocytes (periportal)
Hint1	Hepatocytes (midzonal)
Cox7c	Hepatocytes (midzonal)
Apoc1	Hepatocytes (midzonal)
Fabp1	Hepatocytes (midzonal)
Cyp2c67	Hepatocytes (midzonal)
Cyp2c29	Hepatocytes (midzonal)
Igfbp2	Hepatocytes (midzonal)
Cyp8b1	Hepatocytes (midzonal)
Hamp	Hepatocytes (midzonal)
Gstp1	Hepatocytes (midzonal)
Ubb	Hepatocytes (midzonal)
Adh4	Hepatocytes (pericentral)
Glul	Hepatocytes (pericentral)
Slc1a2	Hepatocytes (pericentral)
Cyp1a2	Hepatocytes (pericentral)
Cyp1a1	Hepatocytes (pericentral)
Psmd4	Hepatocytes (pericentral)
Gulo	Hepatocytes (pericentral)
Slc13a3	Hepatocytes (pericentral)
Scp2	Hepatocytes (pericentral)
Wnt2	Hepatocytes (pericentral)
Rspo3	Hepatocytes (pericentral)
Lect2	Hepatocytes (pericentral)
Cyp2e1	Hepatocytes (pericentral)
Cyp2c29	Hepatocytes (pericentral)
Cyp3a11	Hepatocytes (pericentral)
Clec4g	Endothelial cells
Dnase1l3	Endothelial cells

Epcam	Cholangiocyte/hepatic progenitor cells
Pkhd1	Cholangiocyte/hepatic progenitor cells
Reln	Hepatic stellate cells
Rgs5	Hepatic stellate cells
Clec4f	Kupffer cells
Vsig4	Kupffer cells
Gpnmb	Macrophages
Anpep	Macrophages
Msln	Myofibroblasts
Myl7	Myofibroblasts
S100a8	Neutrophils
Il1f9	Neutrophils
Srgap3	Plasmacytoid dendritic cells
Siglech	Plasmacytoid dendritic cells
Ccr9	Plasmacytoid dendritic cells

Table S4. Differential cell-type abundance in livers from *Npc1*$^{+/+}$ and *Npc1*$^{-/-}$ mice. Average proportion of each cell type across all samples (BaselineProp.Freq), mean proportion of cell type in *Npc1*$^{-/-}$ samples (PropMean.KO), mean proportion of cell type in *Npc1*$^{+/+}$ samples (PropMean.WT), *Npc1*$^{-/-}$ proportion divided by *Npc1*$^{+/+}$ proportion (PropRatio), test statistic (Tstatistic), unadjusted p-value (P.Value), and false-discovery rate-adjusted p-value.

Cell Type	BaselineProp.Freq	PropMean.KO	PropMean.WT	PropRatio	Tstatistic	P.Value	FDR
MNP	0.0681	0.098	0.0395	2.4795	3.0395	0.0056	0.0339
Myo	0.0029	0.0018	0.0039	0.4799	-2.1346	0.0432	0.1296
Endo	0.0901	0.0677	0.1112	0.6084	-1.8502	0.0766	0.1533
HSC	0.0625	0.0494	0.0747	0.662	-1.5076	0.1447	0.217
Chol/HPC	0.0058	0.0052	0.0063	0.8217	-0.8721	0.3918	0.4701
Hep	0.7707	0.7779	0.7644	1.0176	0.3211	0.7509	0.7509

Table S5. Materials and reagents**.** Reagents used for described experiments, type of reagent, dilution used when applicable, and vendor with product number when applicable.

**Reagent/Kit**	**Type**	**Dilution**	**Vendor**
NPC1	Primary antibody	1:500	Abcam (ab134113/clone EPR5209)
GFAP	Primary antibody	1:1000	Abcam (AB290)
Actin	Primary antibody	1:2000	Millipore (Sigma A5441/clone AC-15)
LC3B	Primary antibody	1:500	Cell Signaling (3868S/(D11))
Vinculin	Primary antibody	1:2000	Sigma (V9131/clone hVIN-1)
CD68	Primary antibody	1:250	Santa Cruz Biotechnology (sc-20060)
Goat anti-mouse IgG (H+L)-HRP conjugate	Secondary antibody	1:2000	Bio-Rad (170-6516)
Goat anti-rabbit IgG (H+L)-HRP conjugate	Secondary antibody	1:2000	Bio-Rad (170-6515)
Wheat germ agglutinin 488	Stain	1:1000	Invitrogen (W11261)
Filipin	Stain	1μg	Sigma (SAE0087-1ML)
D-Luciferin, Potassium Salt	synthetic luciferin	NA	Invitrogen (L2916)
SM-102 (MW 710.2) (33474)	ionizable cationic lipid	NA	Cayman Chemical
Cholesterol (MW 386.65) (C8667)	Sterol	NA	Sigma Aldrich
1,2-distearoyl-sn-glycero-3-phosphocholine (DSPC, 18:0 PC; MW 790.2) (850365P)	Phospholipid	NA	Avanti Polar Lipids
1,2-dimyristoyl-rac-glycero-3-methoxypolyethylene glycol-2000 (DMG-PEG2000; MW 2526) (880151P)	pegylated lipid	NA	Avanti Polar Lipids
Quant-iT™ RiboGreen™ RNA Assay Kit (R11490)	Kit	NA	ThermoFisher Scientific
CleanCap® *EGFP* mRNA (L-7601)	*eGFP* mRNA	NA	TriLink Biotechnologies
CleanCap® *Npc1* mRNA (L-7601)	*Npc1* mRNA	NA	TriLink Biotechnologies
Npc1 mRNA qPCR probe	qPCR probe	NA	Integrated DNA technologies
Codon-optimized *Npc1* mRNA qPCR probe	taqMan Assay qPCR probe<br><br>5' to 3' for all primers and probes:<br><br>Forward Primer GCATTCGCCAAGTCTCAAATC	NA	Integrated DNA technologies

	Reverse Primer: AGAGTACGGGCAGGAAGATA<br><br>Probe: GCTATGGTATTGCTTGGCGCAA		

Table S6. Multiple Reaction Monitoring transitions and optimized collision energies for LC-MS analysis of cholesterol and cholesteryl esters

Compound name	Precursor m/z	Product m/z	Transition type	RT (min)	CE (V)	iFunnel mode
CE 16:0	642.6	369.3	Quantifier	10.2	10	Fragile
CE 16:0	642.6	161.1	Qualifier	10.2	43	Fragile
CE 18:0	670.6	369.3	Quantifier	12	10	Fragile
CE 18:0	670.6	161.1	Qualifier	12	43	Fragile
CE 18:2	666.6	369.3	Qualifier	9.2	10	Fragile
CE 18:2	666.6	161.1	Quantifier	9.2	43	Fragile
CE 20:4	690.6	369.3	Qualifier	8.6	10	Fragile
CE 20:4	690.6	161.1	Quantifier	8.6	43	Fragile
CE 22:6	714.6	369.3	Quantifier	8	10	Fragile
CE 22:6	714.6	161.1	Qualifier	8	43	Fragile
CE 16:0-d7	649.6	376.4	Quantifier	10.2	10	Fragile
CE 16:0-d7	649.6	147.2	Qualifier	10.2	45	Fragile
Cholesterol	369.3	161.1	Qualifier	4.1	18	Standard
Cholesterol	369.3	81	Quantifier	4.1	45	Standard
Cholesterol-d7	376.3	161.1	Qualifier	4.1	22	Standard
Cholesterol-d7	376.3	81.1	Quantifier	4.1	45	Standard

Supplemental Methods

Transmission Electron Microscopy

The morphology of LNPs was observed using transmission electron microscopy (TEM) with negative staining. LNP samples were concentrated to a final RNA concentration of 0.25 mg/mL using 10 kDa MWCO Amicon Ultra filter units. The concentrated samples were then prepared by applying 4 µL of LNPs to a glow-discharged 400-mesh copper grid with formvar carbon film (Electron Microscopy Sciences) for 1 minute. The grids were washed and negative stained with 0.1% uranyl acetate. The samples were imaged using a 100kV FEI Morgagni electron microscope equipped with a Gatan Orius SC200 CCD detector at a magnification of 22,000X (2.1 Å/pixel).

Small Animal Imaging

For *in vivo* live imaging, mice were injected with 200 µL saline or 1 mg/kg LNP. After 24 hours, mice were injected with 15 mg/kg d-luciferin and imaged for luminescent signal 20 minutes after the d-luciferin injection. Imaging was done using the IVIS® Lumina™ S5 high-throughput 2-D optical imaging system from the Small Animal Imaging Core at the University of Iowa. Radiance values were obtained from ROIs encompassing each mouse on medium-binning and compared between treatment groups.

Immunohistochemistry

Mice were injected via tail vein with 1 mg/kg LNP and livers were collected after 48 hours. Tissue was sectioned and stained using a CD68 antibody and DAPI nuclear stain by the University of Iowa Histology Research Laboratory and Comparative Pathology Laboratory.
